# Rapid genome modifications including chromosomal fusions and large-scale inversions are key features in Arctic codfish species

**DOI:** 10.1186/s13059-026-03975-6

**Published:** 2026-02-16

**Authors:** Siv N. K. Hoff, Marius F. Maurstad, Ole K. Tørresen, Robin Aasegg Araya, Paul R. Berg, Kim Præbel, Kjetill S. Jakobsen, Sissel Jentoft

**Affiliations:** 1 Centre for Ecological and Evolutionary Synthesis (CEES), Department of Biosciences, University of Oslo, Oslo, Norway; 2 Norwegian Institute for Water Research (NIVA), Oslo, Norway; 3 Centre of Coastal Research (CCR), University of Agder, Kristiansand, Norway; 4 Norwegian College of Fishery Science, Faculty of Biosciences, Fisheries and Economics, The Arctic University of Norway, Tromsø, Norway

**Keywords:** Comparative genomics, Gadiformes, Chromosomal rearrangements, Genome evolution, Transposable elements

## Abstract

**Background:**

Genome evolvability involves activation of transposable elements (TEs) that result in novel genomic rearrangements, including translocations, deletions, duplications, as well as larger structural reorganizations, such as chromosomal inversions and fusions. These genomic modifications contribute to raw genetic variability in which selection can act upon, and thus, promote local adaptation.

**Results:**

Using a comparative genomics framework combined with the generation of six chromosome-level gadid reference genomes, including the cold-water adapted polar cod (*Boreogadus saida*) and Arctic cod (*Arctogadus glacialis*), we uncover an array of larger and smaller chromosomal reorganizations within this lineage. For the two Arctic codfishes, we detect lineage-specific chromosomal fusions, i.e., five in polar cod vs. eight in Arctic cod, resulting in a reduced number of chromosomes found to be associated with their geographical distribution. For the same species, we identify a high number of partly overlapping chromosomal inversions where a majority (especially within breakpoint regions) display an overrepresentation of specific TE families, accompanied by signatures of high degree of conservation. We further demonstrate involvement of miniature inverted-repeat transposable elements (MITEs) in the expansion of the well-known antifreeze glycoprotein genes in polar cod, and that the three gene clusters were localized in association with chromosomal rearrangements on chromosomes 1, 2, and 14.

**Conclusions:**

We characterize how the Gadidae lineage has undergone massive genomic modifications — potentially via activation of TEs — throughout their evolutionary history, and particularly for the more Arctic species. These genomic reorganizations have likely played an important role in divergence processes and adaptation to freezing environmental conditions.

**Supplementary Information:**

The online version contains supplementary material available at 10.1186/s13059-026-03975-6.

## Background

Genomic architecture and organization — including chromosome numbers — vary massively across the tree of life [1, 2]. Most striking variation is reported within the Plantae kingdom [1, 2], mainly due to whole-genome duplications and/or polyploidization events, as well as via single chromosome changes such as chromosomal fusions and fissions [1, 2]. For closely related species, genome organization has in general been thought to be relatively conserved [3, 4]. However, in parallel with the developments in high throughput sequencing technologies, several studies have demonstrated how chromosomal reorganizations can occur over relatively short evolutionary timescales [5] and, in some cases lead to chromosomal speciation (reviewed in Damas et al. [6]) where examples include blind mole rats [7], rock-wallaby [8], and cichlid fish [9]. Large-scale genomic rearrangements, including chromosomal inversions, translocations, fission, and fusions, can be of high evolutionary importance — by linking allelic variants and/or genetic elements — leading to alterations in morphological or behavioral traits [10–13]. Ultimately, such genomic reorganizations can manifest within species, populations, and/or ecotypes as genetic polymorphisms, in which natural selection can act upon and mediate genomic divergence between species, populations, or ecotypes.

The key underlying mechanisms enabling such genomic modifications and reshufflings have in many species been associated with repetitive elements, which include interspersed transposable elements (TEs) as well as tandemly repeated regions, such as satellite DNA [14–23]. For instance, these shorter or longer satellite DNA sequences have been shown to play an important role in, e.g., chromosome pairing and recombination, and thus, replication slippage leading to copy-number changes of the satellite [24]. Moreover, the two groups of TEs — which are categorized based on their transposition mechanisms — have been shown to play a pivotal role in eukaryotic genome evolution, mainly via their ability to mobilize and replicate within the host genome, with the potential of carrying additional genomic sequences to the new insertion site [16, 25, 26]. More specifically, the retrotransposons (class I) include SINEs, LINEs, and LTR elements, which are known to propagate through a “copy-and-paste” mechanism involving reverse transcription and thus, dependent on an RNA intermediate, while the DNA transposons (class II) TEs employ a “cut-and-paste” strategy, i.e., not requiring an RNA intermediate [27–29]. In other words, TE activity can for instance facilitate the movement of genes [25], leading for instance to copy number variation of genes [30, 31], as well as affect chromosomal architecture by promoting recombination events between distant genomic regions, due to sequence homology [21–23, 32], and thought to contribute to large-scale genomic reshufflings such as chromosomal inversions and potentially fusions, with relevance for local adaptation.

Teleostei is the most diverse and species-rich infraclass of vertebrates with more than 30,000 species inhabiting numerous marine and freshwater habitats [33], where pronounced genomic modulations have been reported. These genomic modifications encompass variations in, e.g., genome organization and size, as well as chromosome numbers, features that might be associated with the remarkable diversity observed within this taxon [34–37]. Here, we could highlight the overall higher number of chromosomes as well as increased karyotype variation that have been observed in freshwater species compared to their marine counterparts [38, 39]. This genomic variability encountered in freshwater species has been suggested linked to local adaptation, and, i.e., the higher number of unique habitats, and thus, greater variability in environmental conditions encountered in freshwater vs. marine habitats, combined with well-defined barriers and lack of gene flow [38, 39]. One example is the huge karyotype variability — where the diploid chromosome numbers range from 16 to 50 — noted for the species within the genus *Nothobranchius*, which are uniquely adapted to the fluctuating environments of East African temporal savannah pools [39]. While genomic organization and chromosomal numbers tend to be more stable in marine species, some exceptions are reported. For the suborder Notothenioidei, an endemic group adapted to the freezing conditions of the Southern Oceans, multiple lineages have undergone extensive chromosomal reorganizations among closely related species [40–43]. Specifically, within the genera *Trematomus* [41] and *Notothenia* [42], unique chromosomal fusions and fissions have been reported, which resulted in reduced chromosome numbers for several of the species.

**Fig. 1:**
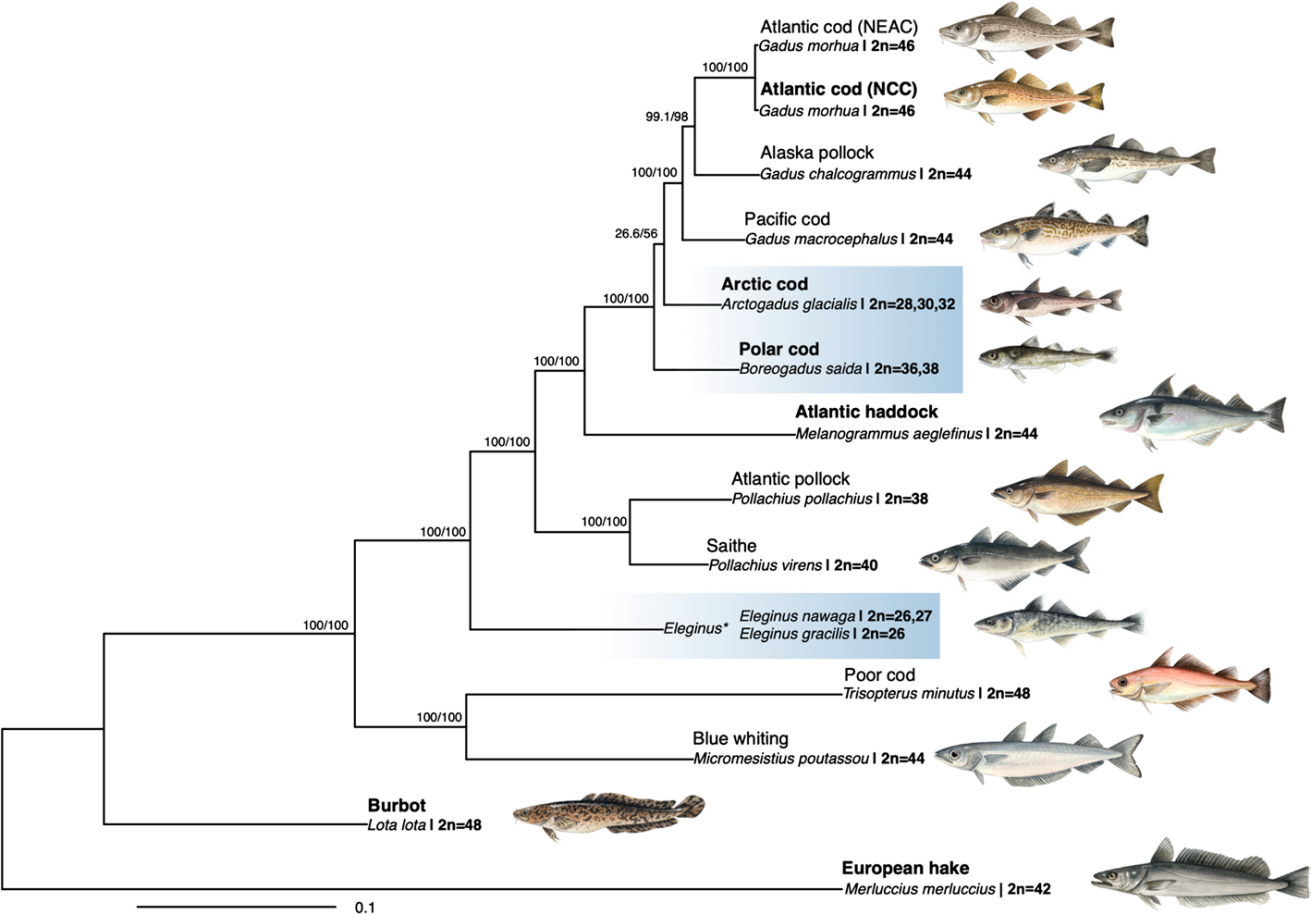
Karyotype variability and phylogenetic relationships among Gadiformes. Phylogenetic relationship for 13 Gadiform species and ecotypes generated by IQ-TREE2 v2.2.0 [59], using the 13 mitochondrial protein-coding genes, for which the karyotype/chromosome numbers are available (see Additional file 1, Table S1 for more details). The six species genome sequenced in the present study are marked in bold. For Atlantic cod, we have included both the non-migratory Norwegian coastal cod (NCC) as well as the migratory Northeast Arctic cod (NEAC). The Arctic residing codfishes are marked in blue. *Eleginus gracilis is used as a representative for both Saffron cod (*Eleginus gracilis*, 2n = 26) and Navaga (*Eleginus nawaga*, 2n = 26–27). Illustrations made by Alexandra Viertler

In the present study, we aimed at taking a closer look at genomic architecture(s) of the northern gadids (codfishes), a family within the order of Gadiformes, which includes species primarily distributed across the northern Atlantic, including the freezing Arctic Oceans and the Northern Pacific [44]. Although Arctic conditions are not as extreme as in Antarctica, organisms in both regions face similar environmental challenges, such as freezing seawater and thick ice covers [45]. For instance, similar to the notothenioids, cold water adapted gadids such as the polar cod (*Boreogadus saida*) and Arctic cod (*Arctogadus glacialis*) display an extended repertoire of antifreeze glycoproteins (AFGP) compared to their temperate counterparts, i.e., enabling these species to cope under extreme freezing conditions [46, 47]. Moreover, inter- and intraspecies chromosome number variation has been reported in previous studies within the gadid family (see Fig. 1; Additional file 1, Table S1). Among the cold-water adapted species residing in the Arctic and sub-Arctic regions [48, 49], fewer chromosomes have been identified compared to the presumed ancestral teleost karyotype (*n* = 24–26) [50–52]. The reduction in chromosome number seen both in the Antarctic notothenioids and Arctic codfishes is intriguing and raises questions about the adaptive significance of chromosomal fusions in species inhabiting high-latitude oceans. For Arctic cod, observed population-level karyotype variation seems to follow a latitudinal cline, supporting the idea of chromosomal reduction being advantageous in cold-water conditions [49]. Moreover, high numbers of chromosomal inversions have been reported for many gadids, and are likely to play important roles in the separation of co-occurring cryptic ecotypes [53, 54], as well as for local adaptation to different environmental conditions [55–58].

To further our insight into the genomic composition and reorganizations within the gadids (see [53–58]), we here took full advantage of the newly generated chromosome-level genome assemblies for Arctic cod (*Arctogadus glacialis*), polar cod (*Boreogadus saida*), non-migratory Atlantic cod (*Gadus morhua*), i.e., Norwegian coastal cod (NCC), Atlantic haddock *(Melanogrammus aeglefinus),* burbot (*Lota lota*), and European hake (*Merluccius merluccius*) using a comparative genomic approach. Combining these resources with a subset of the population data from Arctic cod and polar cod [53, 54], we identify several lineage-specific chromosomal fusions as well as a high number of partly overlapping chromosomal inversions present in the two Arctic codfishes. Intriguingly, a high degree of conservation was identified for some of the overlapping inversions (especially within the breakpoint regions), which was accompanied by an accumulation of TEs. Additionally, we characterize three well-known *afgp* gene clusters in polar cod, where involvement of miniature inverted-repeat transposable elements (MITEs) seems to play an important role in their formation. Lastly, we reconstruct phylogenetic trees using Benchmarking Universal Single-Copy Orthologs (BUSCO) and mitochondrial genes in an effort to elucidate the relationship of Arctic cod within the Gadidae family.

## Results

### Generation of chromosome-anchored genome assemblies for six codfish species

The generation of primary assemblies using long-read Pacific Biosciences (PacBio) data ranged from 42.42 to 98.97 Gb of data (see Additional file 1, Table S2 for more information) and subsequently scaffolding with the linked read data, e.g., 10X and/or Illumina Hi-C, yielded highly contiguous chromosome-level genome assemblies for all six species: polar cod, Arctic cod, Atlantic cod (NCC), Atlantic haddock, burbot, and European hake. The number of super-scaffolds obtained from each assembly utilizing Hi-C data matched the karyotype numbers earlier reported for five of the species (Table 1; Additional file 1, Table S1; Additional file 2, Fig. S1), except for the genome assembly of burbot, which resulted in 23 chromosome-length scaffolds, where *n* = 24 have been reported cytogenetically [60, 61] (see Additional file 3 for more information). The genome assemblies ranged from ~ 538 Mb across 2223 to ~ 679 Mb across 4087 scaffolds for burbot and Atlantic cod (NCC), respectively (Table 1). All putative chromosomes assembled for the six species were larger than 10 Mb in size, and the majority of the assembled sequences (> 93.13% for all assemblies) were placed within the chromosome-length scaffolds (Table 1). Gene completeness was scored to > 90.1% (complete BUSCOs) for the genome assemblies (Table 1). Moreover, the completeness of the PacBio reference genome assemblies, in terms of intra-chromosomal synteny, was confirmed by comparing the reference genome assemblies for polar cod and Arctic cod to their respective Oxford Nanopore Technologies (ONT) draft genome assemblies (see Additional file 4; Additional file 2, Figs. S2 and S3 for more details). The gene annotation of the genome assemblies resulted in total predicted genes ranging from 21,807 in burbot to 23,607 in Atlantic cod (NCC), and the BUSCO gene completeness score ranged from 90.3% for polar cod to 95.5% for burbot. For a full overview of all species (see Table 1; Additional file 1, Table S3).

**Table 1:** Assembly metrics of final genome assemblies and annotation for the six species

Species/	Arctic cod	Polar cod	Atlantic cod (NCC)	Atlantic haddock	Burbot	European hake
Statistics						
**Genome assembly metrics**						
Total scaffolds size (bp)	633,919,102	585,721,513	679,525,213	639,457,590	537,831,372	646,507,975
N scaffolds	2875	2300	4087	1310	2223	830
N chromosome length scaffolds*	15	18	23	22	23	21
N50 scaffold lengths (bp)	49,001,811	28,087,346	27,969,116	26,664,727	22,427,591	28,921,266
N contigs	8221	8646	8503	2333	4307	6667
N50 contig length (bp)	193,655	126,712	271,179	988,607	659,811	143,622
Assembly in scaffolded contigs (% bp)	96.8	95.9	95.5	93.13	97.9	96.12
Base constitution (scaffolds)**	27.00, 22.95, 22.96, 27.02,0.08	26.97, 23.00, 22.99, 26.94,0.10	27.08, 22.90, 22.92, 27.04,0.06	26.96,23.03,23.03,26.94,0.03	27.09,22.68,22.68,27.16,0.38	27.61, 22.17, 22.12, 27.65,0.45
BUSCO completeness final assembly***	C:91.7%[S:90.5%,D:1.2%],F:1.5%,M:6.8%	C:90.1[S:88.8%,D:1.3%],F:1.5%,M:8.4%	C:93.5[S:91.8%,D:1.7%],F:0.7%,M:5.8%	C:90.4[S:88.8%,D:1.6%],F:1.5%,M:8.1%	C:94.9[S:93.9%,D:1.0%],F:0.6%,M:4.5%	C:91.4[S:88.4%,D:3.0%],F:0.8%,M:7.8%
**Gene annotation metrics**						
BUSCO completeness predicted genes***	C:92.6[S:90.9%,D:1.7%],F:2.2%,M:5.2%	C:90.3[S:88.2%,D:2.1%],F:2.9%,M:6.8%	C:94.6[S:92.5%,D:2.1%],F:1.5%,M:3.9%	C:94.5[S:93.0%,D:1.5%],F:1.2%,M:4.3%	C:95.5[S:94.0%,D:1.5%],F:1.0%,M:3.5%	C:91.2[S:86.9%,D:4.3%],F:1.9%,M:6.9%
N predicted genes	22,366	21,810	23,607	22,707	21,807	22,070
Mean gene length (bp)	14,400	13,585	14,552	14,532	13,550	14,692

*Total BUSCO groups searched: 3640. The lineage dataset is: actinopterygii_odb10 database*

*Genes with functional prediction either have a functional annotation from InterProScan or name from comparisons with UniProtKB/SwissProt*

^*^Number of scaffolds > *10 M nt*

^**^%A, %C, %G, %T, %N

^***^C: Complete; S: Complete and single copy; D: Complete and duplicated; F: Fragmented; M: Missing

Complete mitogenomes were successfully assembled for the six species included and utilized for downstream phylogenetic analyses (Additional file 3; Additional file 2, Figs. S4–S6 for more details on mitogenomes).

### Macro-synteny and identification of chromosomal fusions and translocations

**Fig. 2:**
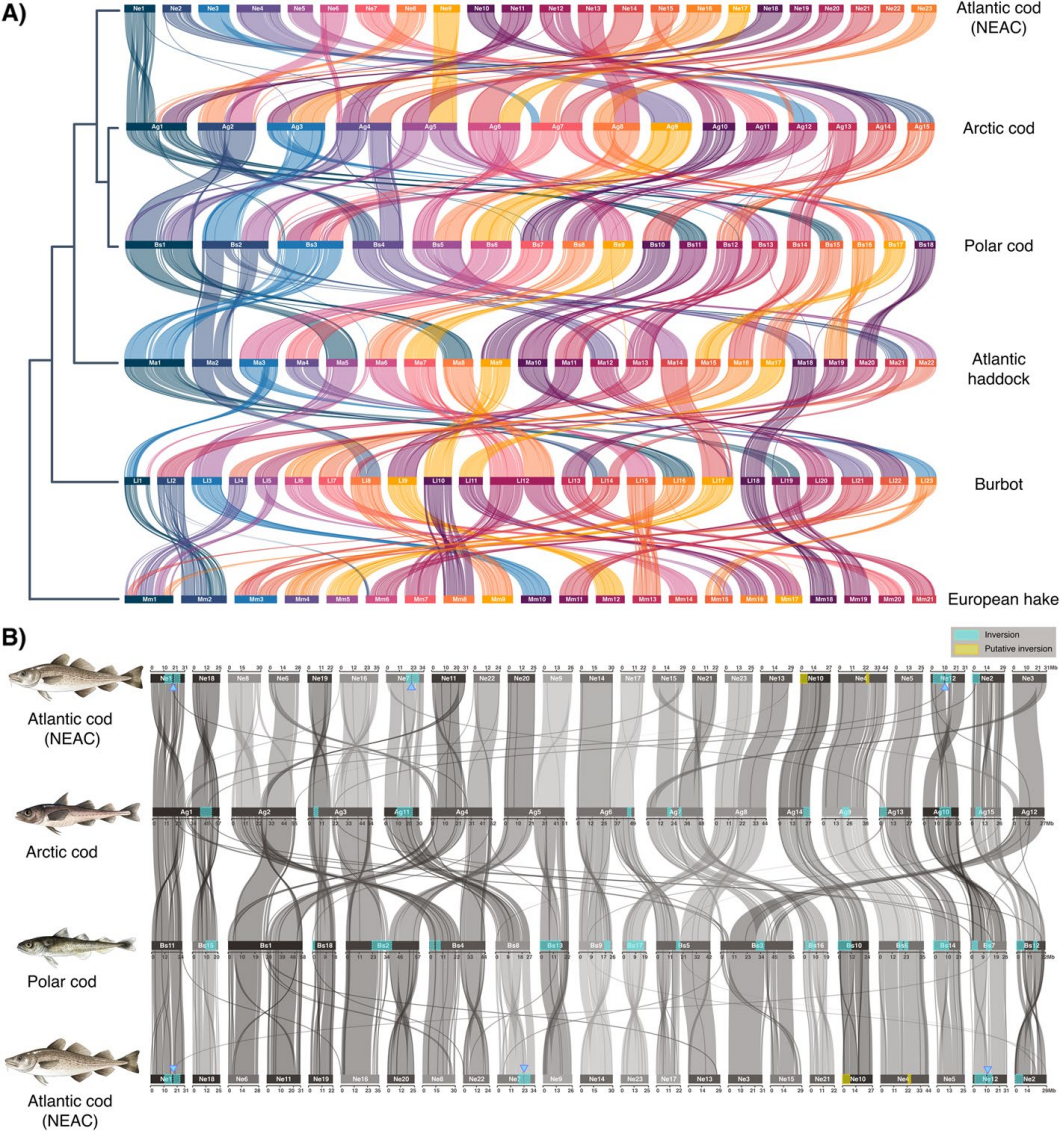
Chromosomal synteny among Gadiform species.** A** Chromosomal synteny inferred by gene order using the programs MCScanX [63] and SynVisio [64] among the six codfishes. Chromosomes are ordered by size from largest to smallest, except for Atlantic cod. Phylogenetic relationships redrawn as a cladogram based on multispecies coalescent (MSC) species tree inferred using Astral-III [65] of 1939 BUSCO gene trees shared between all taxa. **B** Chromosomal synteny between the two Arctic codfishes as well as Atlantic cod, and a schematic overview of previously identified intraspecies polymorphic inversions (marked with turquoise squares) in Atlantic cod [55–58, 66], polar cod [53], and Arctic cod [54]. The putative inversions identified in the present study in Atlantic cod are marked in yellow

To explore the interspecies macro-syntenic relationship between Atlantic cod (using the newly released version of the migratory Northeast Arctic cod (NEAC) genome assembly, i.e., gadMor3.0, see [62] for more details) and the five other codfish species presented in this study (Table 1), in-depth gene order comparisons were conducted. The analyses revealed an overall conserved intra-chromosomal macro synteny between the species for a majority of the chromosomes. However, some larger inter-chromosomal rearrangements such as fusions and translocations were detected (Fig. 2; Additional file 4; Additional file 2, Figs. S7–S9 for more details). The largest reorganizations were detected in Arctic cod and polar cod (Fig. 2A and B), which included multiple species-specific chromosomal fusions. More specifically, we detected eight fused chromosomes in Arctic cod (with an average size of ≥ 44 Mb) and five fused chromosomes in polar cod (with an average size of ≥ 42 Mb), resulting in a reduction in chromosome number, i.e., *n* = 15 and *n* = 18 in Arctic cod and polar cod, respectively (Fig. 2A and B; Additional file 1, Table S4) when compared with e.g. Atlantic cod (*n* = 23). These fusions were firmly confirmed by i) the detected intra-chromosomal Hi-C contact points and ii) the assemblage of ONT data from different individuals for both Arctic cod and polar cod yielded the same fusions as the PacBio reference genomes. Additionally, a closer inspection of one of the fused chromosomes in Arctic cod further demonstrated how the ONT reads were spanning the fusion (see Additional file 4; Additional file 2, Fig. S10 for more details). It should also be noted that these fusions were found to correspond primarily to two homologous chromosomes when compared with any of the other codfishes (Fig. 2A and B; Additional file 1, Table S4). Furthermore, none of the fusions were found to be shared between Arctic cod and polar cod.

The Hi-C contact points also uncovered the putative location of the centromere for most of the chromosomes in the majority of the species, except for the burbot, where the contact point did not unravel the centromere to the same degree as in the other species (see Additional file 2, Fig. S1). For instance, for most of the fused chromosomes in Arctic cod and polar cod, the centromeres appear to be centrally located, except for chromosome 3 (Bs3) in polar cod and chromosome 2 (Ag2) in Arctic cod (Additional file 2, Fig. S1D and E for Arctic cod and polar cod, respectively).

The analyses also unraveled a multitude of both smaller and larger inter-chromosomal translocations when comparing polar cod and Arctic cod vs. Atlantic cod. For Arctic cod, the majority of these were found within the central regions of the fused chromosomes. For instance, for the fused chromosome Ag1 in Arctic cod, which is primarily homologous to two chromosomes in Atlantic cod (Ne1 and Ne18; see "Methods" for the renaming of the Atlantic cod chromosomes), a central region (~ 25–34 Mb) was found to be homologous to the tail end of a third chromosome in Atlantic cod (Ne15) (Fig. 2B).

For the fused chromosome Ag2 in Arctic cod, which is mainly homologous to Ne8 and Ne6 in Atlantic cod, the central region (~ 22–30 Mb) was found to correspond to a smaller region at the end of Ne11 in Atlantic cod (Fig. 2B; Additional file 1, Table S4). Similarly, the fused chromosome Ag4, primarily homologous to Ne11 and Ne22 in Atlantic cod, displayed a region at the beginning of the chromosome (~ 1–5 Mb) corresponding to a chromosomal segment at the beginning of Ne6 in Atlantic cod. Additionally, the fused chromosome Ag7 was found to mostly correspond to Ne15 and Ne21, whereas the first part of the chromosome (~ 1–10 Mb) was found to be homologous to a chromosomal region in the tail end of Ne3 in Atlantic cod. For Ag12, which was primarily homologous to Ne3, a smaller region at the beginning of the chromosome (~ 1–5 Mb) corresponds to the first part of Ne15 in Atlantic cod (Fig. 2B; Additional file 1, Table S4).

When comparing polar cod with Atlantic cod, we also here detected a high number of translocations, but these were in general located at the distal ends of the chromosomes, i.e., the telomeres, and not at the central part of the chromosomes. First, we could highlight that for two of the fused chromosomes, i.e., Bs2 and Bs4, there are seemingly a reorientation of both the distal ends for one of the fused homologous chromosomes when comparing to Atlantic cod, i.e., Ne20 and Ne22, respectively (see Fig. 2B). For another of the fused chromosomes, Bs5, we notice a rearrangement involving one of the distal ends of the homologous chromosome in Atlantic cod, i.e., Ne13, to have shifted its position to be located in the telomere region of the new fused chromosome (see Fig. 2B). Similarly, for some of the non-fused chromosomes, we also detect reallocations on the ends of the chromosomes, e.g., Bs12 and Bs13 homologous to Ne2 and Ne14 in Atlantic cod. Additionally, we found that the fused chromosome Bs1, mainly homologous to Ne6 and Ne11 in Atlantic cod, displayed a region centrally located on the chromosome (~ 25–29 Mb) corresponding to the tail end of Ne8, Ne13, and the beginning of Ne22 in Atlantic cod. For Bs1, we also detected that two smaller regions in the distal ends of the two fused chromosomes, i.e., Ne6 and Ne11, respectively, are translocated to the opposite distal ends of the fused chromosome. Finally, in the fused chromosome Bs3 in polar cod, mainly homologous to Ne3 and Ne15 in Atlantic cod, an intra-chromosomal translocation of the end region on Ne15 (~ 25–29 Mb) to the beginning of Bs3 (~ 1–10 Mb) has seemingly occurred (Fig. 2B). However, this reshuffling could also be explained (but less likely) by an insertion of Ne3 into Ne15 with additional subsequent translocations (see Additional file 4; Additional file 2, Fig. S9C and D).

### Intraspecies chromosomal synteny and detection of novel chromosomal inversions between Atlantic cod ecotypes

**Fig. 3:**
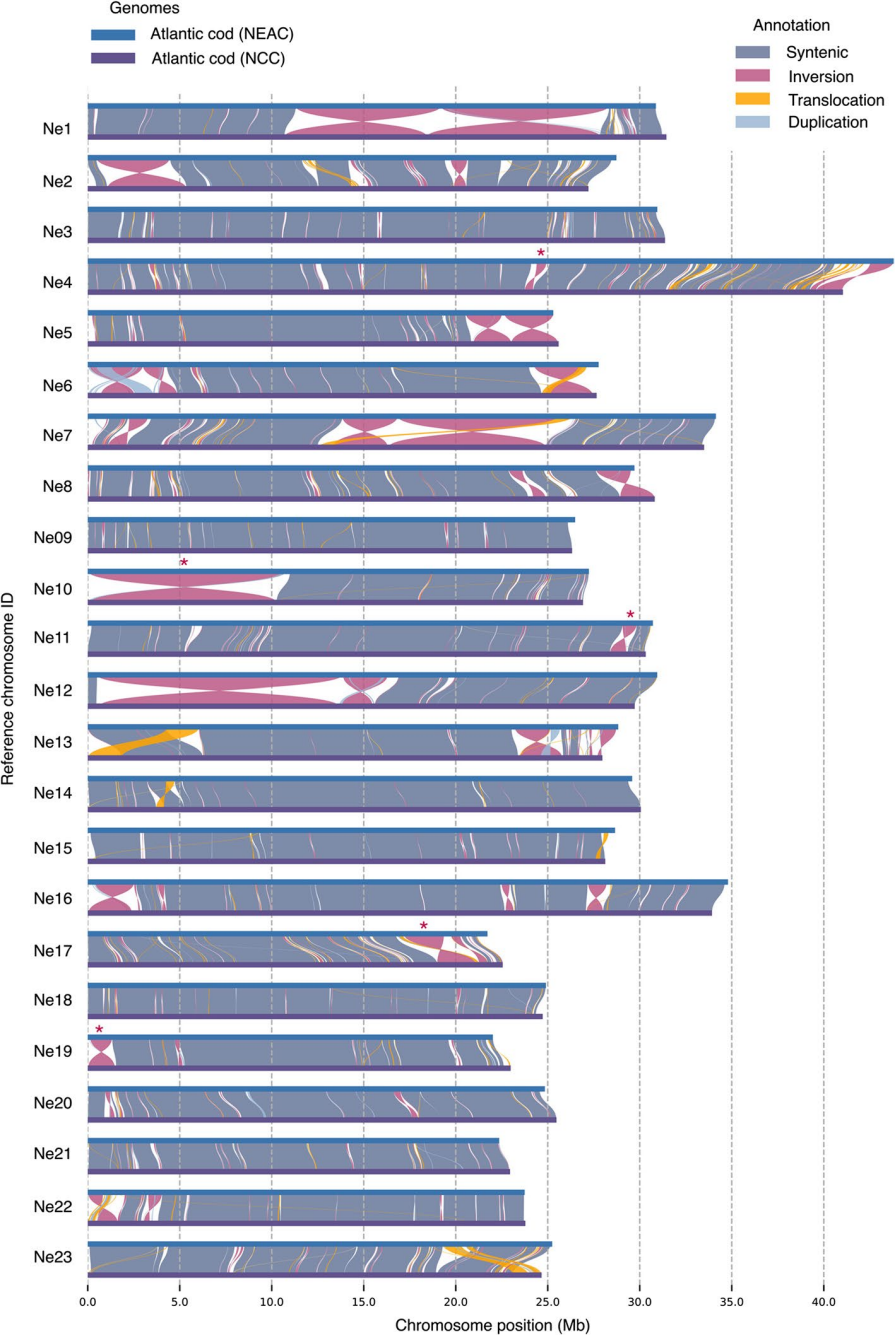
Chromosomal synteny between the two Atlantic cod ecotypes, the migratory Northeast Arctic cod (NEAC) and the non-migratory Norwegian coastal cod (NCC). Chromosomal synteny inferred using SyRI [67]. Red stars denote putative chromosomal inversions identified in the present study, where signals of elevated LD have previously been reported when populations of Atlantic cod have been examined [55, 57, 71]

**Fig. 4:**
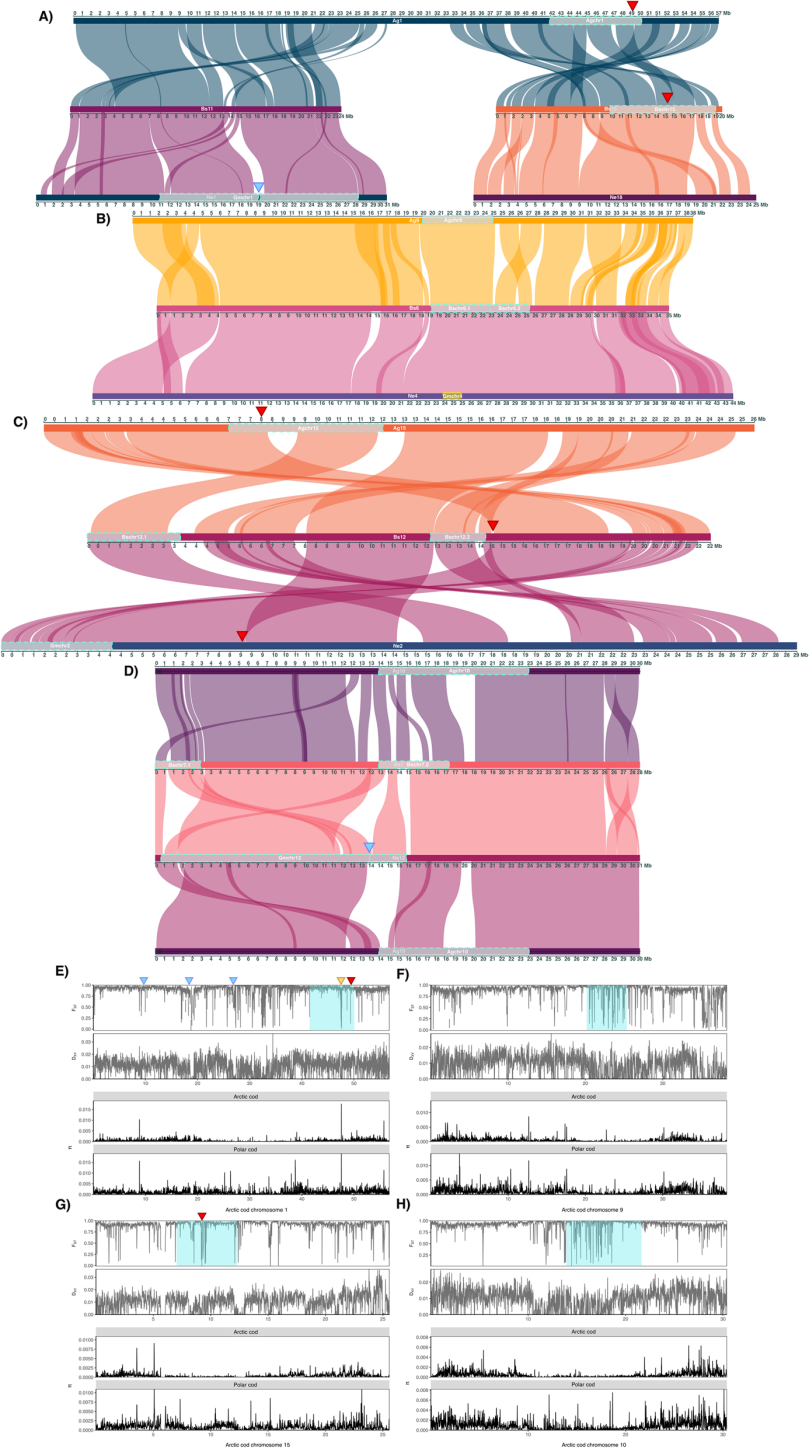
Chromosomal synteny, genetic diversity, and differentiation between Arctic cod and polar cod, within inversion regions. **A**-**D** Pairwise chromosomal syntenies for a selection of chromosomes where intraspecies inversions overlap between Arctic cod, polar cod, and/or Atlantic cod (NEAC), for (**A**) Ag1—Bs11 & Bs15—Ne1 & Ne18, (**B**) Ag9—Bs6—Ne4, (**C**) Ag15—Bs12—Ne2, and (**D**) Ag10—Bs7—Ne12—Ag10. Intraspecies inversions are marked in turquoise and named according to the species and the chromosome where they are located, i.e., Agchr, Bschr, and Gmchr for Arctic cod, polar cod, and Atlantic cod, respectively. One putative inversion in Atlantic cod is marked in yellow (on Ne4). Approximate breakpoint region between two subsequent double inversions in Atlantic cod marked with blue triangles. Locations of hemoglobin gene clusters associated with inversions are marked with red triangles, where LA and MN clusters are located in (**A**) and (**B**), respectively. **E**–**H** Intraspecies genetic differentiation and nucleotide diversity estimated by pixy [72] between Arctic cod and polar cod using the Arctic cod genome assembly as a reference, for chromosomes 1, 9, 15, and 10. The population data includes Arctic cod (*N* = 14) and polar cod (*N* = 14) collected in Tyrolerfjorden, Greenland, and the Barents Sea, respectively. Blue triangles highlight approximate breakpoint regions of the double inversion in Atlantic cod on Ne1, in homologous regions of Arctic cod Ag1, yellow triangle indicates the position of *fbxl5* gene (connected to homeostasis [73]), and red triangles specify the position of hemoglobin gene cluster locations on Arctic cod Ag1 and Ag15. Turquoise squares indicate intraspecies inversions in Arctic cod

The generation of the chromosome-level genome assembly for the non-migratory NCC enabled in-depth inspection of the chromosomal synteny with the already released version of the genome assembly of the migratory NEAC [62] (gadMor3.0, NCBI refseq assembly: GCF_902167405.1). By the comparative analyses (using SyRI [67]), we identified several smaller and medium sized chromosomal inversions (Fig. 3), in addition to the already known chromosomal inversions on LG01, LG02, LG07, and LG12 [55–58, 66], here synonymous with Ne1, Ne2, Ne7, and Ne12 (Fig. 3). Of the already characterized chromosomal inversions, we discover that the inversions previously identified on Ne7 and Ne12 are both made up of double inversions (see Fig. 3). For the inversion on Ne7, a recent study has reported that this inversion likely consists of larger haploblocks with linked single-nucleotide polymorphisms (SNPs) [68], possibly reflecting the double inversion identified in the present study. Furthermore, for the inversion on Ne12, discrepancies have been reported for the length of the inversion, with the latter breakpoint either being located at around 13.5 Mb [10, 55, 69] or alternatively around 16 Mb [58]. Here, we provide support that this inversion is a double inversion, and the length variation previously reported may reflect differences in frequency of the genotypes of the second, smaller inversion located at ~ 13.5–16 Mb on Ne12 among the different populations examined (see Fig. 3). We provide further support for the previously identified double inversion on Ne1 [70], with our analyses delineating two subsequent inverted segments (Fig. 3). Additionally, we discovered one putatively larger inversion on Ne10 (Fig. 3), not yet discovered by population genome-wide datasets. It should be mentioned, however, that within this chromosome, patterns of high linkage disequilibrium (LD) have been detected, especially in comparisons where Baltic and Canadian populations were included [55, 57]. For some of the smaller and medium-sized inversions mentioned above, we here want to highlight the ones on Ne11, Ne17, and Ne19 (shown with red stars in Fig. 3), which are found to be overlapping with genomic regions of high LD in multiple population genomic studies [55, 57, 71]. Lastly, we highlight one smaller putative inversion detected on Ne4 (Figs. 2, 3C, and 4C), which seems to be partly overlapping with the species-specific inversions detected in polar cod and Arctic cod (Figs. 2B and 4C; see description below for more information). To confirm that the putative inversions on Ne4, Ne10, Ne11, Ne17, and Ne19 are polymorphic, population genetic analyses are needed.

### Genomic differentiation and detection of overlapping chromosomal inversions

Inspecting the genomic locations of the reported intraspecies chromosomal inversions in Atlantic cod [55–58, 66], polar cod [53], as well as Arctic cod [54], we detected in total eight partly overlapping inversions between the two Arctic species, where two of these were also found to be partly overlapping with two of the inversions detected in Atlantic cod (Fig. 4A–D; Additional file 1, Table S5; Additional file 2, Fig. S11). Further, we discovered two additional partly overlapping inversions, one detected between Arctic cod and Atlantic cod and one between polar cod and Atlantic cod (Fig. 2B). Here, it should be noted that the highest degree of overlap was seemingly coupled to the breakpoint regions of the inversions.

To enable a further characterization of the inversions, as well as the overlapping breakpoint regions, a more in-depth validation and accurate localization were performed. For this analysis, we took advantage of both i) the ONT draft assemblies as well as ii) the long ONT reads from the sequencing of both polar cod and Arctic cod. First, when comparing the two genome assemblies (PacBio vs. ONT) stemming from two different specimens, we detected 11 out of the total 20 inversions reported in Hoff et al. [53] for polar cod, and one inversion reported in Maurstad et al. [54] for Arctic cod. For these inversions, a further validation in terms of size and location (for more information, see Additional file 1, Tables S6 and S7) was performed. It should be noted that all of the inversions in both species have been confirmed by population data and shown to be polymorphic within the species (see [54] and [53]), and the probability of detecting all of the inversions is unlikely when only comparing two reference genome assemblies from two different individuals. For the rest of the inversions not detected between the genome assemblies, we used the breakpoints defined in Hoff et al. [53]. As a last confirmation, the inspection of ONT reads that were mapped back to the Arctic cod PacBio assembly within the region of the identified inversion on chromosome 6 between these assemblies revealed mapping patterns indicative of putative inversion breakpoints, with an abrupt mapping break in the first breakpoint region (Additional file 4; Additional file 2, Fig. S12 for more details). Taken together, our results support that the inversions detected by population data are indeed true inversions.

From the population data utilized in this study (i.e., Arctic cod and polar cod samples collected in Tyrolerfjorden, Greenland [*N* = 14] and the Barents Sea [*N* = 14], respectively) the genomic differentiation estimates and/or sequence similarity along chromosomes between the two Arctic species revealed overall high degree of differentiation as expected [54, 74], with a baseline F_ST_ averaging 0.8–0.9 (see Fig. 4E–H; Additional file 2, Fig. S13). However, we also identified local stretches along the chromosomes where lower values of F_ST_ (below 0.5), as well as close to zero values of genetic divergence (D_XY_) (Fig. 4E–H; Additional file 2, Fig. S13) were observed. Specifically, such regions were localized in connection to the partly overlapping inversions and/or in the breakpoint regions identified (Fig. 4A–D). Moreover, the estimated nucleotide diversity (π) was on average lower in Arctic cod than in polar cod (Fig. 4E–H; Additional file 2, Fig. S13), i.e., most likely a result of reference bias, since we here used Arctic cod as a reference genome for the variant calling [54].

When comparing the last part of the fused chromosome Ag1 with the corresponding homologous chromosomes in the two other species, i.e., Bs15 in polar cod and Ne18 in Atlantic cod, we found the species-specific chromosomal inversions in polar cod (named Bschr15) and Arctic cod (named Agchr1) to be partly overlapping (Fig. 4A). Intriguingly, one of the hemoglobin gene clusters (the LA cluster) was identified to be located at the very edge of the inversion in Arctic cod, while found to be located within the inversion in polar cod (see Fig. 4A, where the localization of the LA cluster is defined with a red triangle). Moreover, comparing the first part of the fused chromosome of Ag1 with the corresponding homologous chromosomes, i.e., Bs11 and Ne1, we detected no presence of any overlapping inversions between the Arctic species. For Atlantic cod, however, this is the chromosome (Ne1) where the large inversion discriminating between the non-migratory and migratory behavior is localized (named Gmchr1) [10, 55, 57, 70]. However, for the same region using Arctic cod chromosome 1 (Ag1) as the reference, by pairwise comparisons between polar cod and Arctic cod, we identified several stretches with F_ST_ estimates lower than 0.5, i.e., indicating a higher degree of sequence similarity between the two species in those regions (Fig. 4E). These low F_ST_ stretches were i) overlapping with the three breakpoint regions of the identified double inversion in Atlantic cod Ne1 [70] (Figs. 3, 4A and E) and ii) the breakpoint regions of the partly overlapping inversion identified between polar cod and Arctic cod (Fig. 4A and E). Inside the latter inversion, we also detected lower estimates of F_ST_ as well as high values of π for the region harboring the F-box and leucine-rich repeats protein 5 (*fbxl5*) gene in Arctic cod, which has been linked to iron homeostasis [73].

For the homologous chromosomes Ag9, Bs6, and Ne4, we identified species-specific inversions that overlapped between the two Arctic species as well as with a smaller putative inversion detected in Atlantic cod within this study (Fig. 4B and F). Moreover, the first breakpoint region for the overlapping inversions in polar cod (named Bschr6.1 and Bschr6.2) and Arctic cod (named Agchr9) is homologous with a high LD region (i.e., a putative inversion, named Gmchr4) detected in Atlantic cod on Ne4 [55, 57, 71]. Calculations of F_ST_ and D_XY_ between polar cod and Arctic cod along chromosome 9 (Ag9) of Arctic cod uncovered multiple regions with very low F_ST_ estimates (lower than 0.25) compared to surrounding regions. Here, the stretches of low F_ST_ overlap entirely with the overlapping inversions between the two Arctic species. Moreover, calculations of D_XY_ display a putatively similar pattern, with lower estimates over the same region (Fig. 4F).

For the homologous chromosomes Ag15, Bs12, and Ne2, the inversion detected in Arctic cod (named Agchr15) was found to be partly overlapping with one of two chromosomal inversions detected in polar cod (named Bschr12.1) (Fig. 4C). In addition, due to intra-chromosomal translocations, the Arctic cod inversion (Agchr15) seems to have an overlapping breakpoint region with the second inversion detected in polar cod (named Bschr12.2). Intriguingly, this genomic region harbors the second hemoglobin cluster (the MN cluster), which is localized outside of the inversion detected in Atlantic cod (named Gmchr2), at the end breakpoint region of the inversion detected in polar cod, and inside the inversion detected in Arctic cod (Fig. 4C, the MN cluster is marked with a red triangle). Moreover, estimates of F_ST_ and D_XY_ indicate higher degree of sequence similarity at the breakpoint regions of the inversion found in Arctic cod, as well as for the MN cluster (Fig. 4G, indicated with a red triangle).

When comparing the homologous chromosomes Ag10, Bs7, and Ne12, we found that the species-specific inversions detected, i.e., the putative double inversion in Atlantic cod (named Gmchr12) (Fig. 3) vs. one inversion in Arctic cod (named Agchr10) and two inversions in polar cod (named Bschr7.1 and Bschr7.2), are seemingly all overlapping (Fig. 4D). In addition, intra-chromosomal translocations have further led to shared local genomic regions between the inversions, e.g., a region within the inversion on Ag10 in Arctic cod, seems to translocate and make up the first breakpoint region in the first of the two inversions identified in polar cod (Fig. 4D), whereas the end breakpoint of the first inversion of the double inversion in Atlantic cod makes up the latter part of this first inversion in polar cod (see Fig. 4D). Moreover, the overlapping genomic regions between the inversions in Arctic cod and polar cod, i.e., the latter part of the inversion detected in Arctic cod, exhibited low estimates of F_ST_ between the two Arctic species compared to surrounding regions (Fig. 4H).

**Fig. 5:**
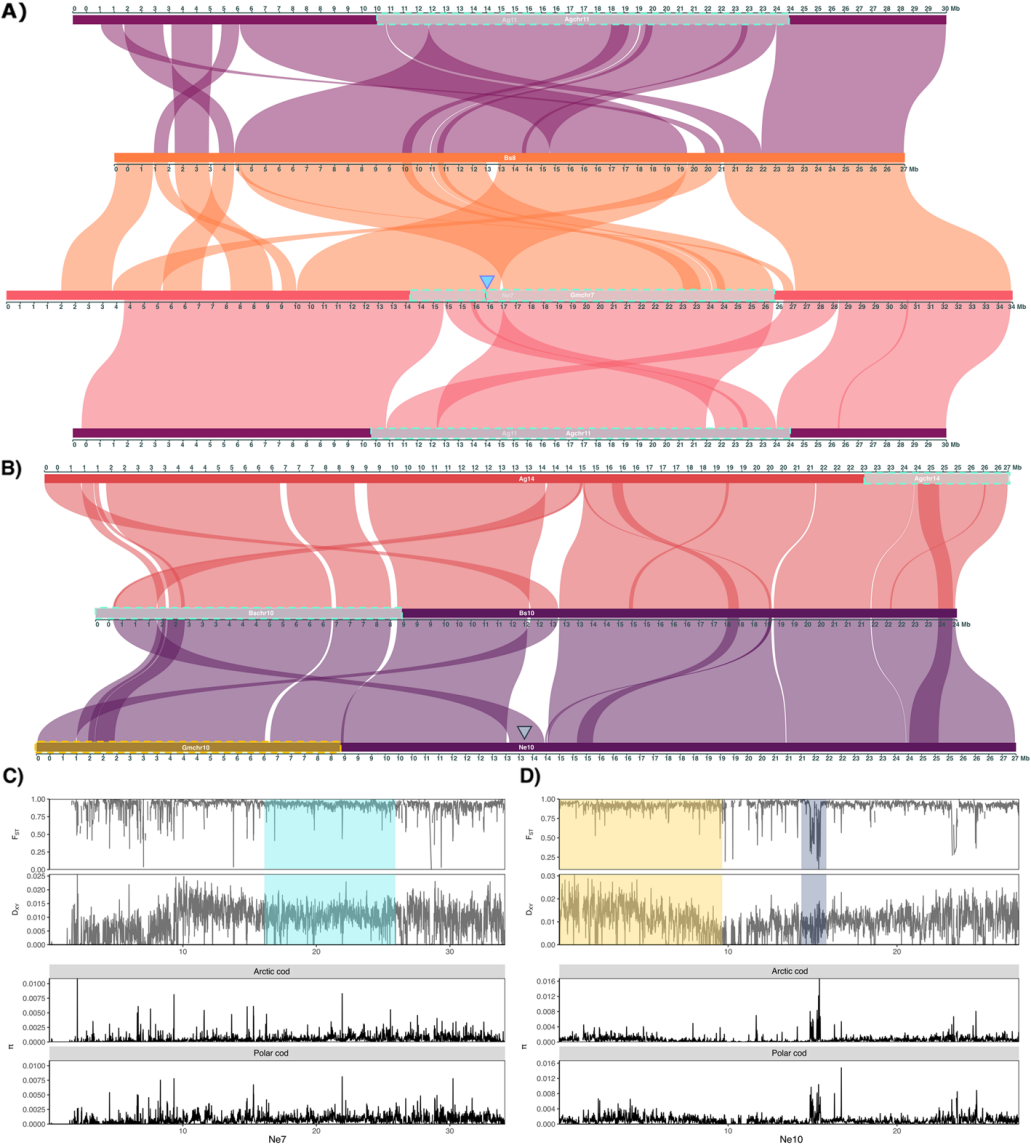
Chromosomal synteny, genetic diversity, and differentiation within inversion regions between Atlantic cod, Arctic cod, and polar cod. Chromosomal synteny between the Arctic codfishes, and Atlantic cod (NEAC), displaying (**A**) Ag11—Ne7—Bs8—Ag11 and (**B**) Ag14—Bs10—Ne10. The boundary between the double inversions in Atlantic cod on Ne7 is marked with a blue triangle. Grey triangle marks a homologous region between Atlantic cod and polar cod that makes up the first breakpoint in the intraspecies inversion found in polar cod Bs10, which is translocated to the middle of Ne10 in the between-species comparison. **C** and **D** Interspecies genetic differentiation and nucleotide diversity were calculated using pixy [54] between Arctic cod and polar cod using the Atlantic cod (NEAC) reference genome for Ne7 and Ne10, respectively. Gray colored box corresponds to the region denoted by a grey triangle in B) and indicates the homologous region between Atlantic cod and polar cod that makes up the first breakpoint in the intraspecies inversion found in polar cod Bs10. The intraspecies polymorphic inversion region in Arctic cod (largely overlapping with the inversion in Atlantic cod (NEAC) on Ne7) is visualized with turquoise square, while the putative inversion on Ne10 in Atlantic cod (NEAC) is marked in yellow. Intraspecies inversions are named according to the species and the chromosome where they are located, i.e., Agchr, Bschr, and Gmchr for Arctic cod, polar cod, and Atlantic cod, respectively

For the homologous chromosomes Ag11, Bs8, and Ne7, an inversion detected in Arctic cod (named Agchr11) was found to overlap to some extent with the double inversion in Atlantic cod (named Gmchr7), where the major differentiation seems to be linked to translocation of the breakpoint regions in Arctic cod (Fig. 5A and C). For this region, however, no signals of sequence similarity (F_ST_ nor D_XY_) were detected between polar cod and Arctic cod, except for a smaller region co-localizing with the first breakpoint region in Arctic cod that is translocated in Atlantic cod (Fig. 5A and C).

The final comparison conducted between homologous chromosomes was between polar cod Bs10 and Atlantic cod Ne10. In polar cod, this chromosome harbors a polymorphic inversion (named Bschr10) that overlaps to some extent with the inversion detected when comparing the genome assembly for the two Atlantic cod ecotypes, the non-migratory NCC with the migratory NEAC (inversion named Gmchr10) (see Figs. 3 and 5B). In Arctic cod, an inversion has been detected on Ag14 (homologous to Bs10 and Ne10 and named Agchr14) at the end of the chromosome; however, this inversion was not found to overlap with the inversions found in polar cod (Bschr10) and Atlantic cod (Gmchr10) (Fig. 5B). Inspecting the estimates of F_ST_ and D_XY_ within this region between polar cod and Arctic cod, we detect lower values of F_ST_ for the stretches that are presumably overlapping with breakpoint regions of this inversion, as well as lower estimates of D_XY_ (~ 9 Mb for the second breakpoint and 13–14 Mb for the translocated area making up the first breakpoint, Fig. 5D).

### Repeat density patterns associated with chromosomal fusions and inversions in the Arctic codfishes

**Fig. 6:**
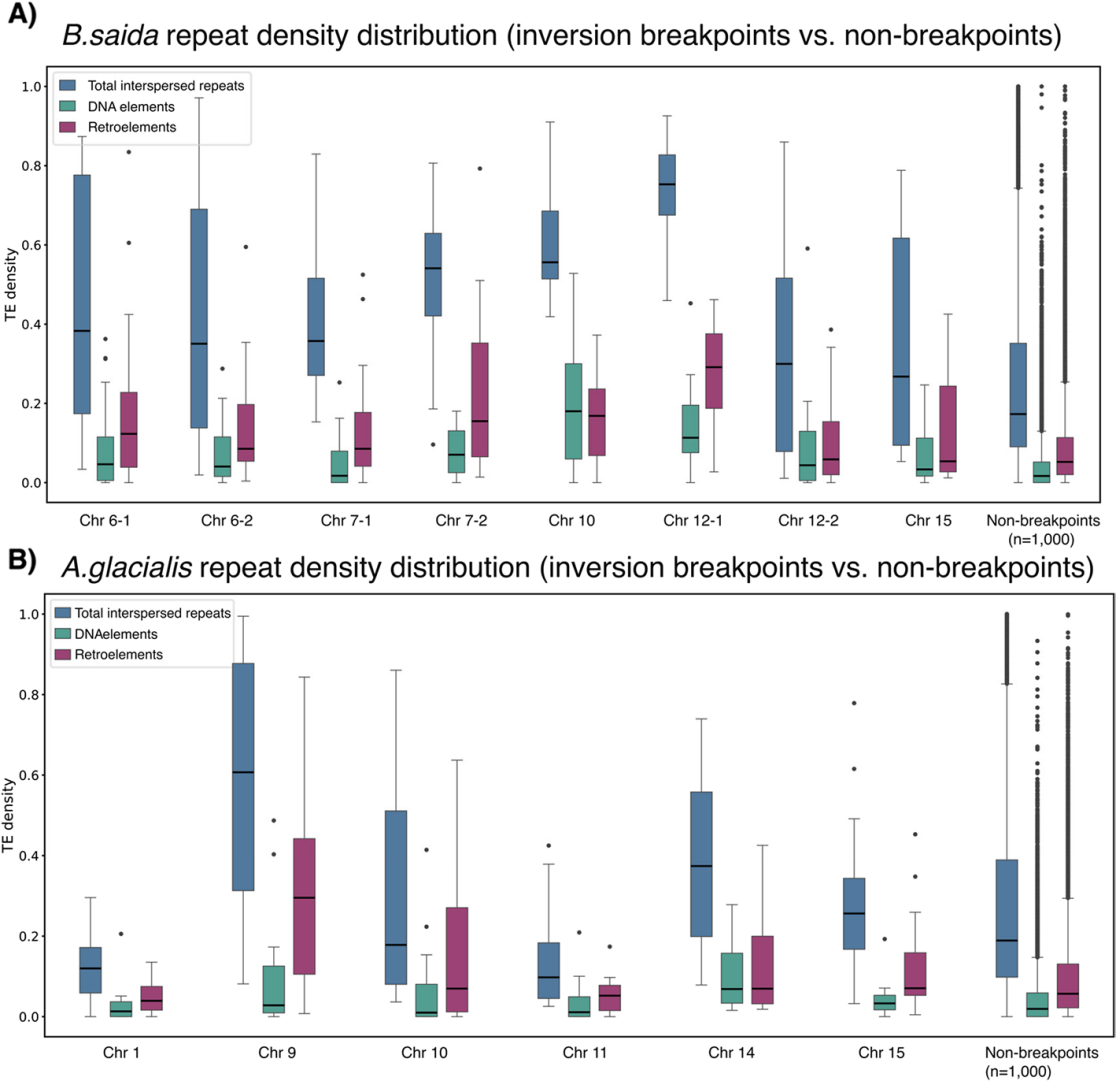
Repeat density distribution in inversion breakpoints vs. non-breakpoints. Distribution of TE densities (0–1) in polar cod for all TEs (blue), DNA elements (green), and retroelements (purple) for inversion breakpoint pairs of overlapping inversions in (**A**) polar cod and (**B**) Arctic cod. For each species, the distribution of repeat densities for 1,000 randomly selected non-breakpoint region pairs (see "Methods") are plotted in the far-right end of the plot

Given that repetitive DNA is frequently linked to chromosomal instability and facilitates novel structural variants, we examined whether repeats are associated with the inversion breakpoints of the overlapping inversions in the two Arctic codfish species. Estimation of repeat densities along each chromosome within 10 kb sliding windows revealed elevated TE densities within the majority of inversion breakpoints in polar cod when compared to randomly selected non-breakpoint region pairs from their respective genomes (Fig. 6; see "Methods"). For instance, the breakpoint regions of the inversions detected on Bs7, Bs10, and Bs12 showed significantly higher densities of total interspersed repeats (both DNA elements and retroelements) relative to the non-breakpoint region pairs in the polar cod (see Fig. 6A; Additional file 2, Figs. S14 and S15). For Arctic cod, we detected tendencies of accumulation of total interspersed repeats in several of the breakpoint regions (Fig. 6B; Additional file 2, Figs. S14 and S16). However, it was only for the inversion breakpoints on Ag9 that we detected significantly elevated levels of interspersed repeats and specifically retroelements (see Fig. 6B; Additional file 2, Figs. S14 and S16).

This overall pattern was consistent with the density peaks observed when plotting interspersed repeats across each chromosome (within 10 kb windows) and visually inspecting the breakpoint regions (Additional file 2, Figs. S15 and S16). Most polar cod breakpoints exhibit density peaks of interspersed repeats exceeding the 95th percentile of their respective chromosomal densities (Additional file 2, Fig. S15A), whereas the Arctic cod breakpoints show less pronounced enrichment (Additional file 2, Fig. S16A). Instead, several Arctic cod breakpoints display peaks of simple tandem repeats within the same breakpoint regions (Additional file 2, Fig. S16B).

Additionally, it should be mentioned that we detect for almost all of the chromosomes (in both species) a larger region with accumulations of interspersed repeats, and especially within the central regions of the fused chromosomes (see Additional file 2, Figs. S15 and S16). These accumulations of interspersed repeats are most likely associated with the centromere regions, which are known to harbor a high number of tandem repeats, including specific TE families [75].

### Antifreeze glycoprotein gene clusters in the Arctic codfishes, and their origin in the Gadidae

**Fig. 7:**
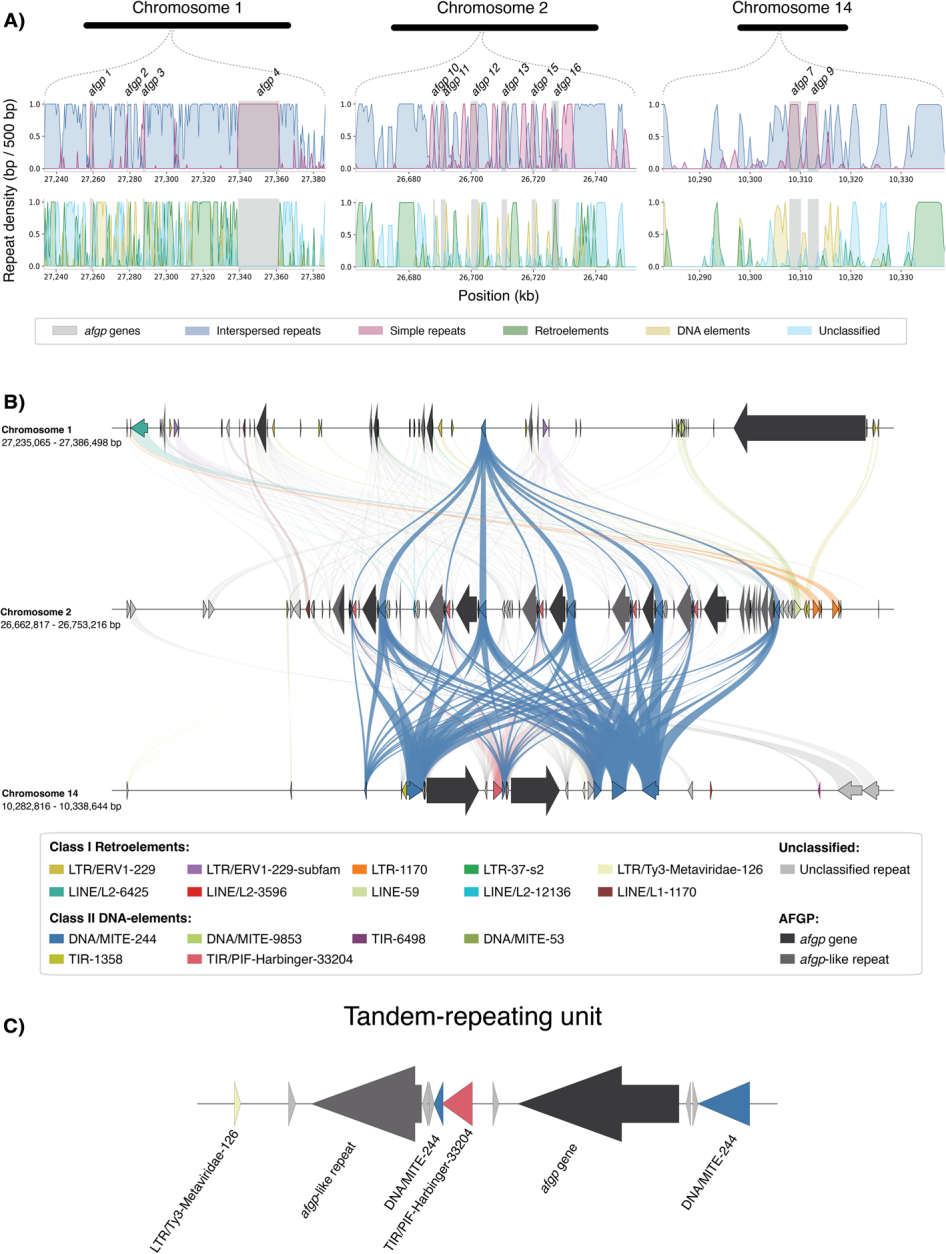
Molecular mechanisms of *afgp*-expansion in polar cod. **A** Repeat density within non-overlapping sliding windows of 500 bp across *afgp* gene clusters (± flanking regions of 25,000 bp) on chromosomes 1, 2, and 14 in polar cod. Positions are shown in kb (x-axis), and repeats are colored according to the legend. *afgp* genes are highlighted in grey. **B** Putative syntenic TE insertions in *afgp* clusters (± 25,000 bp flanking regions) on chromosomes 1 (top), 2 (middle), and 14 (bottom). Shared (syntenic) TE insertions are connected by filled, shaded lines. *afgp* genes are colored in black and *afgp*-like repeats in dark grey, and TE families are colored according to legend. **C** Tandem-repeating unit of *afgp* expansion located on chromosomes 2 and 14

The three *afgp* gene clusters, i.e., *afgp* cluster I, II, and III, previously described in polar cod [76, 77], were characterized in terms of localization, gene content, and organization in the present study. One of the main findings was the separate localization of the three clusters on three different chromosomes (Additional file 2, Fig. S17A–C), which have in earlier reports been identified to co-localize on one chromosome [77]. More specifically, the *afgp* gene cluster I was localized on chromosome 1, in close proximity to the fusion region, spanning 27.25—27.36 Mb from the first to last *afgp* gene, and containing four *afgp* genes (Fig. 7A; Additional file 2, Fig. S17A). Cluster II was identified on chromosome 14, within the intraspecies inversion in polar cod, containing two *afgp* genes spanning 10.30—10.31 Mb (Fig. 7A; Additional file 2, Fig. S17B), while cluster III was located on chromosome 2, located centrally, close to the fusion region, containing six genes spanning 26.68—26.72 Mb (Fig. 7A; Additional file 2, Fig. S17C).

The Maximum likelihood (ML) tree of the polar cod *afgp* genes suggests that the genes mainly branch into two groups with a bootstrap support of 84, where genes from cluster I group together, and genes from cluster II group together with cluster III (see Additional file 2, Fig. S18). Further, the *afgp* genes from cluster II branch out as a subgroup, however, with low branch support. The results demonstrate a likely relationship between the genes, where they group according to their respective gene clusters, suggesting a closer sequence similarity among genes within each cluster, compared to between clusters (Additional file 2, Fig. S18). Moreover, genes in cluster I exhibited the longest within-cluster branch lengths, with *afgp 1* showing the greatest sequence divergence towards the other genes (Additional file 2, Fig. S18).

The characterization of repeat densities across the different *afgp* gene clusters revealed high densities of simple repeats overlapping each annotated *afgp* gene (Fig. 7A, top panel), which is expected, as *afgp* genes have a simple repeat component [46, 77]. Moreover, we identified density peaks of TEs flanking each *afgp* gene (Fig. 7A, bottom panel). In particular, we observed elevated densities of DNA elements flanking the *afgp* genes on chromosomes 2 and 14 (Fig. 7A, bottom panel).

A detailed look at the TEs in the *afgp* gene clusters revealed strong similarities between cluster I (chromosome 1) and cluster III (chromosome 2) regarding the composition of TE families, as well as TE insertions flanking each gene (Fig. 7B). For the genes on cluster III, they appear to be flanked by identical repeats in a tandem repeated pattern (Fig. 7C). One of these repeat families was identified by manual curation as a MITE (DNA/MITE-224; see "Methods"), i.e., a Class II DNA transposable element with terminal inverted repeats (TIRs) where the internal transposase-encoding region has been partially or fully degraded. This TE family was named DNA/MITE-224, which there was one copy of in cluster I, but flanks every *afgp* gene in cluster II and III, suggesting it has been involved in the duplication of the *afgp* cluster on chromosome 2 and 14 by TE-mediated translocation.

Lastly, it should be mentioned that for Arctic cod, only one gene cluster was identified, i.e., which contained five genes spanning 61.42—61.59 Mb on chromosome 2 (Additional file 2, Fig. S17D). As a confirmation, inspection of the gene annotation revealed that the gene *mak16* was localized on chromosome 2 (61.61—61.62 Mb), a gene known to flank *afgp* gene cluster I in polar cod, Atlantic cod, and Atlantic tomcod (*Microgadus tomcod*) [76, 77]. The identification of one flanking gene (*mak16*) downstream of the identified gene cluster, and the fact that the end part of chromosome 2 in Arctic cod is syntenic with the genomic location of *afgp* cluster I in polar cod, suggest that the identified cluster corresponds to cluster I, which is the only cluster also found in Atlantic cod as well as Atlantic tomcod [77].

### Gene family expansion and contraction analyses

Gene family expansion/contraction analyses using CAFE5 found that European hake displayed the highest number of expanded gene families (*n* = 1096 gene families) while Atlantic cod (NEAC) displayed the lowest number (*n* = 318 gene families). For the contracted gene families, burbot showed the largest number of contracted families (*n* = 1087 gene families), while Atlantic cod (NCC) displayed the lowest number (*n* = 339 gene families). For more information on all species, see Additional file 2, Fig. S19. In this comparison, the Arctic codfishes were not particularly different in numbers of gene expansions or contractions, compared to the other codfishes.

GO-enrichment for expanded gene families for the two Arctic codfishes revealed significant enrichment for DNA integration, and purine ribonucleotide binding in polar cod (Additional file 1, Table S8), however, no significant GO-enrichment was detected for the contracted genes. In Arctic cod, a significant enrichment for retinoic acid receptor signaling pathway and ubiquitin-protein transferase activity was found for the expanded gene families, while enrichment for multiple functions coupled to cell signaling and cell communication was observed for the contracted gene families (Additional file 1, Table S8). Taken together, these analyses did not reveal any strong link to the genomic rearrangements observed within the Arctic codfishes investigated in this study.

### Elucidating the relationship of the Arctic codfishes

A ML phylogenetic tree based on the concatenated protein-coding genes from the mitochondrial genomes of an extended number of species (see "Methods" for full list), placed Arctic cod as sister taxon to the Atlantic cod (*Gadus morhua*), although with low support, Shimodaira-Hasegawa-like approximate likelihood ratio (SH-alrt) of 26.6 and ultrafast bootstrap approximation (UFBoot [78]) of 56 (Fig. 1). Pairwise identity analysis between *Melanogrammus aeglefinus*, *Arctogadus glacialis, Boreogadus saida,* and *Gadus morhua* for each mitochondrial protein-coding genes (PCGs) revealed that the PCGs between *Arctogadus glacialis, Boreogadus saida,* and *Gadus morhua* had high pairwise similarity (Additional file 2, Fig. S20), suggesting that the PCGs might not provide sufficient information for resolving the branch represented by *Arctogadus glacialis*.

The reduced ML and Bayesian phylogenies inferred using only the mitogenomes of the species sequenced in the present study (except for Atlantic haddock, see Additional file 5 for details) and using concatenated PCGs resulted in the same topology as the extended mitochondrial phylogeny (Fig. 1; Additional file 2, Figs. S21 and S22). Moreover, the ML and Bayesian tree inferences based on the complete mitogenomes differed in the placement of Arctic cod, where the ML analysis placed Arctic cod and polar cod as sister species with a bootstrap support of 62, whereas the Bayesian phylogeny placed Arctic cod as sister taxon to the Atlantic cod, with a posterior probability of ~ 0.76 (Additional file 2, Figs. S23 and S24).

Fitting a phylogenetically informed linear regression by applying the mitochondrial ML tree representing 13 codfish species (implemented in the R package phylolm, see "Methods" for details) revealed a significant association between reduced chromosome number and cold-water preference, with a p-value of 0.006 and 0.026 after correcting for multiple testing. Tests for chromosome number vs. northerly geographical distribution were also significant, with a p-value of 0.003 and 0.011 after correcting for multiple testing (see "Methods"; Additional file 1, Table S9; Additional file 2, Fig. S25 for more details on key model parameters).

The BUSCO search for phylogenetic analyses uncovered between 88.4% and 93.9% complete and single-copy BUSCO genes for the genome assemblies presented in this study, including the already published NEAC genome assembly (gadMor3.0) [62] (Additional file 1, Table S10). In total, 2494 BUSCO genes were found to be shared among all species. 555 BUSCO genes were removed after quality filtering (see "Methods"; Additional file 2, Fig. S26 for details).

**Fig. 8:**
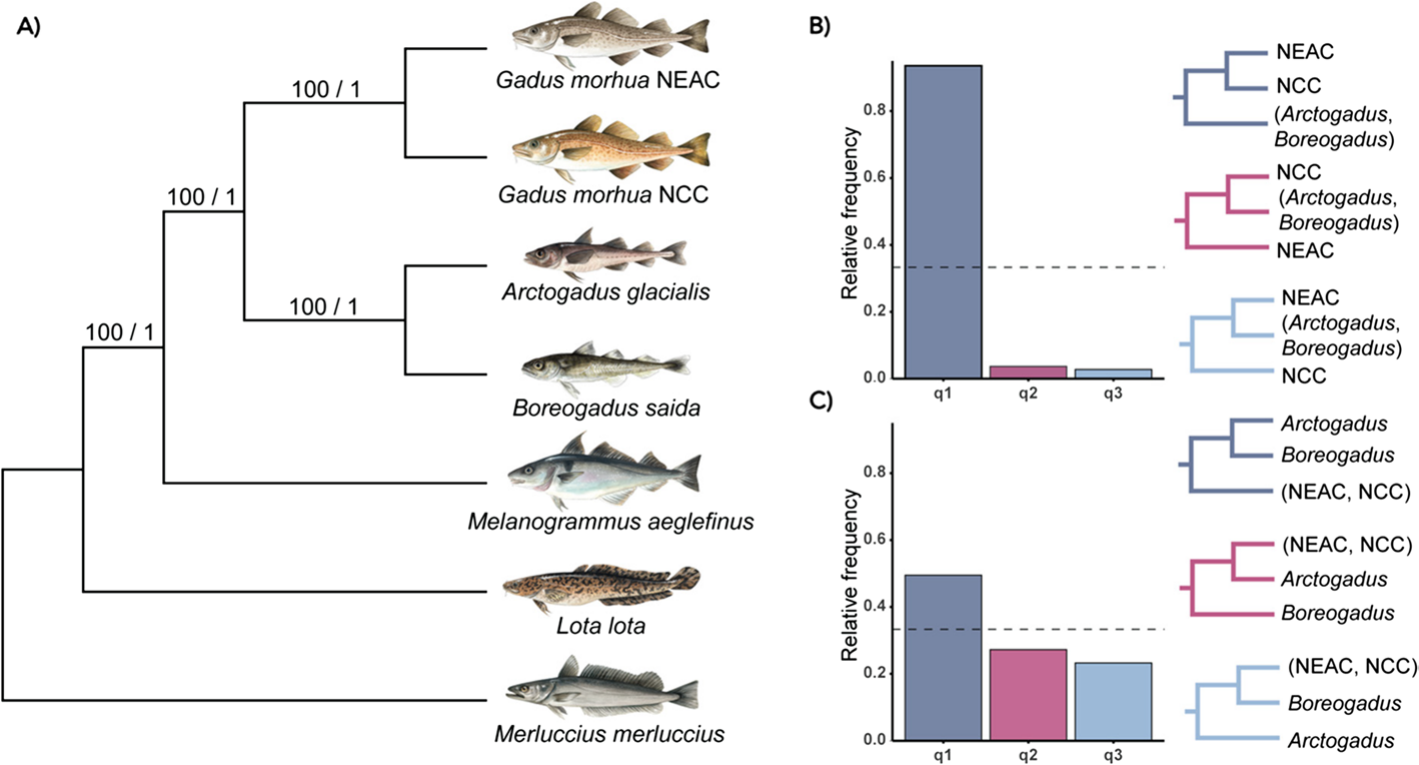
Multigene phylogenies of the Gadiformes species.** A** Multispecies coalescent species tree produced with Astral-III [65] of 1939 BUSCO gene trees shared between all taxa. Branch support is given as multi-locus bootstrap/local posterior probability (see "Methods"), and branch lengths are given as coalescent units. **B** Quartet frequencies calculated using DiscoVista [79] with European hake (*Merluccius merluccius*), burbot (*Lota lota*), and Atlantic haddock (*Melanogrammus aeglefinus*) specified as outgroups.** B** Quartet frequencies of the internal branch separating the two Atlantic cod (*Gadus morhua*) ecotypes, i.e., NEAC and NCC, from the other species, and (**C**) quartet frequencies of the internal branch separating Arctic cod (*Arctogadus glacialis*) and polar cod (*Boreogadus saida*) from the rest of the species. Each tree depicted for (**B**) and (**C**) represents alternative hypotheses for the unrooted quartets shown in the bar plot, outgroup not shown

Multispecies coalescent (MSC) analysis using Astral-III of the 1939 BUSCO gene trees resulted in a species tree with good bootstrap and posterior probabilities (Fig. 8A; Additional file 2, Fig. S27). In the resulting tree, Arctic cod and polar cod were placed as sister species, and the Atlantic cod was placed as the sister taxon to this group (Fig. 8A). The ML phylogeny of the concatenated BUSCO genes with an alignment length of 3,364,610 bp resulted in a tree with the same topology as the species tree (Additional file 2, Fig. S28). Quartet frequencies revealed that the majority of gene trees agree on the placement of NEAC and NCC as expected (Fig. 8B; Additional file 2, Fig. S29). However, the relationship of Arctic and polar cod in relation to *Gadus morhua* shows some level of discordance (Fig. 8C; Additional file 2, Fig. S29) with the highest frequency of gene trees placing Arctic cod (*Arctogadus glacialis)* and polar cod (*Boreogadus saida*) as sister species (0.495), Arctic cod (*Arctogadus glacialis)* as the sister lineage to *Gadus morhua* (0.272), and lastly polar cod (*Boreogadus saida*) as the sister lineage to *Gadus morhua* (0.233). Due to the discordance, we further investigated the positioning of the BUSCO genes in the genomes, which could potentially contribute to uncertainties in the phylogenetic placement of the species. We found that 148 and 314 of a total of 1939 BUSCOs were localized within the species-specific inversions in Arctic cod and polar cod, respectively.

## Discussion

In this study, we present chromosome-level reference genomes for six codfish species that were used in a comparative setting to investigate the evolutionary genomic changes that have taken place within this lineage. From these in-depth analyses, we uncovered independent lineage-specific chromosomal fusions and inversions, as well as translocations — especially within the cold-water adapted species — that are seemingly modulated by the activation of TEs. Moreover, our findings demonstrate that such massive genomic modifications can take place across relatively short evolutionary time scales (within ~ 4 million years [10]) and are key features that have likely played an important role in divergence processes and adaptation to freezing environmental conditions.

### Chromosome number variability in codfishes

The sequencing and assemblage of reference genome assemblies for six codfish species (the non-migratory ecotype of Atlantic cod (NCC), polar cod, Arctic cod, Atlantic haddock, burbot, and European hake) resulted in assemblies of high contiguity and BUSCO completeness metrics. For the two Arctic codfish species, the genome assemblies (including the draft Oxford Nanopore genome assemblies) uncovered species-specific chromosomal fusions, i.e., five in polar cod vs. eight in Arctic cod, which have given rise to a reduced chromosome number in both species compared to their closely related codfish species, where the chromosome number ranges between *n* = 22–24 (see Additional file 1, Table S1). This rather striking result demonstrates that none of the fusion events seemed to be of common origin, as none of the fused chromosomes were homologous between the species, thus indicating that they have taken place during relatively short evolutionary time scales (i.e., within ~ 4 million years [10]). Moreover, a reduced set of chromosomes has also been reported in two codfish species residing in Arctic and sub-arctic waters [44, 80, 81], i.e., the Navaga (*Eleginus nawaga*) and Saffron cod (*Eleginus gracilis*) (Fig. 1; Additional file 1, Table S1), which are in line with our finding that the reduced chromosome number is significantly associated with northward distribution and/or preferred sea temperature, i.e., Arctic conditions. Similarly, a pronounced reduction in number of chromosomes is documented for some of the species within the suborder of the cold-water adapted Antarctic notothenioids [40–42, 82]. These observations could be examples of convergent/parallel evolution, and thus, indicate a likely adaptive significance associated with a reduced number of chromosomes and/or associated with the fusion process of chromosomes, i.e., coupling novel genomic regions together, especially for species inhabiting high-latitude oceans.

The evolutionary importance of the chromosomal fusions is also indicated in two population sequencing studies of polar cod and Arctic cod, which both demonstrate that chromosomal fusions display unique genomic characteristics, including signals of elevated LD, positive estimates of Tajima's D, and reduced D_XY_ estimates compared to surrounding regions [53, 54]. Signals of higher LD indicate lower recombination rates within these central regions of the chromosomes, vs. lower LD signals, and thus, higher recombination rates are detected in the distal regions of the chromosomes. These findings are in line with previous studies reporting that chromosome size (due to fusions) and localization of the new centromere impact the recombination landscape across chromosomes [83–85]. Reduced recombination across a fused region of a chromosome is likely to have evolutionary consequences, i.e., by increasing the overall linkage between genes and genetic elements residing within this region, leading to genes being inherited together more frequently. Chromosomal fusions can also bring together previously distantly located genes and genetic elements that may be of particular advantage in a given environment [86]. Therefore, rearrangements of chromosomes may give rise to new and advantageous combinations of genes/genetic elements, as well as increase the likelihood of the new combinations being inherited together. Here we could highlight that two of the three *afgp* gene clusters in polar cod (cluster I and cluster II) were localized in close vicinity of the chromosomal fusions on chromosome 1 and chromosome 2, while cluster II was located within the chromosomal inversion detected on chromosome 14 (i.e., Bs14). In other words, all three *afgp* gene clusters are located within regions that display high LD patterns, potentially linking beneficial allele combinations of the *afgp* genes and/or regulatory elements. Furthermore, two of the fused regions in polar cod harbor larger inversions, as well as in one of the fused chromosomes in Arctic cod, i.e., on Bs2, Bs3, and Ag7. The fused region on Bs2 — as mentioned above — is associated with one of the *afgp* gene clusters. It should also be noted that the fused chromosome Bs5 has been identified to harbor a putative sex-determining region in polar cod [53], supporting that these fusions harbor genes of evolutionary importance.

Another direct beneficial role of chromosomal fusions is the reduced risk for segregation errors, as seen with higher chromosome numbers [85]. In this regard, using the Hi-C combined contact map with the repeat annotation, we were able to localize centromeres for most of the chromosomes, and especially within the larger fused chromosomes. Intriguingly, the centromeric regions of the fused chromosomes were by large, identified at the center of the fused chromosomes and displayed rather variable size. Thus, the species-specific chromosomal fusions detected for the partially sympatric sub-Arctic and Arctic gadids [44] could potentially act as barriers to gene flow, and thus, be linked to the diversification and speciation processes within the lineage (see speciation by chromosomal rearrangements discussed in [86–90]).

### Chromosomal inversions in the Arctic gadids

The in-depth investigation of the genomic localization of the previously reported intraspecific chromosomal inversions found in Atlantic cod, polar cod, and Arctic cod uncovered multiple partly overlapping inversions among the species, as well as for some of the breakpoint regions (Fig. 2B for overview, and Fig. 4A-D for detailed examples). None of these inversions were 100% overlapping, i.e., indicating that the chromosomal inversions are species-specific, and thus of evolutionary independent origin. However, given the fact that local genomic reorganizations seem to have happened regularly within the different species, e.g., such as inter- and or intra-chromosomal translocations, a common origin for some of the inversions cannot be ruled out completely. For instance, the rather large inversion detected when comparing the genome assemblies of the non-migratory and migratory Atlantic cod and the corresponding inversion detected in polar cod on chromosome 10 (Ne10 vs. Bs10) could potentially have a shared ancestral origin, followed by successive translocations within polar cod (Fig. 5B). Similarly, a common origin cannot be excluded for the partly overlapping inversion found between polar cod and Arctic cod on Bs6 vs. Ag9, as well as for the inversions found to be overlapping to some extent between Arctic cod and Atlantic cod on Ag11 vs. Ne7. For the latter, the discrepancy is largely due to the translocation of the breakpoint regions within Arctic cod.

For the inversions found to partly overlap between polar cod or Arctic cod vs. Atlantic cod (on Ne10 and Ne7), limited sequence similarities (F_ST_ and D_XY_) were detected. This is likely due to polar cod and Arctic cod not sharing overlapping inversions in the specific regions investigated. For the remaining partly overlapping inversions, we uncovered smaller and/or larger local regions with high sequence similarity, i.e., some covering almost the entire inversion region, whereas others include smaller regions often located at the breakpoint regions of the inversion. Such signatures of conservation could imply that these genomic regions harbor genes and/or regulatory elements of evolutionary importance that are either under i) purifying or parallel selection [91, 92], and/or linked to ii) past gene conversion events [10]. For instance, when spanning larger areas, these regions could be selected for as supergenes encompassing locally adapted genes/alleles. If the identified regions, however, are located in the breakpoint of an inversion, this could suggest that these regions harbor fewer but still important genes that are linked together via the formation of an inversion [93]. Since polar cod and Arctic cod have overlapping geographical distributions [94, 95], it is plausible to assume that they encounter comparable environmental conditions, and thus, likely similar selection pressures acting on the same genomic regions. In this regard, we could highlight the conserved genomic regions detected within the overlapping chromosomal inversion on Ne12, Ag10, and Bs7, which is also found to be partly syntenic with an overlapping inversion detected between the two darter sister species (*Etheostoma perlongum* and *Etheostoma maculaticeps*) as well as in rainbow trout (*Oncorhynchus mykiss).* These findings combined further strengthen the idea of these genomic regions being of high evolutionary impact [96].

Moreover, the accumulation of specific TE classes as well as short tandem repeats in the breakpoint regions could also putatively be linked to the degree of conservation detected between the two Arctic gadids within these regions, and thus, underscore the potential evolutionary impact of such interspersed elements in the formation of the inversions in these species, as reported in other study systems [19, 21, 23, 97–99]. In general, codfishes are known to harbor large amounts of repeated sequences, in particular short tandem repeats [100–103] as well as transposable elements [103]. Recently, the involvement of TEs, and more specifically MITEs belonging to a family of *hAT* transposons, in the formation of the well-known inversions in Atlantic cod was documented [104]. From this, and the fact that the overlapping regions were often associated with translocations, it is likely that the shared homologous regions underlying chromosomal inversions contain such interspersed repeats, and that they are involved in the mechanistic formation of the inversions detected within the Gadiformes lineage.

Manual investigation of the localization of the previously described hemoglobin gene clusters, the LA- and MN-clusters [105], revealed that both were located in association with the partly overlapping species-specific inversions in the Arctic gadids, and give further support for the adaptive significance of the genomic reorganizations within codfishes. The hemoglobin genes have been linked to temperature adaptation through polymorphisms and copy-number variation in codfishes [106–109]. Interestingly, the LA-cluster was localized at the breakpoint region of the inversion on Ag1 in Arctic cod, while the cluster was found inside the inversion in polar cod (Bs15). One hypothesis for the maintenance of polymorphic chromosomal inversions after the inversion occurs is that inversions can rise in frequency within populations due to beneficial mutations close to or at inversion breakpoints [93]. Moreover, genes proximal to inversion breakpoints may exhibit differential expression [110], and genes located at breakpoints can contain different exon sequences in the alternative inversion haplotypes [111]. Based on this, we can hypothesize that hemoglobin and co-regulated genes (such as the *fbxl5* gene) could be affected by the inversion(s) on Ag1 and Bs15, but to what extent remains to be explored.

The second hemoglobin cluster, the MN-cluster, was found to be localized inside the inversion on Ag15 in Arctic cod, while on the boundary/breakpoint of one of the two inversions located on Bs12 in polar cod. These two chromosomes are homologous to Ne2 of Atlantic cod, where an inversion has also been detected [55, 57, 66], but even if the MN-cluster is localized outside of the inversion, there is seemingly a link between this inversion and the hemoglobin genes also in Atlantic cod [112]. Moreover, the inversion on Ne2 in Atlantic cod has been linked to adaptation to environmental differences in oxygen, salinity, and temperature between Atlantic cod populations in the North Sea and the Baltic Sea [71, 113]. Taken together, the MN-cluster was found to be in close association with species-specific inversions in all three species, which suggests that these homologous regions are likely of evolutionary importance in the different species. However, further investigation is needed to fully resolve the evolutionary significance of these inversions and how they relate to hemoglobin functionality.

### Origin of antifreeze glycoprotein gene clusters in polar cod

The localization and characterization of the known *afgp* gene clusters in polar cod revealed that the three gene clusters are located on three different chromosomes, in contrast to being co-localized on a single chromosome as previously reported [77]. For Arctic cod, only one *afgp* cluster was identified, i.e., homologous to cluster I, which is the only cluster found in Atlantic cod as well as Atlantic tomcod [77], and thus, can be regarded as the ancestral *afgp* gene cluster within the codfishes, which is further supported by our phylogenetic analysis.

In polar cod, the ancestral *afgp* cluster on chromosome 1 (cluster I) and the cluster on chromosome 2 (cluster III) were found to share multiple TEs belonging to the same families (e.g., DNA/MITE-244, LINE-59, and LTR-1170). These similarities suggest that the *afgp* gene(s) in cluster II could have originated from an ectopic recombination event, which translocated the *afgp* gene(s) from chromosome 1 to chromosome 2. Such events can occur through the mispairing of stretches of similar sequences between chromosomes (i.e., the TE insertions), leading to non-allelic homologous recombination (NAHR) [75, 114, 115]. Subsequently, the *afgp* genes in cluster III appear to have expanded by tandem duplications likely driven by similar TEs, supported by the observed repeating unit of *afgp* genes flanked by identical TE families in all of the *afgp* genes on chromosome 2 (see Fig. 7C). One of these TE families was identified as a MITE (DNA/MITE-244) with intact TIRs. The involvement of DNA transposons with TIRs, particularly MITEs, in driving tandem gene duplications has been proposed for toxin gene expansions in the green-head ant [116]. Given that the *afgp* genes in cluster III are flanked by the same MITE (DNA/MITE-244), it is plausible that further activation of this TE facilitated a translocation event giving rise to the *afgp* cluster on chromosome 14. Similar to certain DNA elements capable of gene capture and co-translocation [117], the TEs flanking the *afgp* genes on chromosome 2 could allow for *afgp* to hitchhike with the TEs to the new genomic location on chromosome 14 [22, 75]. This hypothesized origin of polar cod *afgp* cluster II and III was further supported by our phylogenetic analyses. Here, it could be noted that the genomic region that harbors the *afgp* gene cluster III in polar cod is homologous to a region on chromosome 16 in Atlantic cod, where *afgp*-like sequences have been identified [46], Thus, it is possible that the NAHR event took place prior to the splitting of polar cod, Arctic cod, and Atlantic cod, but that the translocated *afgp* genes became pseudogenized in the two latter lineages.

Taken together, our results suggest that the origin of cluster II and III was facilitated by TE activity, and that the translocation event resulting in *afgp* cluster II, is a lineage-specific novelty in polar cod. Thus, TEs have likely facilitated the expansion of the *afgp* gene repertoire in this species, a gene family crucial for this species ability to inhabit the freezing waters of the Arctic Ocean regions.

### Are phylogenetic investigations hampered by the chromosomal rearrangements?

The phylogenies constructed using BUSCO, as well as mitochondrial genes extracted from the newly generated genome assemblies, uncovered some uncertainty in the placement of the Arctic gadids. Depending on the markers and methodology used, Arctic cod was either placed as sister taxon to *Gadus*, or as sister species to polar cod, consistent with incongruences earlier reported [10, 118–120]. At the mitochondrial level, our results show that the Arctic cod is a sister taxon to *Gadus*, but with low bootstrap support. The low support likely reflects insufficient phylogenetic signals in mitochondrial PCGs, which could be due to purifying selection (shown by Wilson et al. [120]), as the PCGs showed high sequence similarity across species (Additional file 2, Fig. S20). Conversely, in the MSC BUSCO phylogeny, Arctic cod is placed as a sister species to polar cod. The quartet frequency analysis for the BUSCO phylogeny demonstrated discordance among the gene trees in the placement of Arctic cod and polar cod as sister lineage *Gadus* (Fig. 8C). The uncertainty in the placement of Arctic cod in the BUSCO phylogeny could be due to several factors affecting phylogenetic inference, e.g., introgressive hybridization [10, 74], incomplete lineage sorting, and/or large amounts of highly linked genomic regions leading to an altered recombination landscape [121, 122]. For the latter, quantification of BUSCOs within and outside identified chromosomal inversions in polar cod and Arctic cod revealed some BUSCOs to be localized within these regions, possibly contributing to uncertainties in the phylogenetic placement of the species.

## Conclusions

In this undertaking, we uncover that the cold-water adapted polar cod and Arctic cod have undergone rapid and extensive genomic rearrangements, which include both chromosomal fusions as well as inversions, presumably modulated by TE activation. A mediating role of TEs was also documented for the formation of the three known *afgp* gene clusters in polar cod, where two of them were found to localize within the fused regions on chromosome 1 and chromosome 2, while the third was found within the chromosomal inversion on chromosome 14. Combined, our findings suggest that these genomic modulations are key features in Arctic codfish species, and thus, could potentially have played important roles in the speciation processes within the lineage, and/or adaptation to freezing environmental conditions.

Finally, our attempt to reconstruct the phylogenetic relationship between the codfishes resulted in conflicting topologies for the BUSCO and mitochondrial phylogenies. We speculate that incongruences in the placement of Arctic cod might, at least partly, stem from the massive genomic reorganizations that have taken place, which cover large proportions of the genomes, encompassing a combination of shared and unique evolutionary trajectories.

## Methods

### Sample collection

We whole-genome sequenced a total of six codfish species in the present study. These specimens, along with their sampling locations, are as follows: Arctic cod (*Arctogadus glacialis*) from Tyrolerfjorden, Northeast Greenland; polar cod (*Boreogadus saida*) from Tyrolerfjorden, Northeast Greenland; non-migratory Atlantic cod (*Gadus morhua*) i.e. NCC from Lofoten area, Norway; Atlantic haddock *(Melanogrammus aeglefinus)* from Tromsø area, Norway; burbot (*Lota lota*) from Lake Stuorajavri, Finnmark, Norway; and European hake (*Merluccius merluccius*) from Oslofjorden, Norway. For a detailed overview of the specimens, as well as the tissue used for each sequencing technology, see Additional file 1, Table S11, and Additional file 2, Fig. S30.

All samples used in this study were collected in a responsible manner in connection with different research surveys. The fish were humanely sacrificed before sampling in accordance with the guidelines set by national and international animal welfare laws (e.g., https://norecopa.no), and thus no specific legislation was needed.

### Generation of chromosome-anchored genome assemblies of six codfish species

To construct high-quality chromosome-length genome assemblies, we leveraged a combination of PacBio long-read sequencing and chromosome conformation Hi-C reads to scaffold contigs (see Additional file 5 for a detailed description of sequencing and assemblage). For some of the species, we in addition utilized 10X linked reads to link contigs, and Illumina sequencing data for polishing, to ensure high base quality. In brief, first, primary assemblies were constructed from PacBio long reads using Flye [123]. Next, for Arctic cod, polar cod, and Atlantic cod (NCC), to assemble primary contigs into chromosome-length scaffolds, contigs were first scaffolded using 10X linked reads and the program Scaff10X [124]. For all species assemblies, Hi-C linked reads were mapped and processed using either 3D-DNA [125] or YaHS [126] (Atlantic haddock). The 3D-DNA/YaHS draft assemblies were visualized and manually inspected in the Juicebox program suite [127] or PretextView [128] (Atlantic haddock). Manual splitting of scaffolds that were assembled, but that exhibited low levels of Hi-C contact points between them was performed. Lastly, for Arctic cod, polar cod, burbot, Atlantic haddock, and Atlantic cod (NCC), the assemblies were polished using both long and short-read data. For long-read polishing, pbmm2 [129] was used to map PacBio reads, and polishing was done using gcpp [129]. Lastly, Illumina paired-end reads were mapped by using minimap2 [130], and subsequent base calling and polishing were done using Freebayes [131]. Finalized assembly metrics were calculated using the assemblathon_stats script [132], and completeness was assessed by the Benchmarking Universal Single-Copy Orthologs (BUSCO) software [133]. Moreover, the full mitochondrial genomes of the six Gadiform species were assembled using either MitoVGP [134] or MitoHiFi [135], depending on the available input data (see Additional file 5).

To evaluate and validate the chromosomal architectures of the genome assemblies, we leveraged two newly generated draft assemblies for polar cod [136] (fBorSai1.1) and Arctic cod [136] (fArcGla1.1), respectively. These draft genomes are generated using a combination of ONT and Hi-C sequencing data from different individuals than the reference genomes. Macro-synteny of the PacBio and ONT draft assemblies for each species was compared and assessed using the D-genies interactive platform [137] and the software SyRI v1.5 [67]. The ONT draft assemblies resulted in 15 and 18 chromosomes for Arctic cod and polar cod, respectively, concordant with the PacBio assemblies (Additional file 2, Figs. S2 and S3). Further, synteny comparisons showed that the PacBio and ONT draft assemblies display highly similar intrachromosomal architecture (Additional file 2, Figs. S2 and S3).

The reference genome assemblies were annotated using either i) a combination of funannotate [138] with RNA sequencing data processed with HISAT v2.1.0 [139], Portcullis v1.2.0 [140], and Mikado v2.0rc6 [141], or ii) a combination of miniprot v0.5 [142], funannotate v1.8.13 [138], EvidenceModeler v1.1.1 [143], AGAT [144], and InterProScan v5.47–82 [145]. For more details, see Additional file 5.

For the purpose of ensuring a comparable baseline for gene family expansion and contraction analyses, we did an additional gene annotation where the same pipeline was applied to all six reference genomes. This consisted of an approach similar to step ii) above, but with some updates to input data. See Additional file 5 for more details.

### Repeat annotation in the Arctic codfishes

To annotate the repetitive DNA in the Arctic codfishes, we used an existing manually curated TE library generated for the closely related Atlantic cod (NEAC; [104]) to perform an initial masking of the Arctic (PacBio HiFi) and polar cod (PacBio HiFi and ONT) assemblies using RepeatMasker v4.1.5 [146] (Additional file 1, Table S12; Additional file 2, Fig. S31). To obtain better resolution of TEs surrounding particular genes of interest (see *Identification of antifreeze glycoprotein genes, and their genomic location in the Arctic codfishes*), we manually curated a subset of TEs that were prioritized according to number of insertions and proximity to the *afgp* gene clusters in polar cod. This subset of TE consensus sequences was manually investigated using structural and homology-based evidence from MCHelper [147], TE-Aid [148], and PASTEC [149]. Incomplete sequences were examined using a BLAST-extension-alignment protocol to delineate their true boundaries, and the output consensus sequences were searched against the Dfam [150] and Censor [151] online databases for reclassification. We then added the manually curated consensus sequences to the initial NEAC-specific TE library, resulting in a semi-curated library for Arctic codfishes. Finally, this TE library was used to mask the Arctic and polar cod genome assemblies using RepeatMasker v4.1.5, using the slow search setting to increase sensitivity.

### Genome-wide synteny analyses between codfish genomes

Based on previous studies, the suggested ancestral number of chromosomes in teleosts has been estimated to be *n* = 24–26 [50–52]. Based on this, we conducted a first screening to evaluate the chromosome configuration of Atlantic cod (*n* = 23) by dot plotting the NEAC genome assembly [62] (gadMor3.0, NCBI refseq assembly: GCF_902167405.1) against platyfish (*n* = 24) (*Xiphophorus maculatus,* GCF_002775205.1_X_maculatus-5.0, NCBI refseq assembly: GCF_002775205.1) as well as John Dory (*n* = 22) (*Zeus faber,* GCA_960531495.1_fZeuFab8.1, NCBI refseq assembly: GCA_960531495.1) using minimap2 v2.24 [130] and the D-genies interactive platform [137]. This initial screening confirmed that the chromosome configuration in Atlantic cod is similar to the outgroup species, with only a few putative fusions, encompassing two of the Atlantic cod chromosomes (for more details, see Additional file 4).

Intraspecies chromosomal synteny between the Atlantic cod ecotypes (NCC and NEAC) was investigated by first obtaining an alignment file between the genome assemblies using Minimap2 v2.17 [130] with settings -ax asm5 –eqx, then chromosomal rearrangements were identified and visualized using the software SyRI v1.5 [67].

Subsequently, by a gene-order based approach using the program MCScanX [63] and visualization using the SynVisio [64] online platform, chromosomal synteny between the genome assemblies of the six codfishes was performed. Here, genome collinearity, chromosomal homologies, and chromosomal rearrangements were manually inspected and identified in a pairwise manner with Atlantic cod, polar cod, and Arctic cod as references, and the five other species as queries.

The MCScanX analysis was performed according to developers’ recommendations, first doing reciprocal all by all BLAST [152] search between all proteins of the annotated gene for all six species, using BLAST + v2.11.0 [152] with the settings: blastp -e 1e-10 -b 5 -v 5 -m 8. The hits were afterward anchored to a bed-like file made from the annotation GFF file, with information about gene names and the genomic locations of each gene. Synteny blocks were then built using MCScanX with default settings, i.e., gene gap size set to 10. Results were visualized on the SynVisio interactive webpage as syntenic maps between the genome assemblies.

### Genefamily expansion and contraction analyses

OrtoFinder [153] was run on the additional gene annotations to group the genes into orthogroups. CAFE5 [154] was applied to gene families and species tree as output by OrthoFinder to model gene gain and loss across the species tree. This resulted in a set of gene families that have significantly expanded at various points in the phylogeny.

GO-enrichment of expanded and contracted gene families in the Arctic codfishes was performed following the steps described in https://github.com/ebp-nor/workshop-2024/blob/main/day3_comparative_genomics/GO-enrichment.md (accessed 08.09.2025). In short, using the output from OrthoFinder and CAFE5, GO terms are extracted from GFF files, and combined per gene family, and significantly expanded and contracted gene families were exported to a new list for polar cod and Arctic cod using the Tidyverse package [155] in R [156]. Then GMT helper (https://biit.cs.ut.ee/gprofiler/gost, accessed 08.09.2025) was used to convert the annotation to a valid GMT file. Finally, GO-enrichment was performed on the g:Profiler web platform (https://biit.cs.ut.ee/gprofiler/gost, accessed 08.09.2025).

### Detection of overlapping chromosomal inversions

From previous literature and ongoing studies, several chromosomal inversions have been detected within Atlantic cod [55–58, 66], polar cod [53], and Arctic cod [54]. For Atlantic cod, four major inversions have been detected: on LG01, LG02, LG07, and LG12 [55–58, 66] (synonymous with Ne1, Ne2, Ne7, and Ne12 in the present study), where the inversion on Ne1 has been linked to differentiation between the migratory NEAC vs. the non-migratory NCC. In recent studies, a total of 20 inversions in polar cod [53] and 11 inversions in Arctic cod [54] have been reported and suggested linked to cryptic ecotypes and/or local adaptation.

Using this information and plotting the location for the different inversions by position within the genome assembly of each of the species, they were identified onto their respective chromosomes using the SynVisio [64] interactive homepage (as described above). For Atlantic cod, the breakpoint regions for the inversion were identified using SyRI results between the ecotypes (see above for details). We then inferred whether some of these inversions were overlapping or not between these three species (see Fig. 2B). For visualization as well as simplicity, the chromosome numbering of Atlantic cod is renamed from linkage groups LG01-LG23 to chromosome numbers Ne1-Ne23 in this paper (see Figs. 2, 3, 4 and 5).

To investigate potential associations between the identified genomic rearrangements, i.e., inversions and the chromosomal fusions, and repetitive DNA, we plotted the repeat density (interspersed repeats and simple repeats) within non-overlapping sliding windows of 10,000 bp along all of the chromosomes in both of the Arctic codfish species. These plots were used to visually evaluate the repeat density peaks within the overlapping inversions, focusing on the breakpoint regions (for coordinates see Additional file 1, Tables S6 and S7; ± 50,000 bp). These estimates were compared to the respective chromosomal medians and 95th percentiles. Furthermore, we plotted the distribution of repeat densities (all TEs, DNA elements, retroelements) within the breakpoints of the overlapping inversions (i.e., five inversions in Arctic cod and seven in polar cod, see Additional file 1, Table S5). We then compared the inversion breakpoint distributions to randomly selected pairs of 100 kb non-breakpoint regions using boxplots. This region interval was chosen to match the size of the inversion breakpoint intervals. Non-breakpoint region pairs were always drawn from the same chromosome and sampled 1,000 times from random genomic locations. To formally test whether inversion breakpoints were associated with elevated TE densities, we performed a permutation test in which we plotted the median densities of the non-breakpoint regions as a histogram and compared them to the median densities of the inversion breakpoint pairs. Median densities exceeding the 95th percentile of the non-breakpoint distribution were considered significant.

### Validation of chromosomal fusions and inversions using ONT genome assemblies

Validation of the chromosomal rearrangements was obtained by leveraging the ONT draft genome assemblies for polar cod and Arctic cod. Importantly, these assemblies were generated from different individuals than the PacBio genome assemblies and can therefore provide additional confirmation that the fusions and inversions are true rearrangements. For both species, Minimap2 v2.26 was used to map the ONT draft genome assembly towards the PacBio reference assembly, applying the settings -ax asm5 –eqx. Then, SyRI v1.5 was run as follows, here with polar cod as example: syri \ -c boreo_vs_hap1.sam \ -r boreogadus_saida_mt_09042020.Chr1-18.fa.gz \ -q fBorSai1.1.hap1.renamed.Chr1-18.fa.gz -F S \ –prefix syri_boreo_vs_hap1. The results were plotted using plotsr [157]. The VCF output from SyRI containing the called structural variants, was loaded for manual inspection in JBrowse 2 [158]. Furthermore, for Arctic cod we mapped the ONT reads against the PacBio reference genome and manually inspected how reads behaved around fusion, and inversion breakpoints. This was done by first identifying inversions between the two genome assemblies (PacBio and ONT draft) and thus the two individuals using SyRI v1.5 [67], to get detailed breakpoint location for the inversions. In addition, to identify the fused regions of a chromosome, the Atlantic cod genome assembly (gadMor3.0) was mapped towards the PacBio genome assembly using Minimap2 v2.26 with the settings -asm5. Lastly, we mapped the ONT reads towards the PacBio genome assembly using minimap2. Dotplots were also generated for PacBio vs ONT draft genome assembly comparisons using the D-genies online platform [137].

### Genomic differentiations along the chromosomes (F_ST_, D_XY,_ and π) for the Arctic gadids

To evaluate genetic divergence and/or nucleotide similarity along chromosomes between Arctic cod and polar cod, we took advantage of population-level data generated in Maurstad et al. [54] and Hoff et al. [53]. A total of 14 Arctic cod and 14 polar cod samples (Additional file 1, Table S13) were selected for a joint variant calling using Arctic cod as the reference, as well as Atlantic cod [62] (gadMor3.0, NCBI refseq assembly: GCF_902167405.1). First, Illumina pair-end reads were trimmed using Trimmomatic v0.39 [159] using default settings. Mapping was done using the Burrows-Wheeler Alignment Tool v0.7.17 [160] (BWA-MEM algorithm) with default settings. Alignment files for each sample were merged and sorted using SAMtools v1.9 [161]. Reads of duplicated origin were marked using MarkDuplicates v2.22.1 [162]. Variant calling and hard filtering of variant sites were performed using the Genome Analysis Toolkit (GATK) v4.2.0.0 [163, 164]. First, each mapped sample was individually called into GVCFs using the “HaplotypeCaller” tool. Second, GVCFs were imported into a GenomicsDataBase using the “GenomicsDBImport” tool. Joint genotyping was performed using the GenotypeGVCFs tool to produce final VCFs. SNPs were extracted and down-sampled to 100,000 SNPs using “SelectVariants” to generate diagnostic plots for specific filter parameter evaluation. Filtering was done by following the GATK hard-filtering recommendations as well as by manual inspection of the diagnostic plots as suggested in Danecek et al. [165]. After the initial round of filtering, VCFs were further filtered using VCFtools v0.1.16 [165]. Using the resulting SNP variant dataset, we calculated F_ST_, D_XY_ between polar cod and Arctic cod, as well as π for each species using pixy v1.2.6 [72] with a window size of 10,000 bp.

### Phylogenomic placement of Arctic cod

#### Mitochondrial phylogenetics

For our mitochondrial phylogenetic analyses, we included mitochondrial genomes for additional gadid species gathered from NCBI (see Table 1 for an overview) in addition to the assembled mitochondrial genomes for Arctic cod, polar cod, Atlantic cod (NEAC and NCC), Atlantic haddock, burbot, and European hake. Mitochondrial protein-coding genes (PCGs) were identified by MitoFish [166–168], aligned using MAFFT v7.453 [169], and manually inspected and corrected for reading frame shifts before they were concatenated with PhyKIT v1.11.7 create_concat [170] to produce a supermatrix. A maximum likelihood (ML) tree was inferred using IQ-Tree2 v2.2.0 [59] and ModelFinder Plus (MFP) [171] was used to search for the best substitution model under the Bayesian information criterion (BIC). Branch supports were calculated using 1,000 replicates of ultrafast bootstrap approximation (UFBoot) [172]. Pairwise similarity for each PCG was calculated among *Arctogadus glacialis*, *Boreogadus saida,* and the species included from *Gadus* using PhyKIT v1.11.7 [170] (Additional file 2, Fig. S20). In addition, we conducted phylogenetic analysis including only our assembled mitogenomes (see Additional file 5 for details).

#### BUSCO phylogeny

Single-copy orthologous genes from five of the genome assemblies (Arctic cod, polar cod, Atlantic cod (NCC), burbot, and European hake), in addition to Atlantic cod [62] (gadMor3.0, NCBI refseq assembly: GCF_902167405.1) and Atlantic haddock (ENA: ERR1473879), were identified using BUSCO v5.0.0 [133]. BUSCO was run using AUGUSTUS v3.2 [173] applying the lineage dataset Actinopterygii (actinopterygii_odb10) consisting of 3640 BUSCO groups. All nucleotide BUSCO genes found as single-copy and occurring within all the assemblies were extracted and aligned using MAFFT v7.453 [169] with the L-INS-i strategy. Each alignment was trimmed using the smart-gap approach with ClipKIT v1.3.0 [174]. Filtering of alignments included filtering on sequence length, variable sites (VAR sites), and parsimony informative sites (PI sites), as these properties have been shown to affect phylogenetic signals [170]. Sequence statistics were calculated using PhyKIT v1.11.7 [59]. Filtering on sequence length (> 500 bp), VAR sites (> 5%), PI sites (> 2.5%), and percentage of gap sites (< = 30%) removed 555 BUSCO genes (Additional file 2, Fig. S26). Alignments that were above 500 bp, contained more than 5% VAR sites, 2.5% PI sites, and had more than 70% sites without gaps were included for downstream analysis.

Phylogenetic placement of Arctic cod using BUSCO genes was inferred under both concatenation and gene tree approaches. Gene trees were first inferred using IQ-Tree2 v2.2.0 [59] under the GTR + I + G substitution model [65]. Tree support for each gene was calculated using 1,000 replicates of UFBoot approximation. Species tree estimation based on the gene trees produced in IQ-Tree2 was inferred under an MSC analysis conducted in ASTRAL-III v5.7.8 [79]. Bootstrap support was estimated using 100 replicates of multi-locus bootstrap based on the bootstrap trees produced with IQ-Tree2. Discordance among BUSCO gene trees was visualized using quartet frequencies with DiscoVista [170] while specifying European hake, burbot, and Atlantic haddock as outgroups. The same BUSCO genes used to infer the species tree were concatenated into a supermatrix using PhyKIT v1.11.7.

#### Chromosomal number and northward distribution in codfishes

We investigated the relationship between chromosomal number vs. preferred sea temperature as well as latitudinal distribution, among the 13 Gadiformes that were included in the mitochondrial phylogeny, by fitting a phylogenetically informed linear regression implemented by the R package phylolm [175]. For this test, we used the minimum number of chromosomes reported for those species that exhibited variable intraspecies chromosomal number, i.e., polar cod and Arctic cod [48, 49]. This was tested against the preferred sea temperature range for each species (estimated as mean °C), where data were obtained from www.fishbase.se/search.php (accessed 23.11.2025) [176, 177]. Chromosomal number was also tested against northernmost as well as southernmost distribution limits, i.e., defining each species' restriction on how far south they are found, thus reflecting their northerly distribution. The function for running the test as follows; phylolm(Chromosomes_n ~ North_dist, data = Data, phy = Tree_rooted, model = "BM"), where Chromosomes_n were haploid chromosome numbers, and North_dist were northernmost as well as southernmost distribution boundaries in two separate tests (Additional file 1, Table S9). All models were fitted assuming a Brownian motion (BM) model of trait evolution. Option Phy = Tree_rooted was set to the mitochondrial phylogenetic tree, which was rooted using European hake. Latitudinal coordinates were obtained for all species via www.fishbase.se/search.php (accessed 08.09.2025). After obtaining the test results, the p-values were adjusted by the following function: p.adjust(pvals, method = "bonferroni"), where *pvals* were listed p-values for each test.

### Identification and characterization of antifreeze glycoprotein gene clusters, and their genomic location in the Arctic codfishes

A two-step method was used to identify the antifreeze glycoprotein genes and their location in the genomes of polar cod. First, the entirety of the *afgp* genomic regions for polar cod previously released by Zhuang et al. [76] (NCBI accession MK011258, MK011259, and MK011260) were mapped to the polar cod draft ONT genome assembly (haplotype 1) using minimap2 [130], applying the setting: -x asm5. The resulting paf files were manually inspected for best hit in the genomes, based on length of matching bases and MAPQ. Secondly, we ran miniprot v0.18 [142] using the protein-coding sequences of the *afgp* genes from polar cod (as mentioned above) as query, and the ONT draft assemblies (haplotype 1) for polar cod and Arctic cod as target. Settings -Iut16 and –gff were applied. The results were then visually inspected in the Integrative Genomics Viewer [178]. In addition, the gene annotation results for Arctic cod genome assembly were inspected for *mak16* (a gene flanking the *afgp* cluster I in Atlantic cod and Atlantic tomcod [77]) for extra confirmation of which gene cluster was identified.

To shed light on the origin of the three known *afgp* gene clusters in polar cod, we generated Maximum Likelihood (ML) trees using the Tamura-Nei (1993) model [179] and 100 standard bootstrap replicates in MEGA12 [180]. First, the coding sequence of all *afgp* genes identified in polar cod in the present study, as well as the previously identified *afgp* genes [76], were aligned using MUSCLE [181]. Before generating the ML tree, the alignment was manually trimmed to contain a majority of informative sites.

To investigate the potential molecular mechanisms behind the *afgp* expansion in polar cod, we characterized the TE landscape surrounding the three *afgp* clusters on chromosomes 1, 2, and 14. First, we plotted the repeat densities on the three chromosomes within non-overlapping sliding windows of 500 bp spanning each *afgp* gene cluster, including flanking regions of ± 25,000 bp. Second, we visualized putative syntenic TE insertions in the three clusters (filtered on TE families that were shared by at least two *afgp* clusters) using geneviewer v0.1.10 [182]. Recurring TE insertions flanking *afgp* genes or the ends of different clusters were manually investigated to validate if the insertions represented overlapping regions of the consensus sequences, and that the insertions were not false positives (see *Repeat annotation of Arctic codfishes* above). Finally, we detected a simple tandem repeat (STR) (see Additional file 2, Fig. S32) consisting of three repeating units of ~ 2,000 bp that resembled the *afgp* *6* gene sequence detected in Baalsrud et al. [46] (~ 68% identity across each repeating unit). This repeat was classified as an *afgp*-like repeat.

## Supplementary Information

Additional file 1: Supplementary tables [48, 49, 53, 54, 60–62, 176, 177, 186–193].

Additional file 2: Supplementary figures [46, 53, 59, 65, 72, 76, 79, 142, 148, 153, 154, 170, 174, 178, 194].

Additional file 3: Supplementary note 1. Description of genome assembly of burbot, and long-read mitogenomes for six codfishes [60, 61, 134, 135, 195–198].

Additional file 4: Supplementary note 2. Description of validation of chromosomal fusions and inversions using ONT genome assemblies and reads, and further details on macro-synteny and identification of chromosomal rearrangements in codfishes [48, 49, 53, 54, 62, 193].

Additional file 5: Supplementary methods [59, 62, 78, 123–127, 129–135, 137, 138, 140–145, 160, 161, 167–171, 174, 178, 194, 199–224].

## Data Availability

All raw sequences, as well as genome assemblies generated within this study, have been deposited in the European Nucleotide Archive (ENA) at EMBL-EBI under the bioproject PRJEB77069 [183]. More specifically, this included the following genome assemblies, with their respective ENA accession numbers: GCA_964260595.1 for *Arctogadus glacialis* (arcglaasm) [183], GCA_964260565.1 for *Boreogadus saida* (borsaiasm) [183], GCA_964260575.1 for *Gadus morhua *‘NCC’ (gadmorasm) [183], GCA_964260585.1 for *Lota lota* (lotlotasm) [183], and GCA_964260605.1 for *Merluccius merluccius *(mermerasm) [183]. Additionally, the genome assembly for Atlantic cod (NEAC) was accessed by ENA nr GCA_902167405.1 for *Gadus morhua* ‘NEAC’ (gadMor3.0) [62]. Moreover, additional annotations, as well as two ONT draft assemblies for *Arctogadus glacialis* (fArcGla1.1.hap1.draft.fa.gz and fArcGla1.1.hap2.draft.fa.gz) and *Boreogadus saida *(fBorSai1.1.hap1.draft.fa.gz and fBorSai1.1.hap2.draft.fa.gz), are available at: 10.5281/zenodo.17475568 [136]. Finally, the population datasets used can be found under ENA project PRJEB88972 for Arctic cod [184], and PRJEB77070 for polar cod [185].
