# Supplementary material for: Rapid genome modifications including chromosomal fusions and large-scale inversions are key features in Arctic codfish species
Source: Genome Biol. 2026 Feb 16;27:100. doi: 10.1186/s13059-026-03975-6 (PMC13011446; doi:10.1186/s13059-026-03975-6)
Supplement: Supplementary file 2 — Additional file 2: Supplementary figures [46, 53, 59, 65, 72, 76, 79, 142, 148, 153, 154, 170, 174, 178, 194]. [file 13059_2026_3975_MOESM2_ESM.docx]

**Supplementary figures**


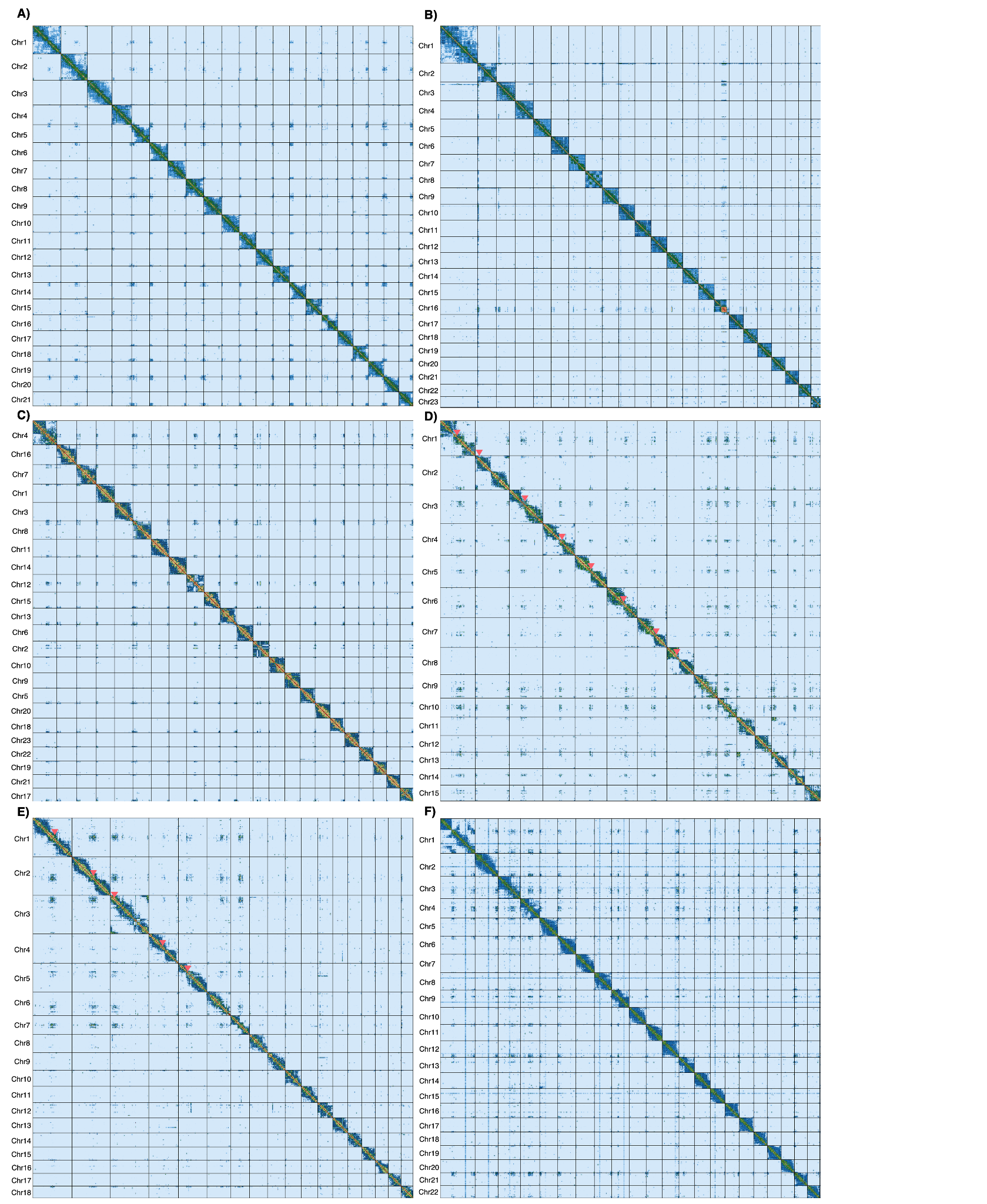


**Fig. S1.** Hi-C contact points are shown as a heatmap on the super-scaffolds of each final genome assembly of the six Gadiform fishes. Putative centromere regions are annotated with red triangles for the fused chromosomes in Arctic cod and polar cod. **A)** European hake, **B)** burbot**, C)** Atlantic cod (NCC), **D)** Arctic cod, **E)** polar cod, and **F)** Atlantic haddock.

**
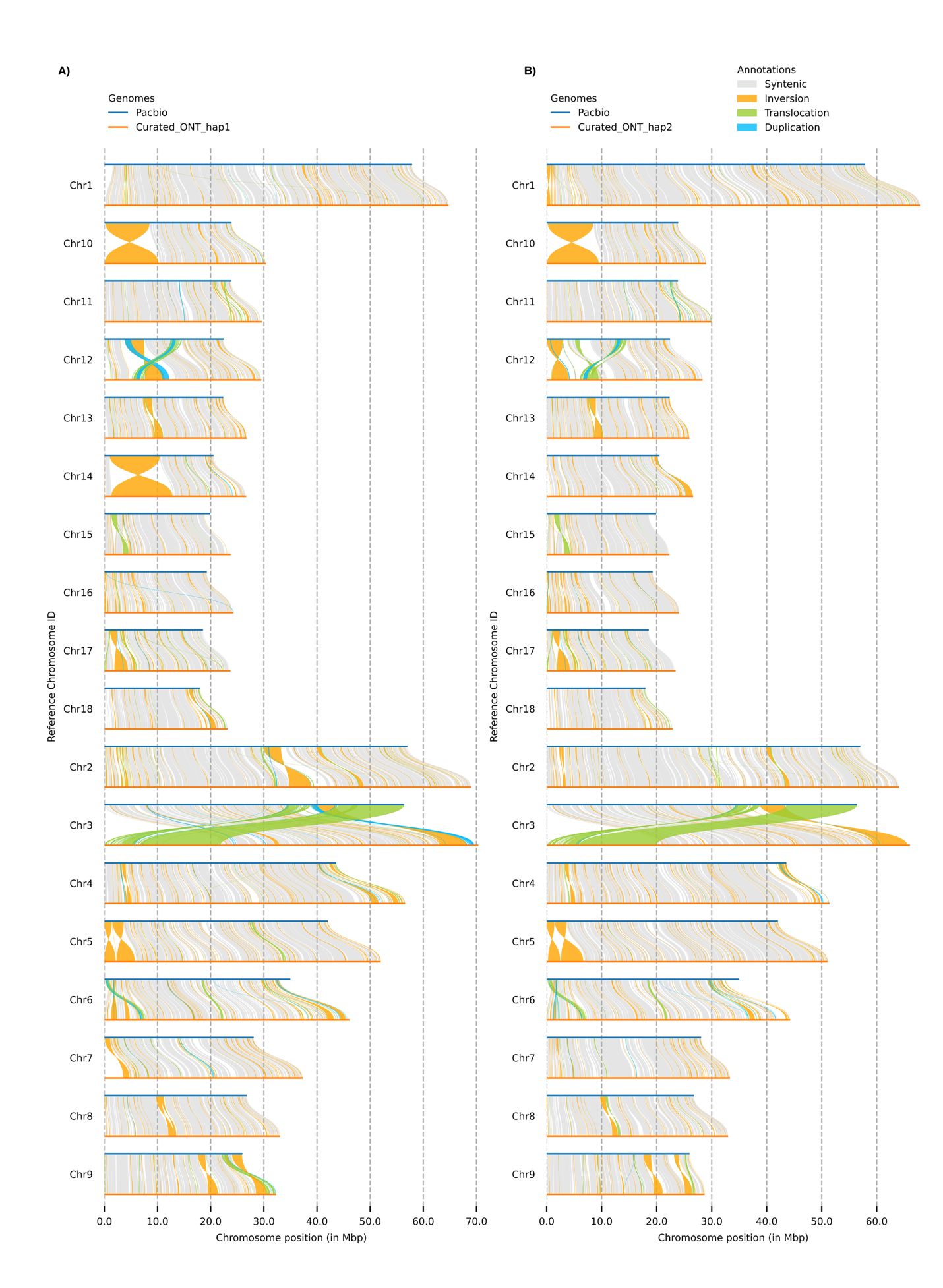
**

**Fig. S2.** Chromosomal synteny between PacBio reference genome and ONT draft assembly for **A)** haplotype 1, and **B)** haplotype 2 for polar cod.


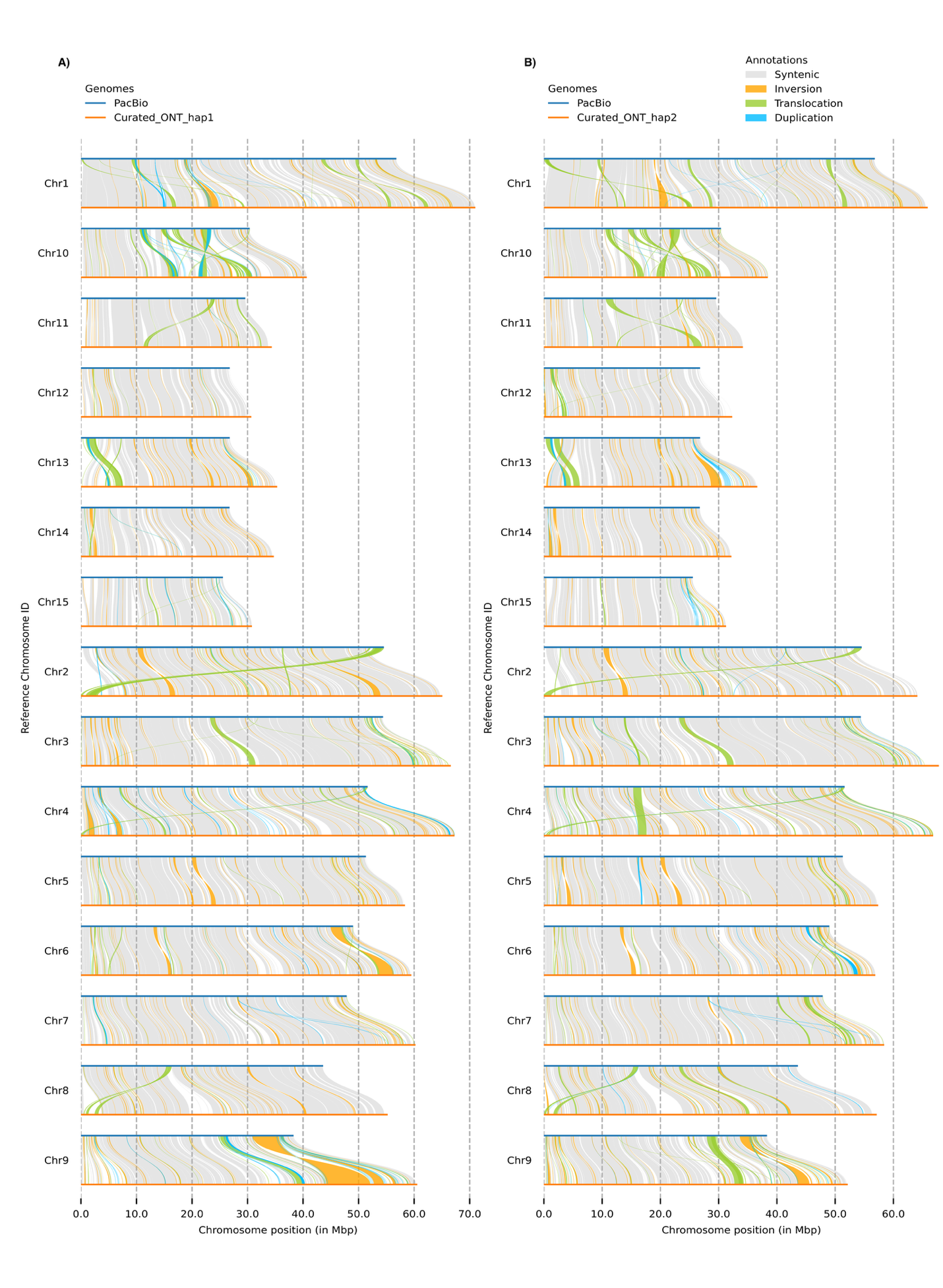

**Fig. S3.** Chromosomal synteny between PacBio reference genome and ONT draft assembly for **A)** haplotype 1, and **B)** haplotype 2 for Arctic cod.


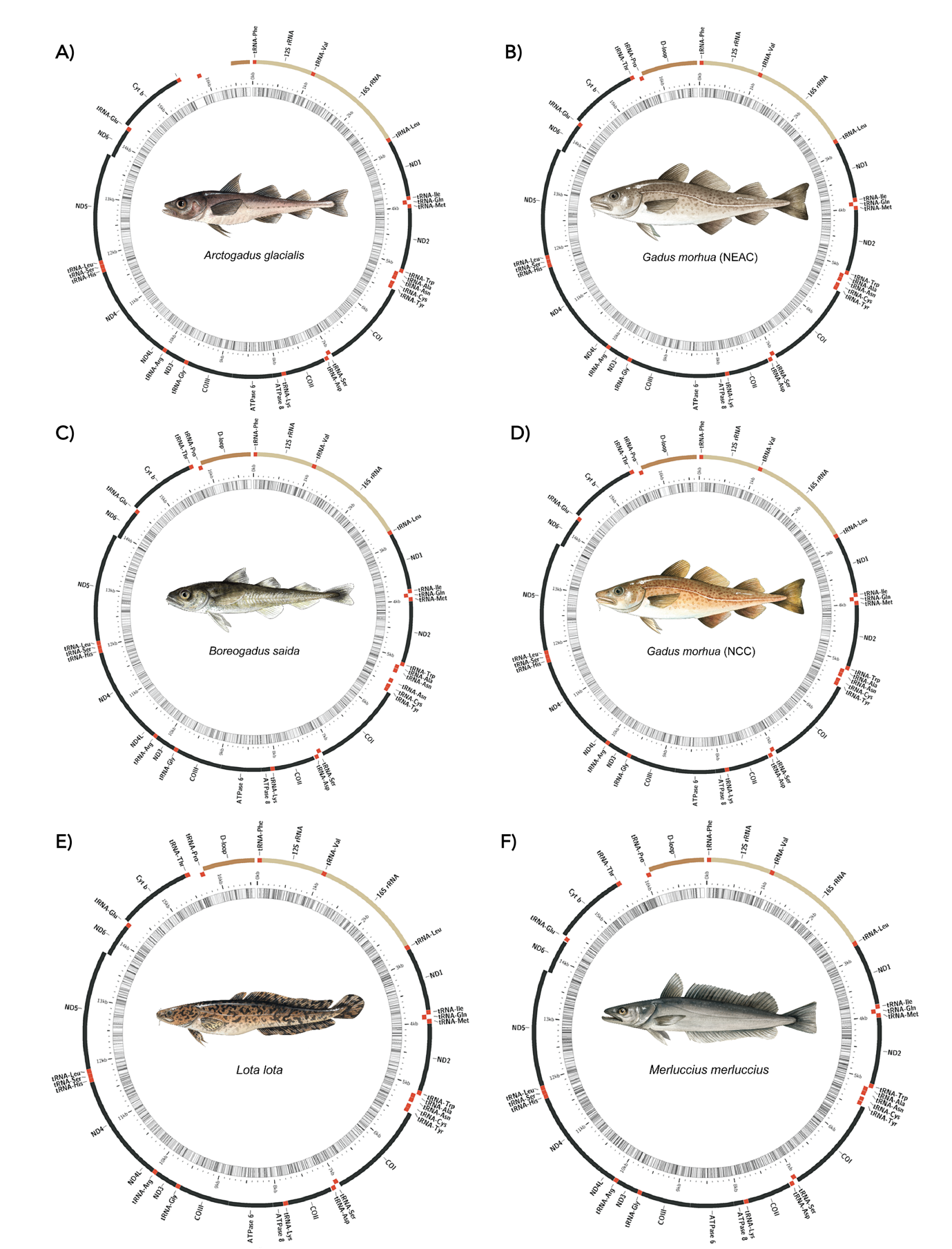


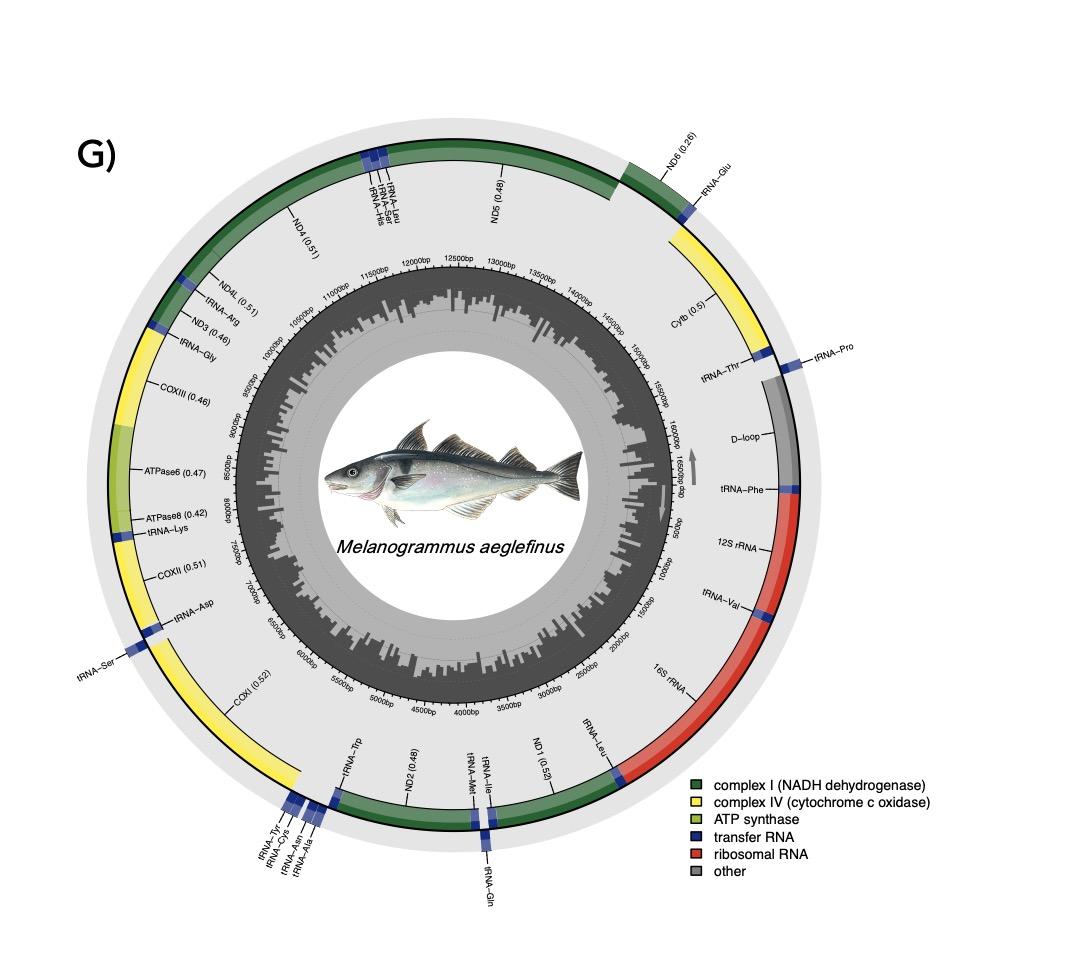


**Fig. S4.** Mitofish annotated mitogenomes for **A)** Arctic cod, **B)** Atlantic cod (NEAC), **C)** polar cod**, D)** Atlantic cod (NCC**), E)** Burbot**, F)** European hake, and **G)** Atlantic haddock. In **A-F),** the inner band represents GC-content. All fish illustrations by Alexandra Viertler.


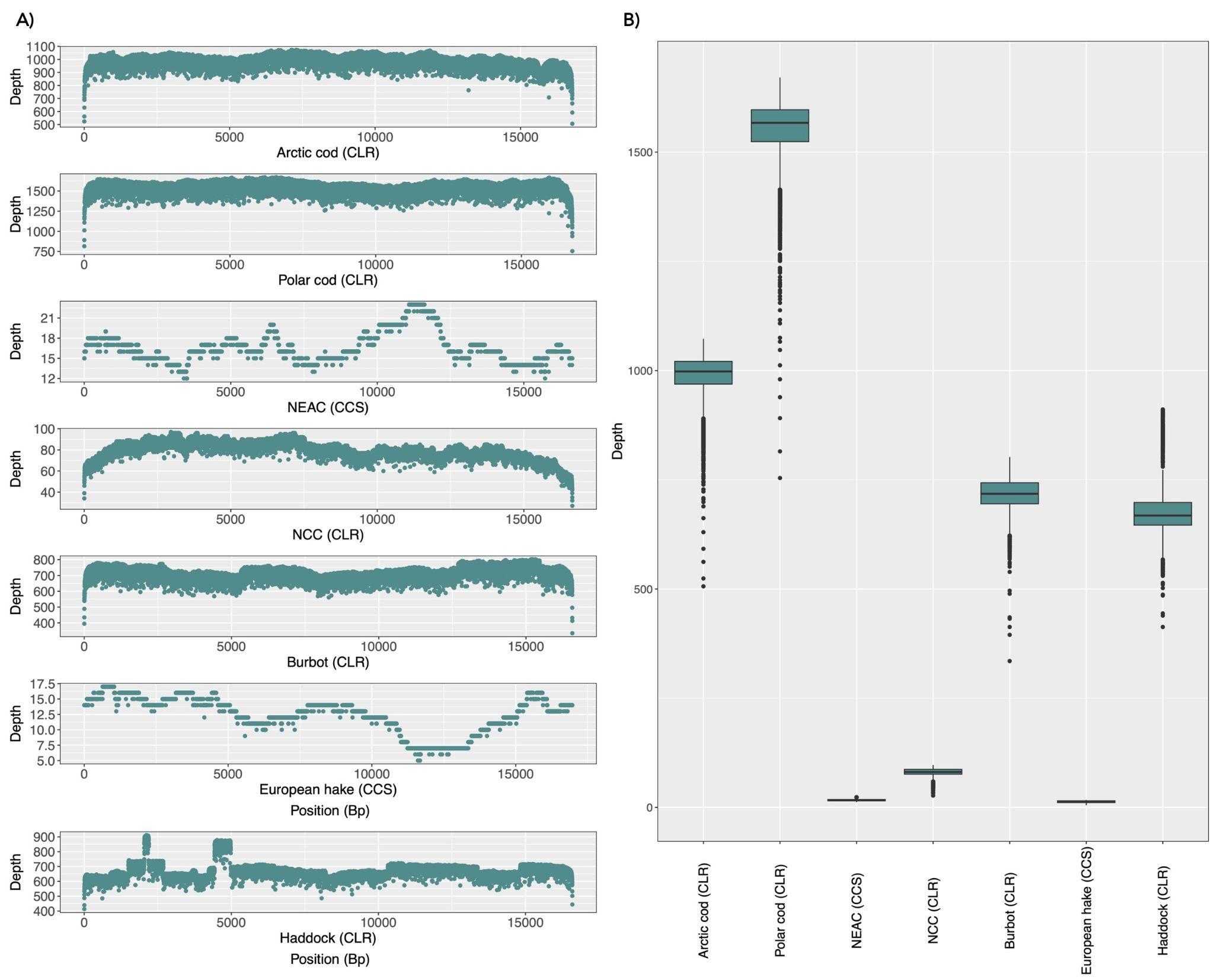


**Fig. S5.** Depth distribution of PacBio long-reads mapped against the assembled mitogenomes. **A)** Depth of the reads along the mitogenomes per base and **B)** Depth visualized as boxplots per assembly.


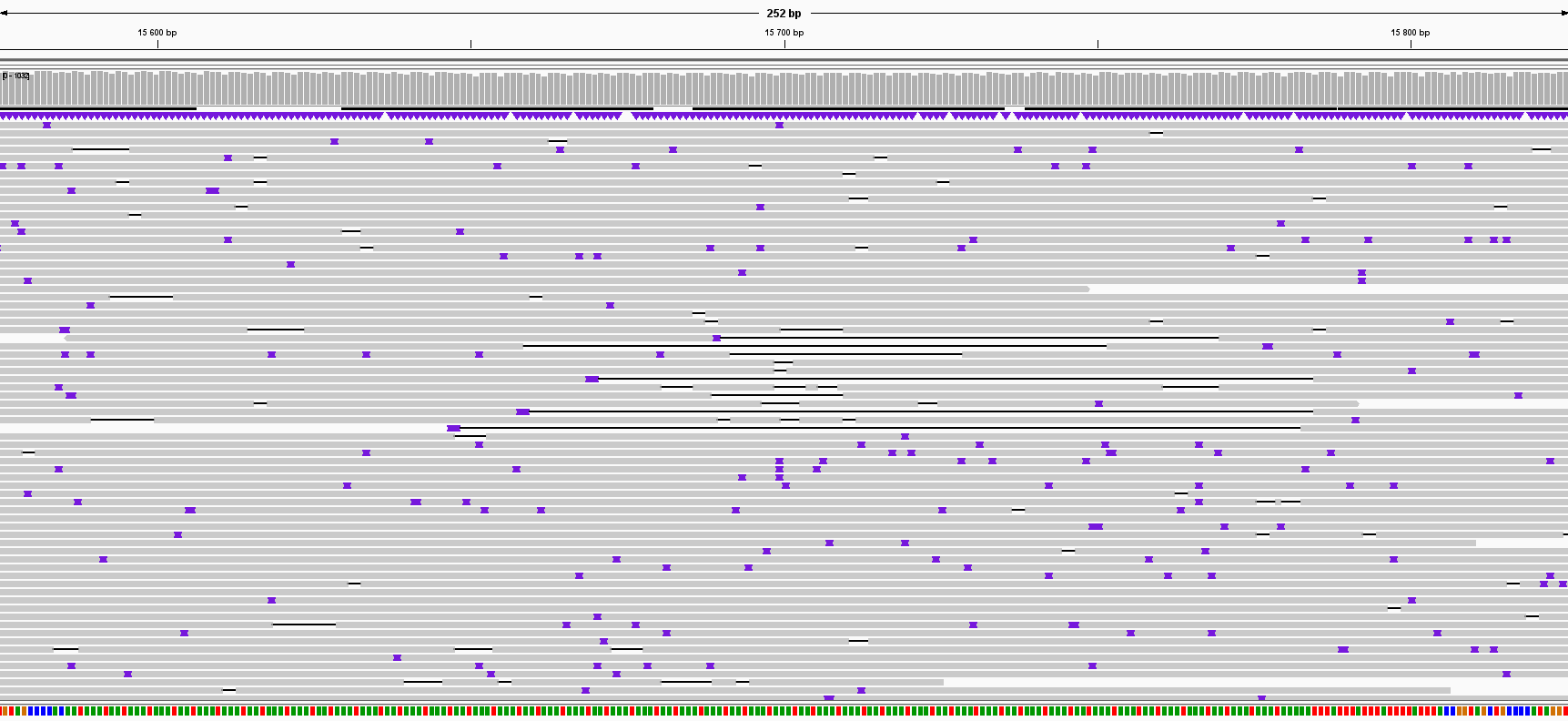


**Fig. S6.** Examples of potential heteroplasmic reads along a repeat within the Arctic cod mitochondrial genome from position 15575 - 15825 visualized in Integrative Genomics Viewer [178]. Purple points and black bars represent insertions or deletions, respectively.


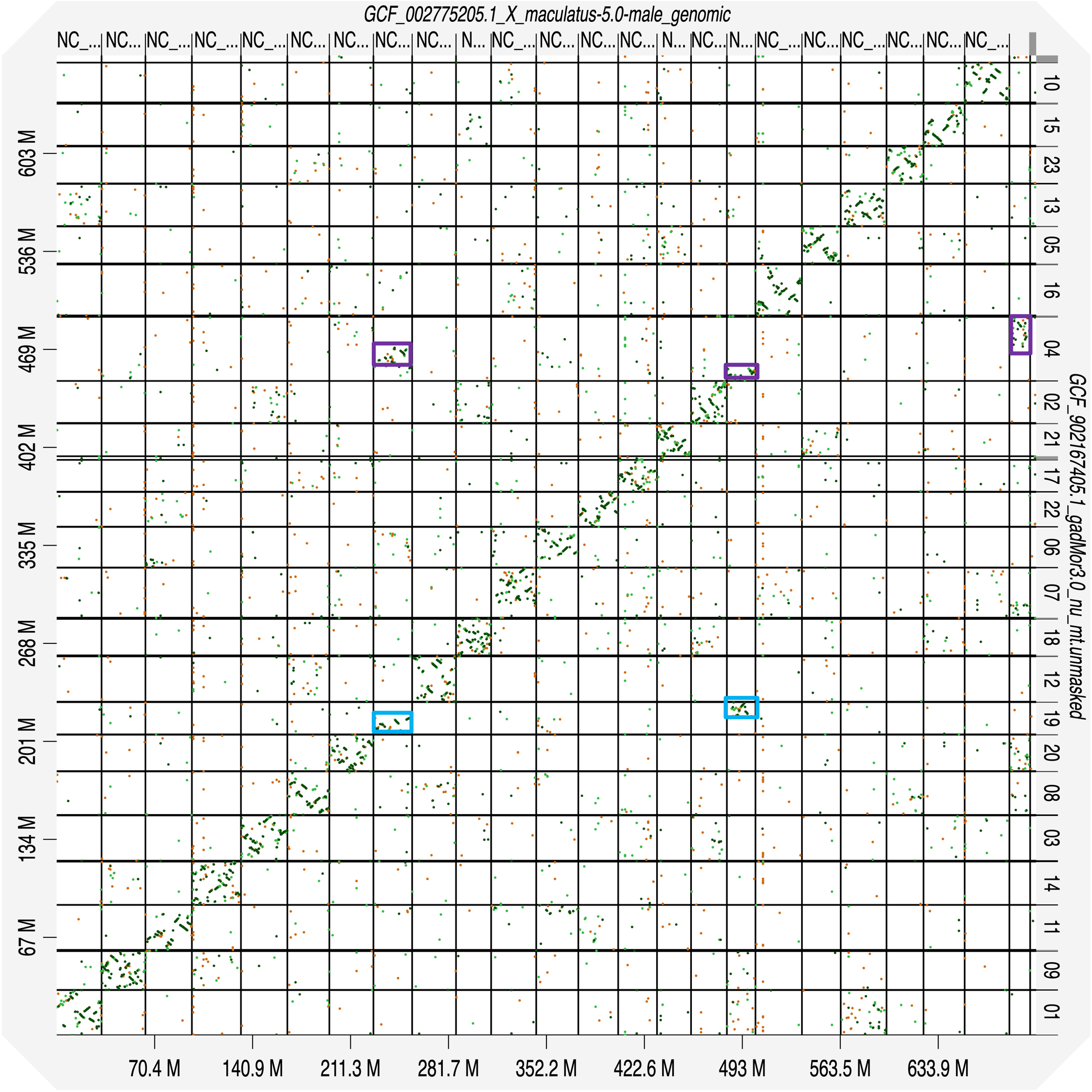


**Fig. S7.** Dotplot between Atlantic cod (NEAC) and platyfish (n=24) genome assembly reveals mostly syntenic relationships between chromosomes. Two possible fused chromosomes in Atlantic cod (LG04 and LG19) compared to platyfish marked with squares.


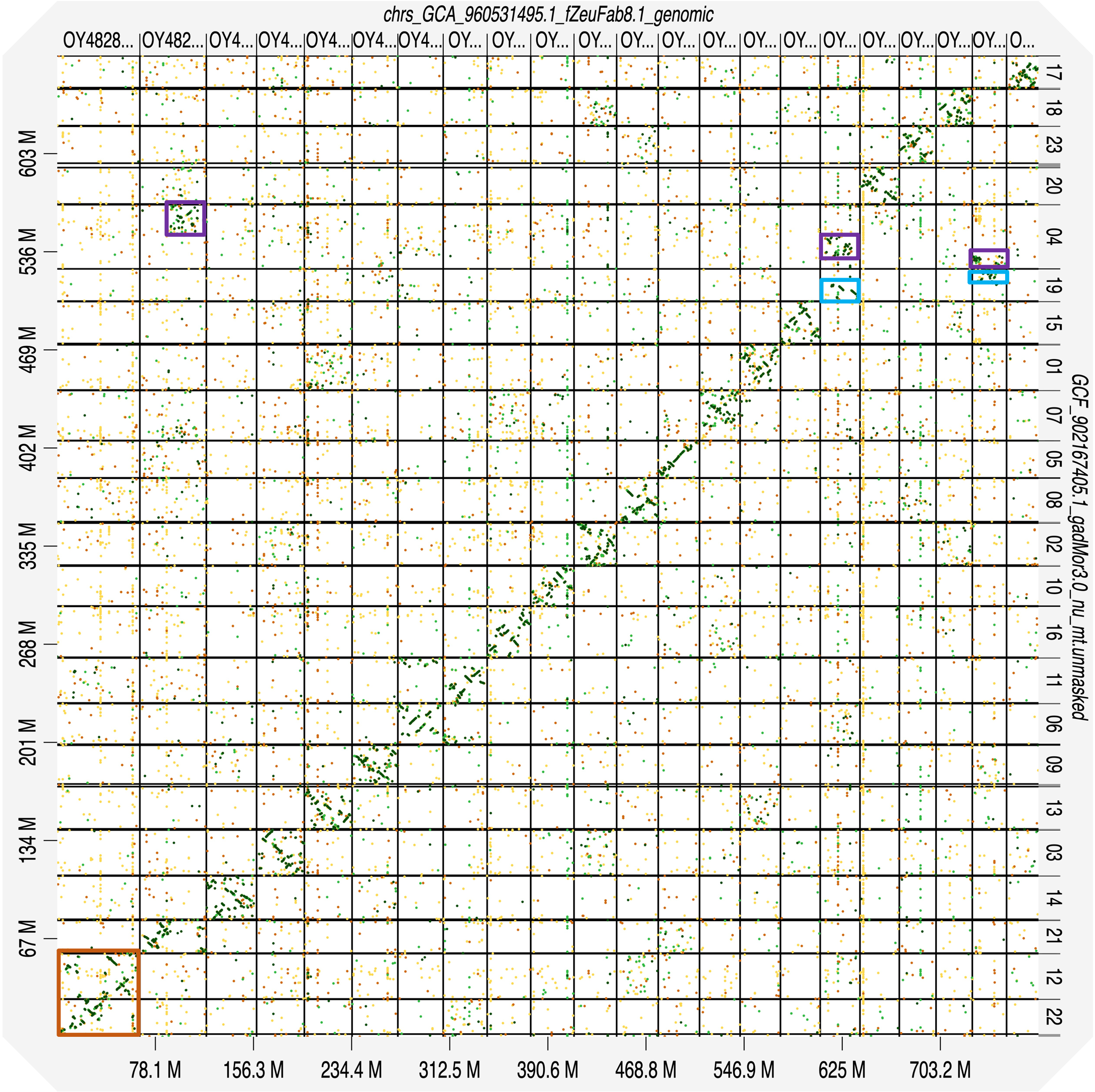


**Fig. S8.** Dotplot between Atlantic cod (NEAC) and John Dory (n=22) genome assembly. Two possible fused chromosomes in Atlantic cod (LG04 and LG19) compared to John Dory, marked with squares. A fission (Atlantic cod) or fusion (John Dory) marked in orange.


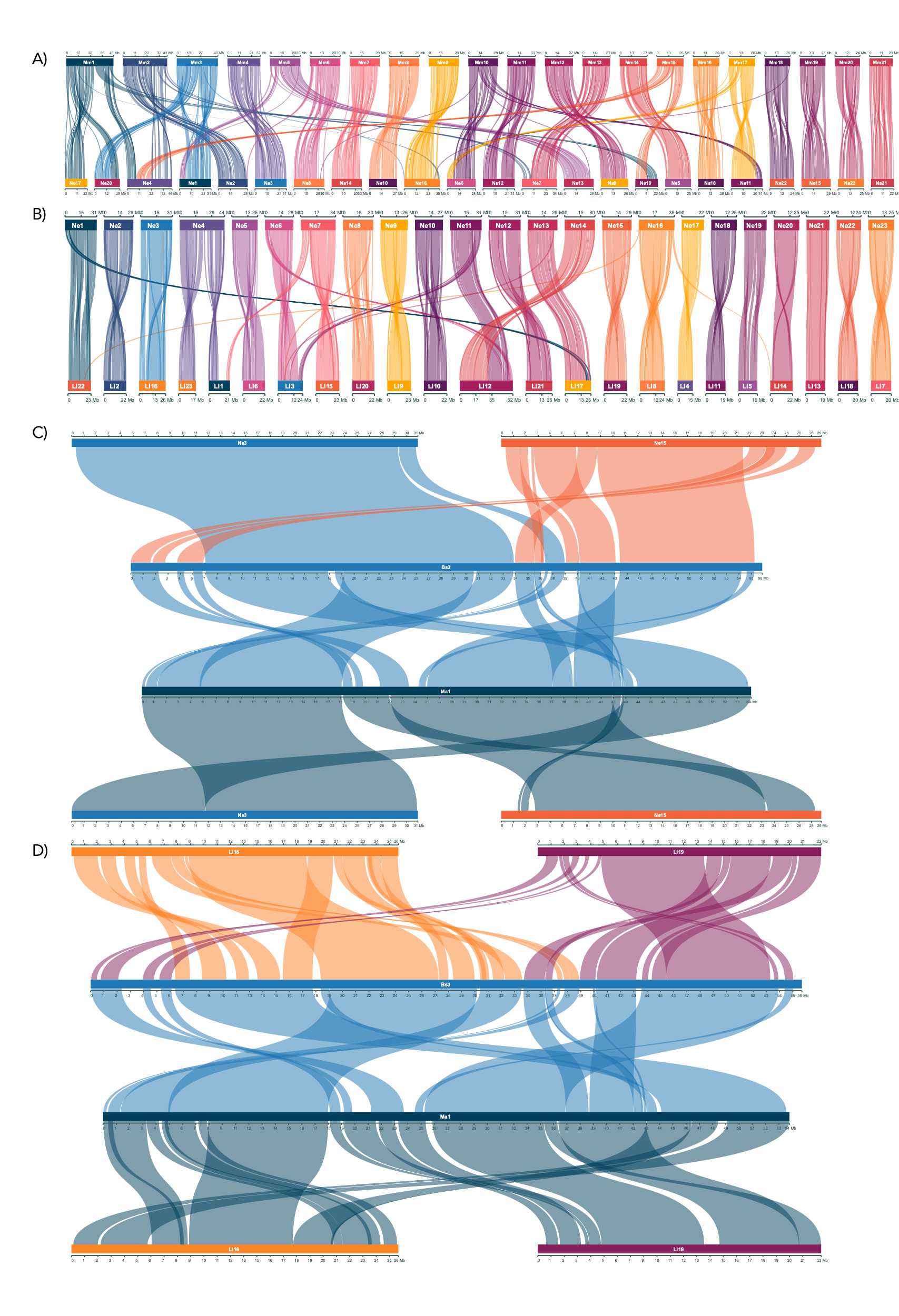


**Fig. S9.** Chromosomal synteny based on gene order between Atlantic cod (NEAC) and **A)** European hake, and **B)** burbot. Chromosomal synteny between homologous chromosomes polar cod Bs3 and Atlantic haddock Ma1, as well as homologous chromosomes in **C)** Atlantic cod (Ne3 and Ne16), and **D)** burbot (Ll16 and Ll19).

**
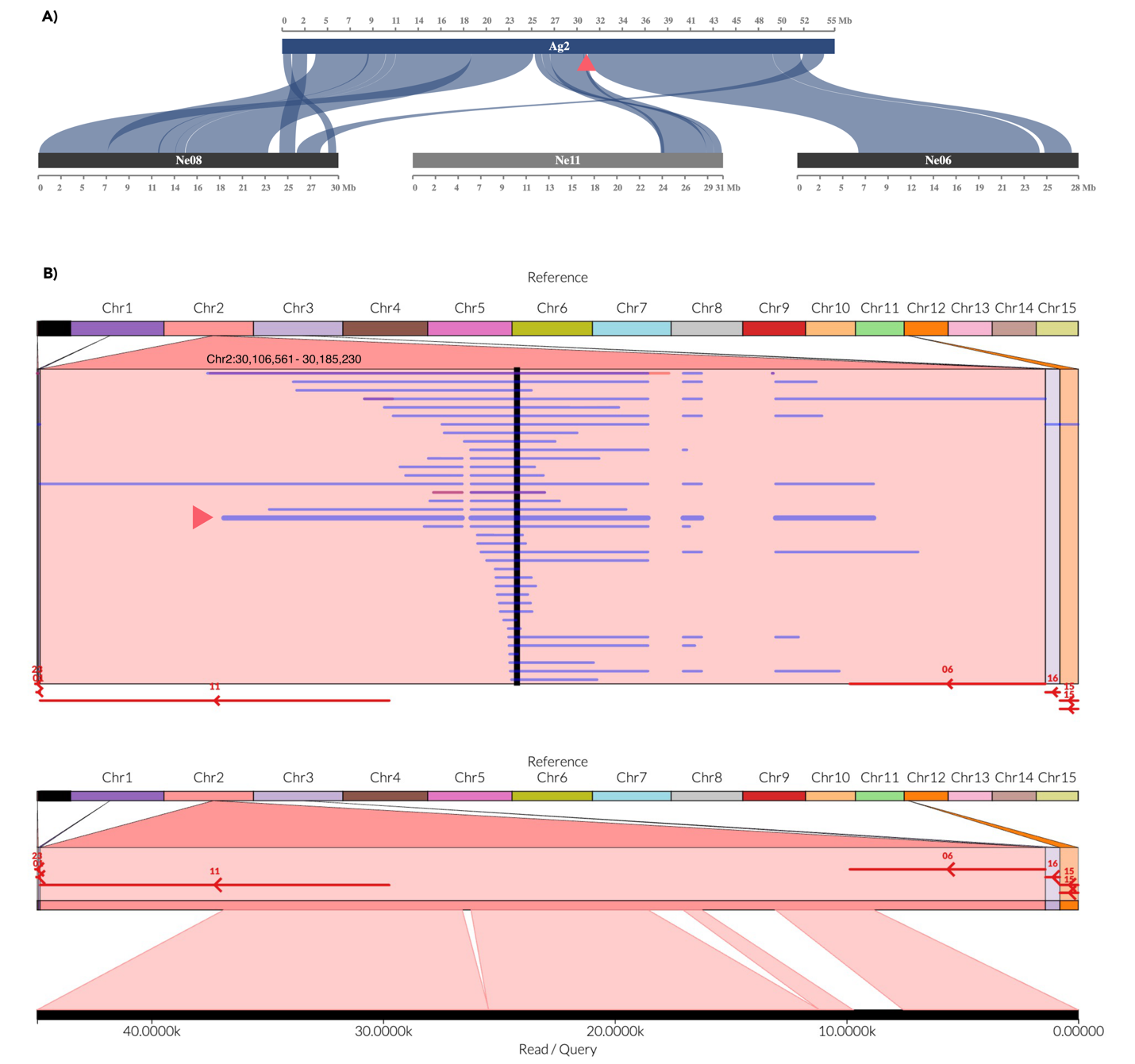
**

**Fig. S10**. **A)** Macro-synteny between the fused chromosome 2 in Arctic cod and the homologous chromosomes in Atlantic cod (Chr6, 8, and 11). Fusion/translocation point between Atlantic cod Chr11 and Chr6 marked with a red arrow**. B)** Top: ONT reads mapped back to the PacBio reference genome. Red bars with naming at the bottom show the homologous chromosomes in Atlantic cod when mapped to the Arctic cod PacBio genome assembly. The panel shows multiple reads that span regions homologous to the end of Atlantic cod 11 and the beginning of Atlantic cod Chr6, suggesting that this region is continuous in Arctic cod assembly.
Bottom: The read indicated with a red arrow in the top panel is shown, displaying one exemplary read crossing the fusion/translocation point.


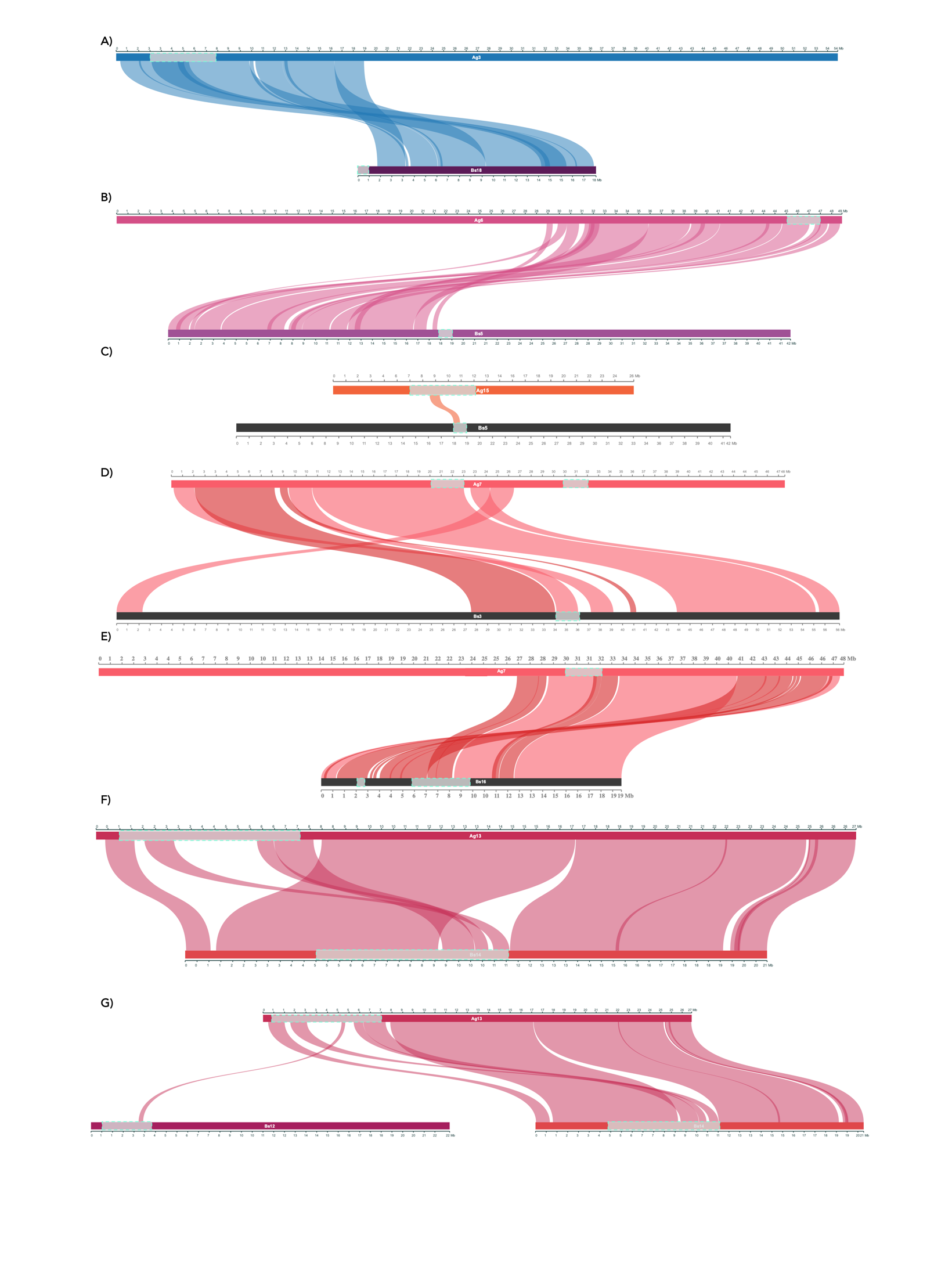


**Fig. S11.** Chromosomal synteny between homologous chromosomes harboring species-specific inversion in Arctic cod and polar cod. **A)** Ag3 - Bs10 with no overlap detected between inversions. The beginning of Bs18 is homologous to the end of Ag4, harboring no inversion in Arctic cod (Fig. 2B). **B)** Ag6 - Bs5, no overlap detected between inversions. **C)** Between the homologous regions harboring inversions on Arctic cod Ag15 and polar cod Bs5, a small overlapping genomic region was detected. **D)** No overlap between inversions was observed between Ag7 - Bs3. **E)** The inversions on Arctic cod Ag7 (~30 - 32 Mb) and polar cod Bs16 (~6 - 9.8 Mb) were found to share one breakpoint region**. F)** We found that the homologous genomic regions of inversions on Arctic cod Ag13 (~1 - 7 Mb) and polar cod Bs14 (~5 - 11 Mb) overlap to some extent. **G)** We additionally found a small genomic region of the inversion on Arctic cod Ag13 to be homologous to the region within a polymorphic inversion on Bs12 in polar cod.


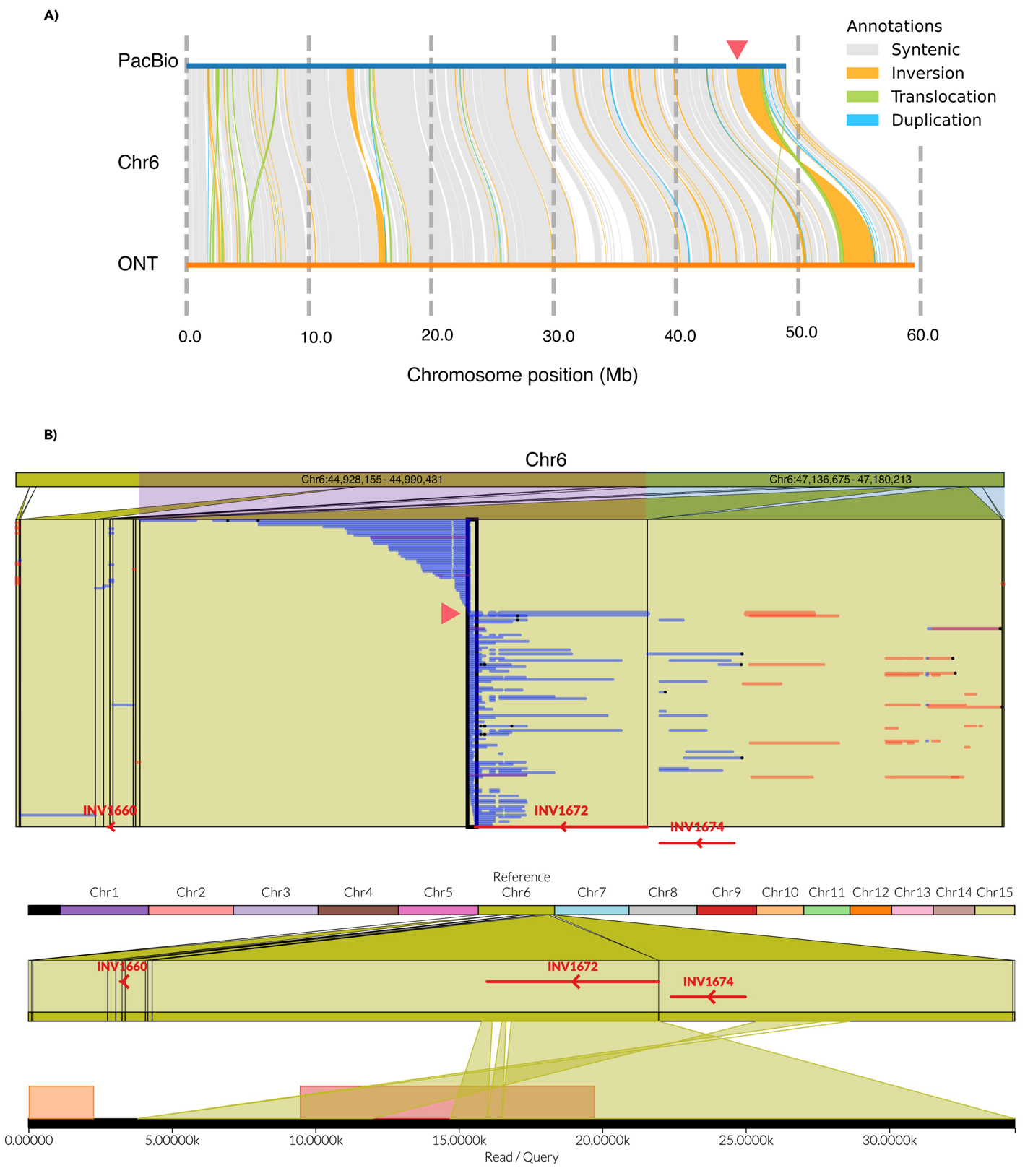

**Fig. S12. A)** Intraspecies chromosomal inversion in Arctic cod, on chromosome 6, verified between the ONT draft assembly (haplotype 1) and the PacBio reference genome, using SyRI. The first breakpoint is marked with a red arrow. **B)** Top: ONT reads mapped back to the PacBio reference genome. Two chromosomal locations are shown, making up the first and second breakpoints of the inversion. Red bars with inversion names at the bottom show the inversions detected by SyRI between PacBio and ONT assembly (haplotype 1). A clear break in read mapping is shown for the first inversion breakpoint (beginning of INV1672). Bottom: The read indicated with a red arrow in the top panel is shown, the read maps to the beginning of INV1672 and after the second breakpoint of the inversion (the end of INV1674).


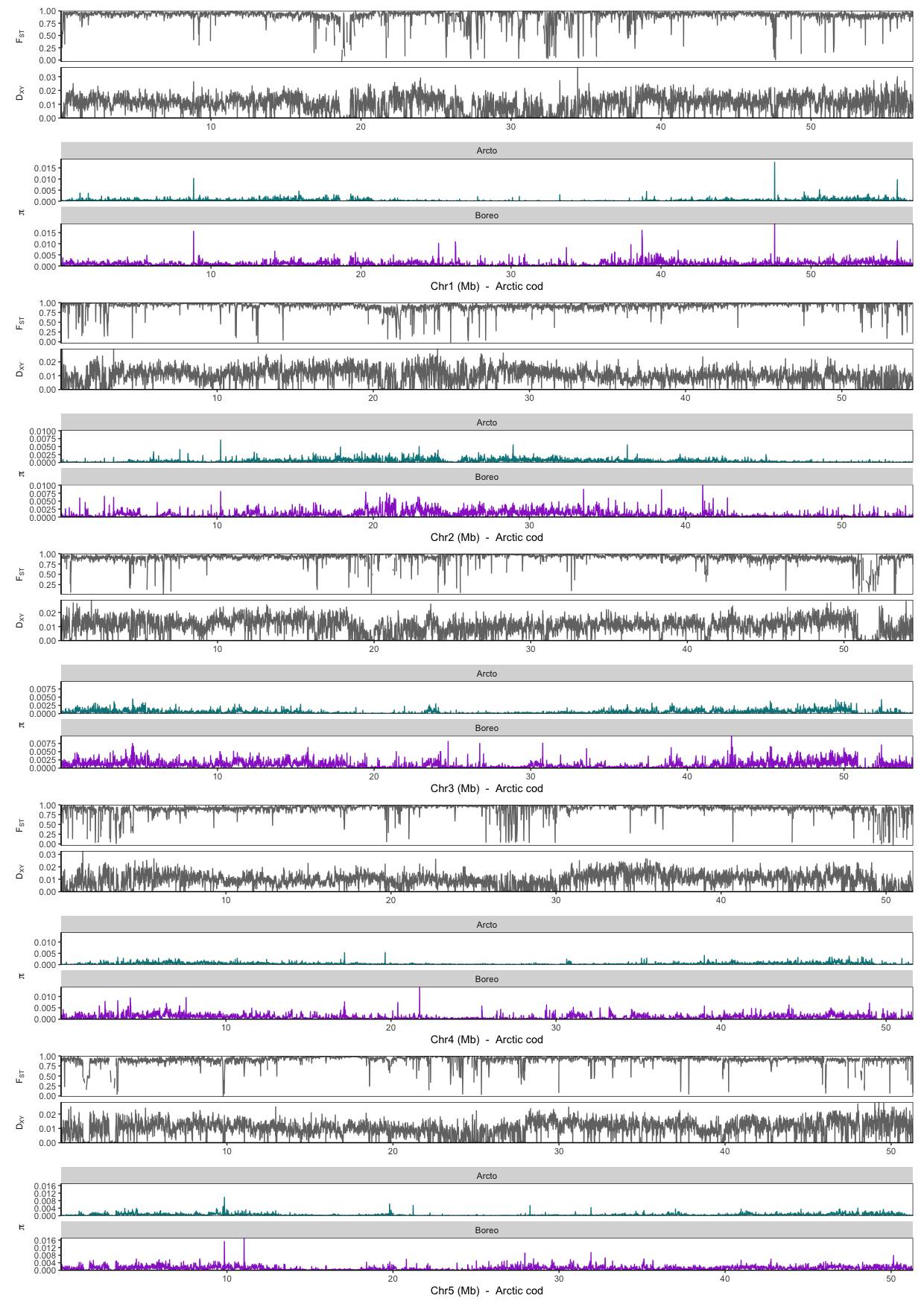


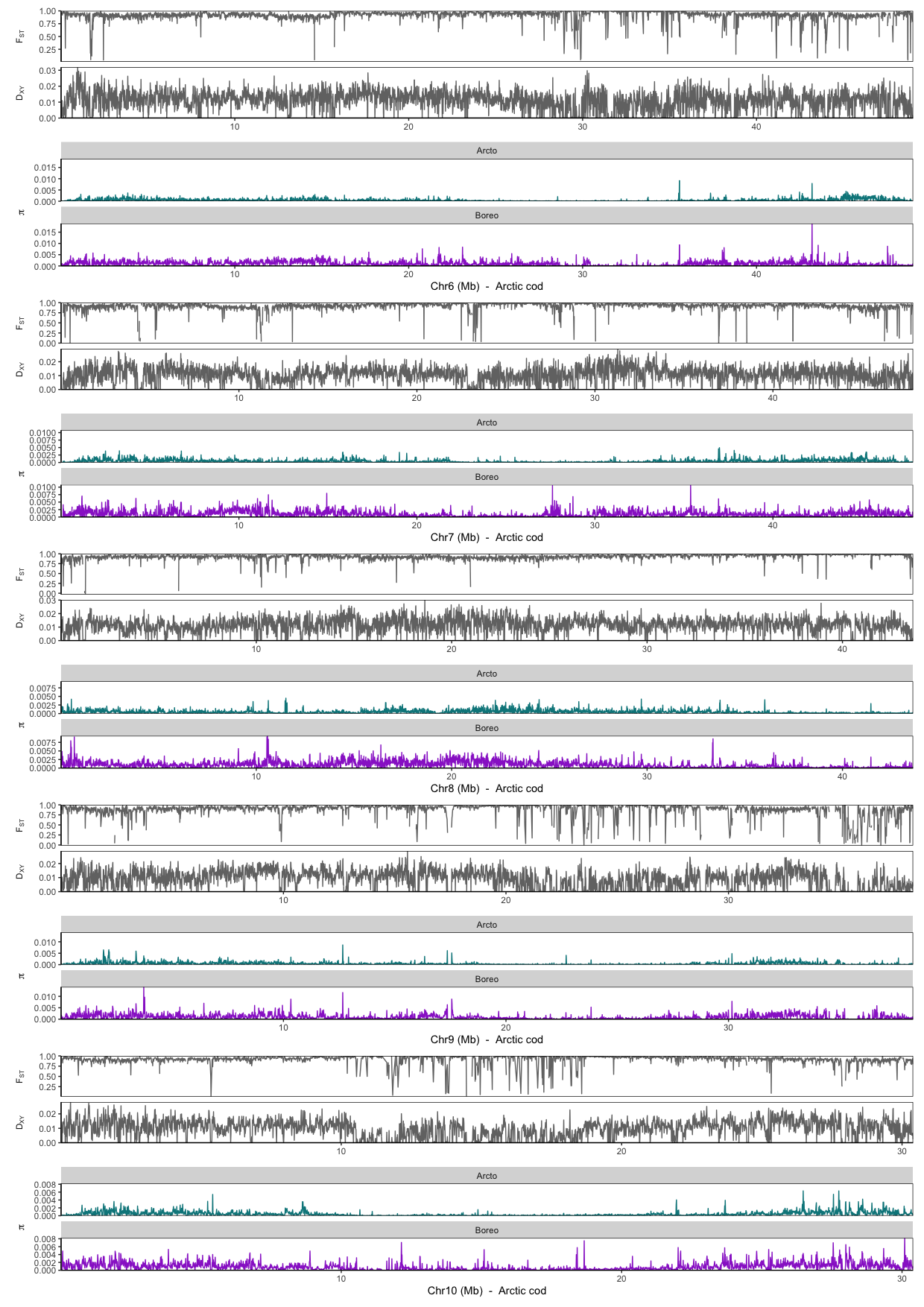


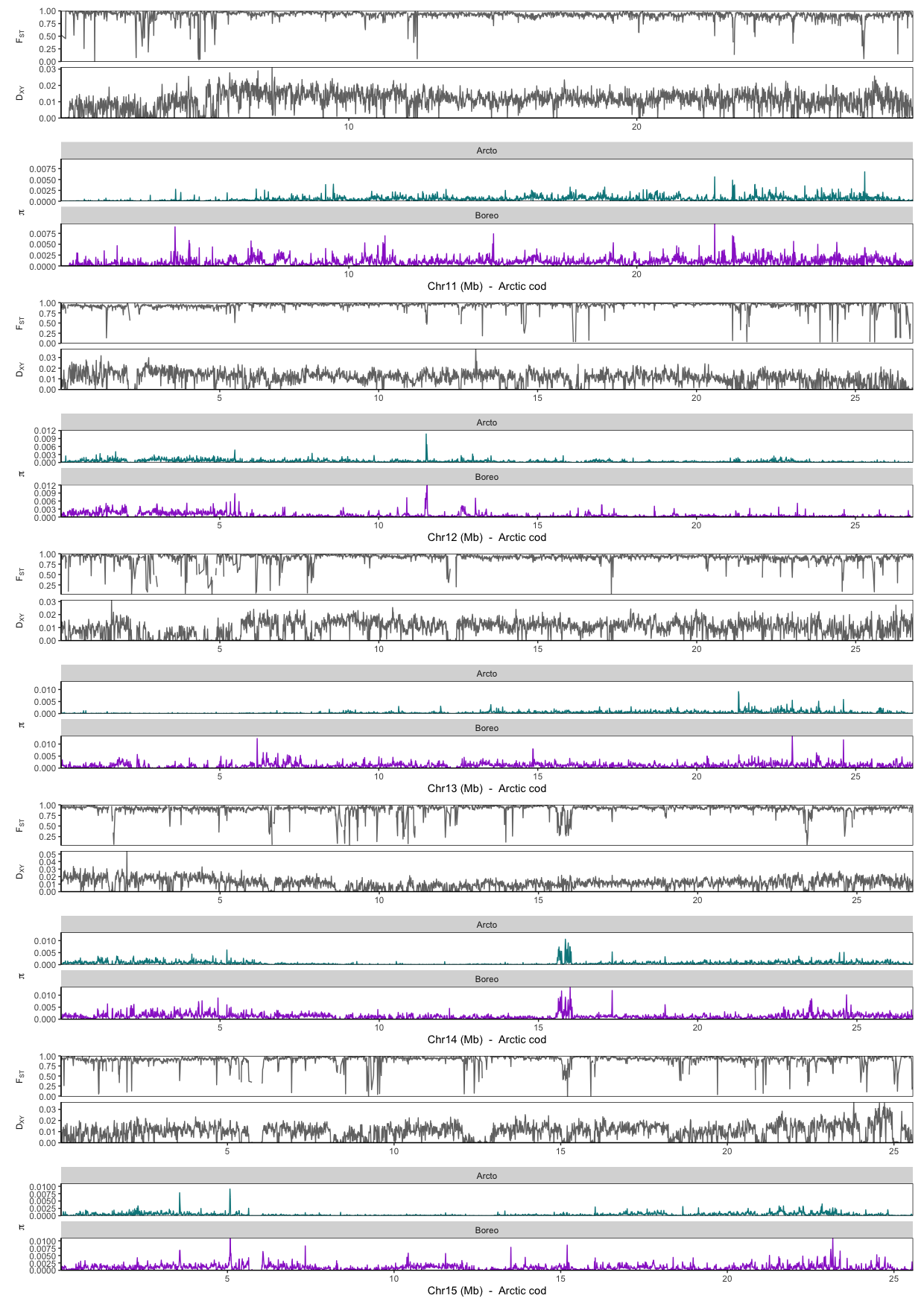


**Fig. S13.** Intraspecies genetic differentiation and nucleotide diversity estimated using pixy [74] between Arctic cod and polar cod using the Arctic cod genome assembly as a reference, for chromosomes 1 – 15 (i.e., Ag1-15).

**
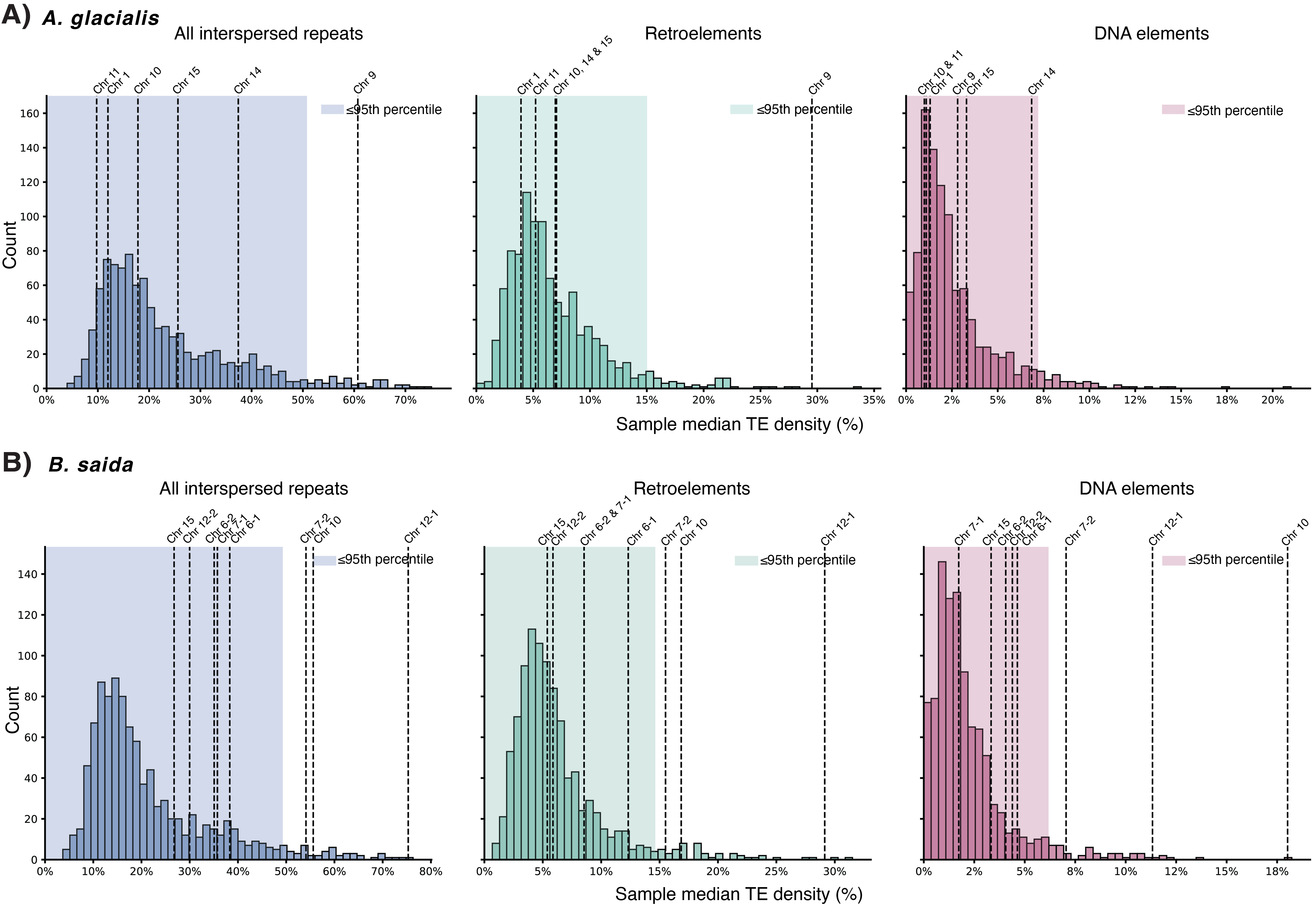
**

**Fig. S14.** Permutation test of repeat densities in breakpoints vs. non-breakpoint region pairs. Distribution of median TE densities for 1,000 randomly drawn pairs of 100 kb non-breakpoint regions in **A)** *A. glacialis* and **B)** *B. saida.* Median densities (%) of non-breakpoint pairs are shown as histograms, partitioned into all interspersed repeats (blue), retroelements (green), and DNA elements (red). The 95^th^ percentiles of each distribution are highlighted in the same colors. Median densities of the inversion breakpoint pairs of overlapping inversions in *A. glacialis* and *B. saida* are displayed as vertical dotted lines labelled above each line.

**
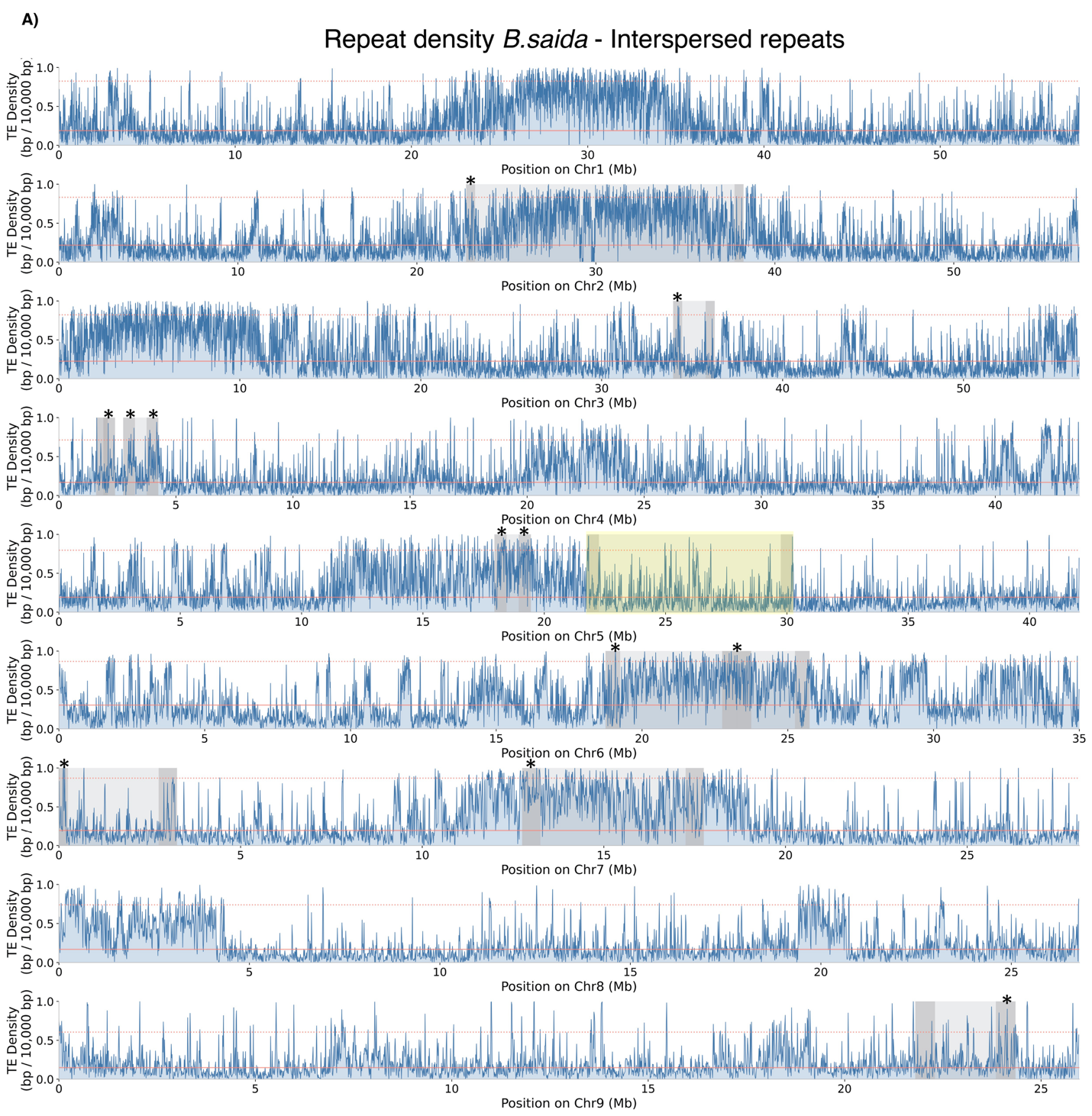
**

**Fig. S15.** Repeat density across chromosomes, with annotated inversions for polar cod. **A)** Density of interspersed repeats (blue) within non-overlapping sliding windows of 10,000 bp in chromosomes harboring inversions in polar cod. Chromosomes are shown along each row in Mb. Inversions are highlighted in light grey, and breakpoints (+/- 250,000 kb for better visualization) are colored in dark grey at the ends of each inversion. A large sex-determining region on chromosome 5 is marked in yellow [53]. Chromosomal median densities are shown as red lines and 95^th^ percentiles as red dotted lines. Breakpoints that display density peaks above the 95^th^ percentile are marked with an asterisk.

**
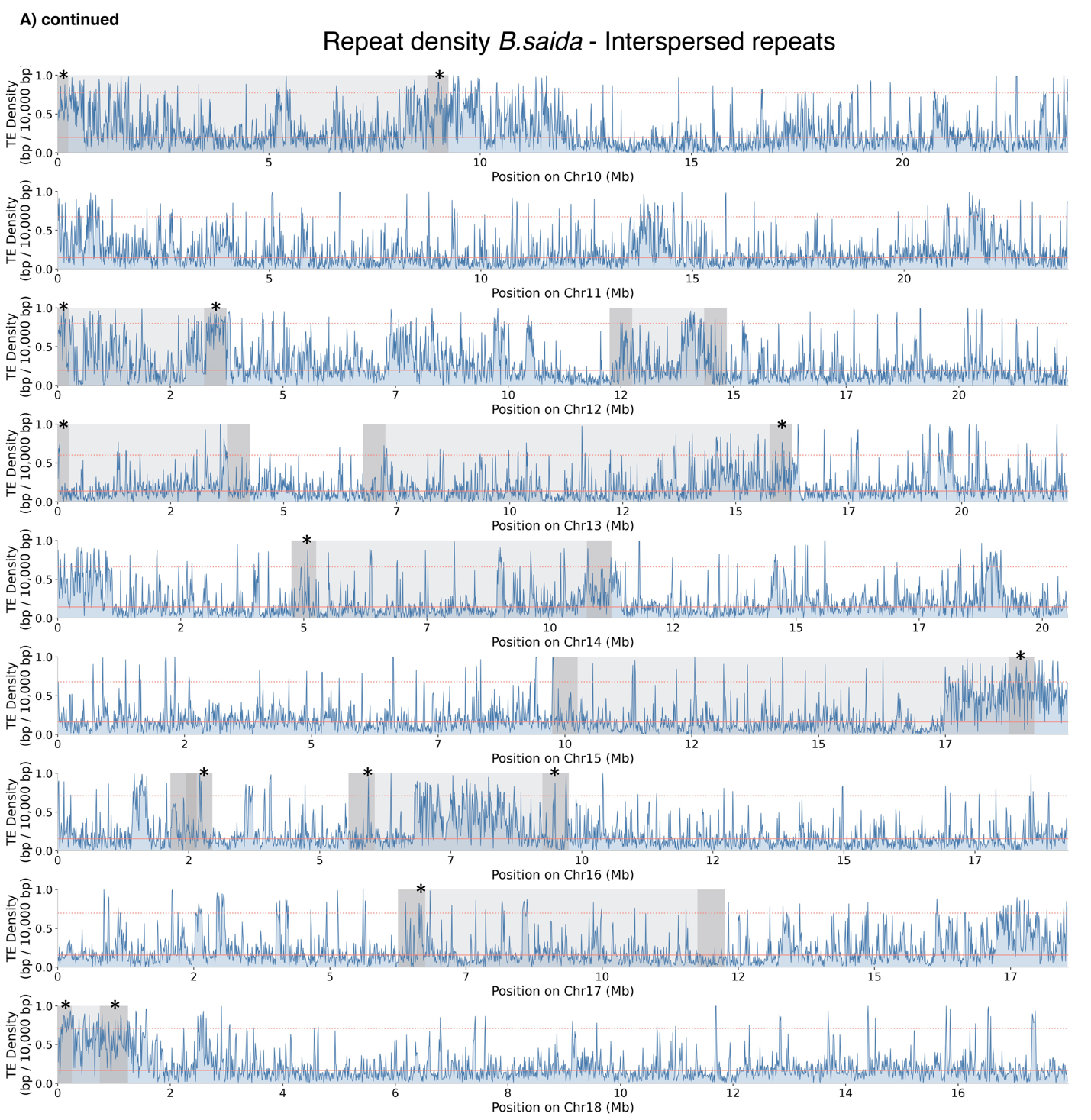
**

**Fig. S15 (continued).** Repeat density across chromosomes, with annotated inversions for polar cod. **A)** Density of interspersed repeats (blue) within non-overlapping sliding windows of 10,000 bp in chromosomes harboring inversions in polar cod. Chromosomes are shown along each row in Mb. Inversions are highlighted in light grey, and breakpoints (+/- 250,000 kb for better visualization) are colored in dark grey at the ends of each inversion. A large sex-determining region on chromosome 5 is marked in yellow [53]. Chromosomal median densities are shown as red lines and 95^th^ percentiles as red dotted lines. Breakpoints that display density peaks above the 95^th^ percentile are marked with an asterisk.

**
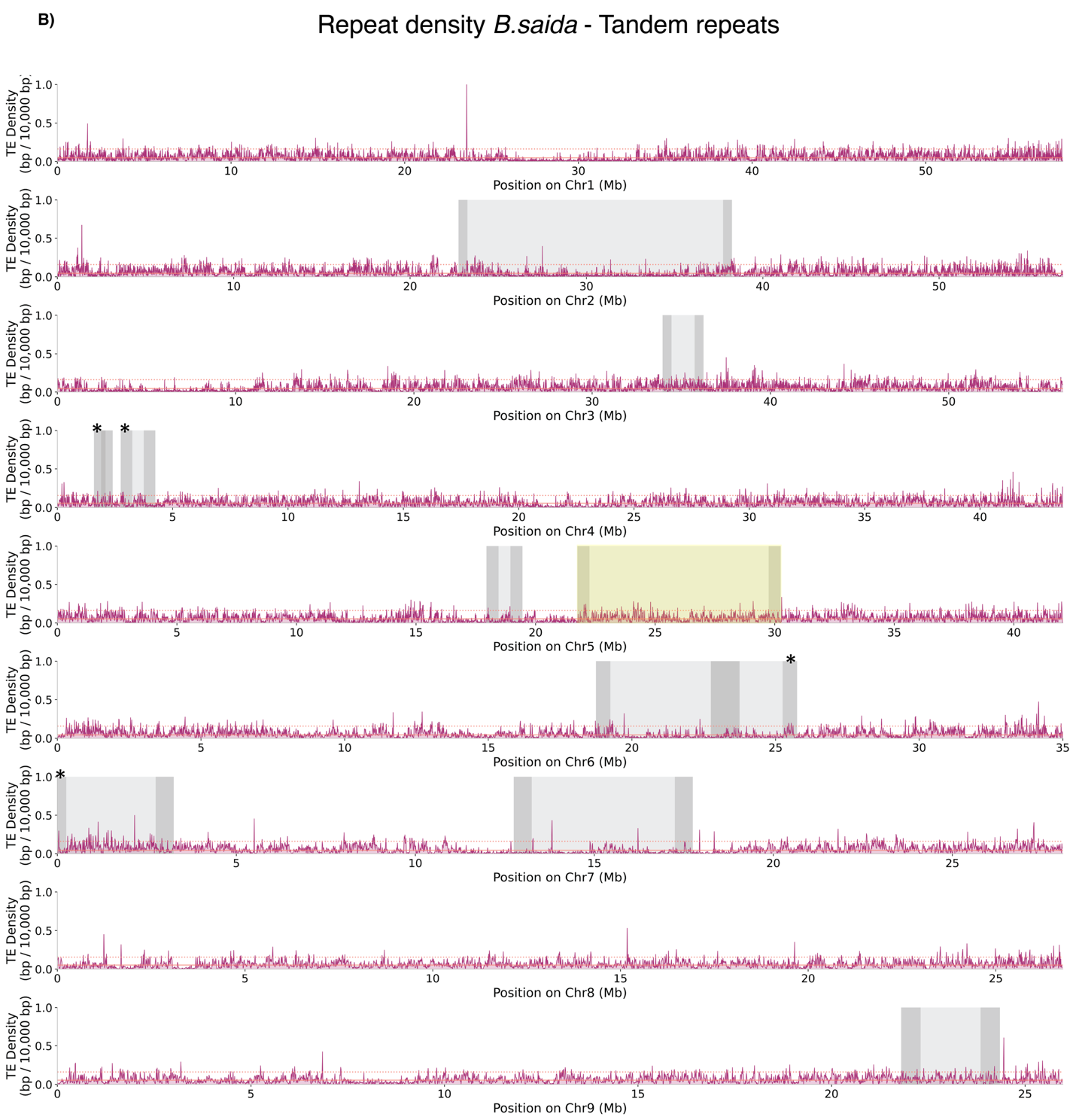
**

**Fig. S15 (continued).** Repeat density across chromosomes, with annotated inversions for polar cod. **B)** Density of simple repeats (red) within non-overlapping sliding windows of 10,000 bp in chromosomes harboring inversions in polar cod. Chromosomes are shown along each row in Mb. Inversions are highlighted in light grey, and breakpoints (+/- 250,000 kb for better visualization) are colored in dark grey at the ends of each inversion. A large sex-determining region on chromosome 5 is marked in yellow [53]. Chromosomal median densities are shown as red lines and 95^th^ percentiles as red dotted lines. Breakpoints that display density peaks above the 95^th^ percentile are marked with an asterisk.

**
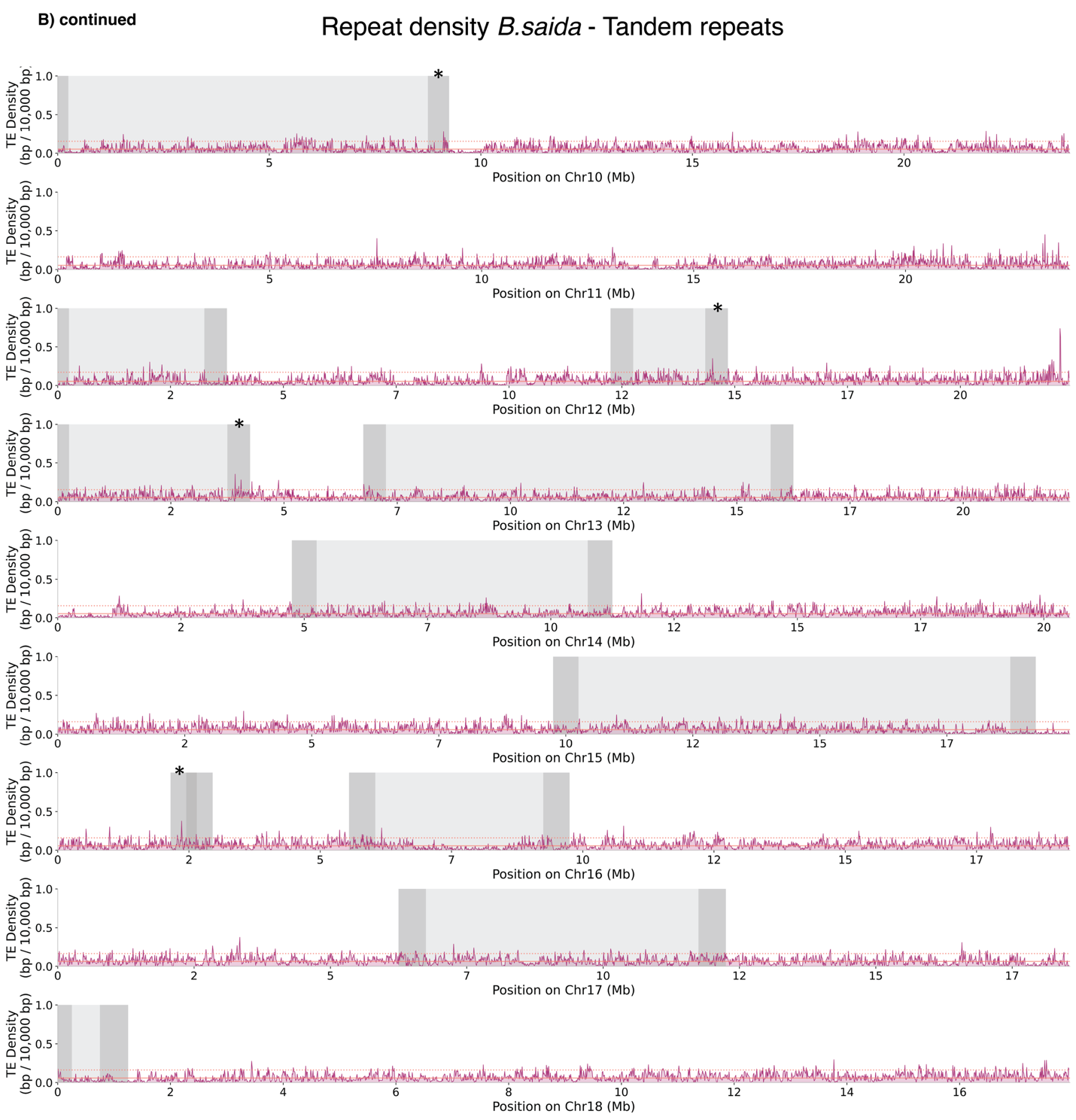
**

**Fig. S15 (continued).** Repeat density across chromosomes, with annotated inversions for polar cod. **B)** Density of simple repeats (red) within non-overlapping sliding windows of 10,000 bp in chromosomes harboring inversions in polar cod. Chromosomes are shown along each row in Mb. Inversions are highlighted in light grey, and breakpoints (+/- 250,000 kb for better visualization) are colored in dark grey at the ends of each inversion. A large sex-determining region on chromosome 5 is marked in yellow [53]. Chromosomal median densities are shown as red lines and 95^th^ percentiles as red dotted lines. Breakpoints that display density peaks above the 95^th^ percentile are marked with an asterisk.

**
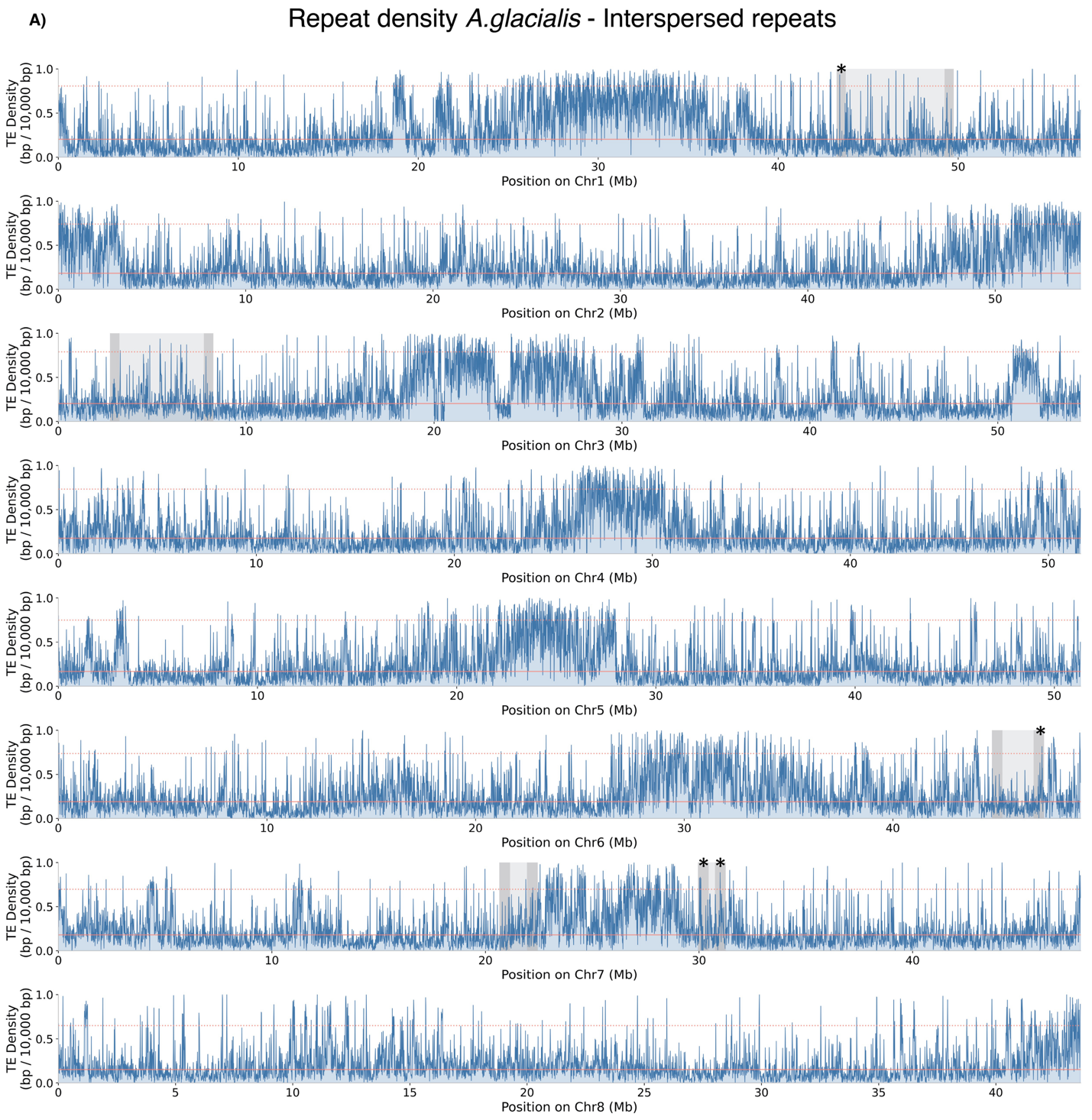
**

**Fig. S16.** Repeat density across chromosomes, with annotated inversions for Arctic cod. **A)** Density of interspersed repeats (blue) within non-overlapping sliding windows of 10,000 bp in chromosomes harboring inversions in polar cod. Chromosomes are shown along each row in Mb. Inversions are highlighted in light grey, and breakpoints (+/- 250,000 kb for better visualization) are colored in dark grey at the ends of each inversion. Chromosomal median densities are shown as red lines and 95^th^ percentiles as red dotted lines. Breakpoints that display density peaks above the 95^th^ percentile are marked with an asterisk.

**
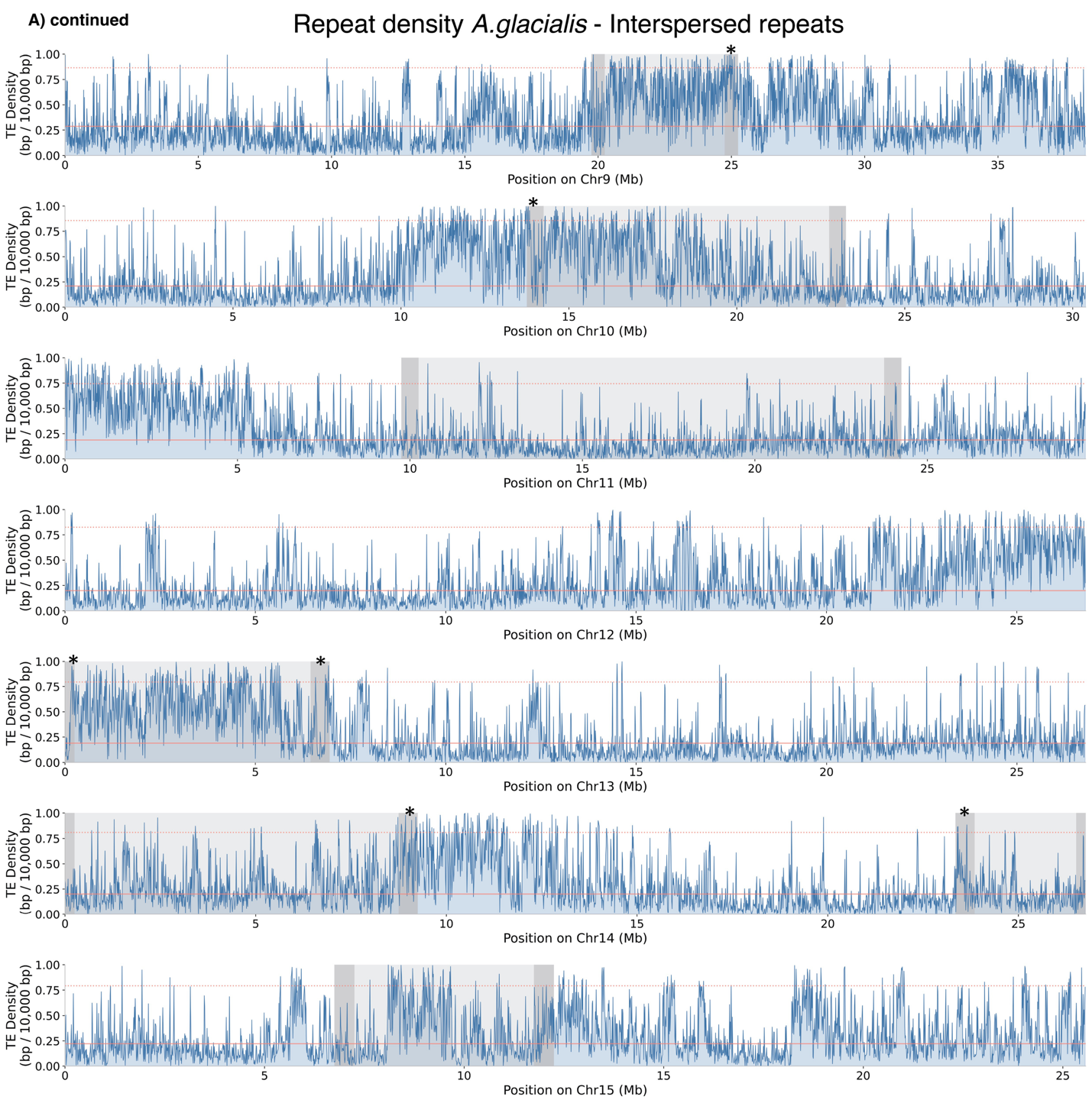
**

**Fig. S16 (continued).** Repeat density across chromosomes, with annotated inversions for Arctic cod. **A)** Density of interspersed repeats (blue) within non-overlapping sliding windows of 10,000 bp in chromosomes harboring inversions in polar cod. Chromosomes are shown along each row in Mb. Inversions are highlighted in light grey, and breakpoints (+/- 250,000 kb for better visualization) are colored in dark grey at the ends of each inversion. Chromosomal median densities are shown as red lines and 95^th^ percentiles as red dotted lines. Breakpoints that display density peaks above the 95^th^ percentile are marked with an asterisk.

**
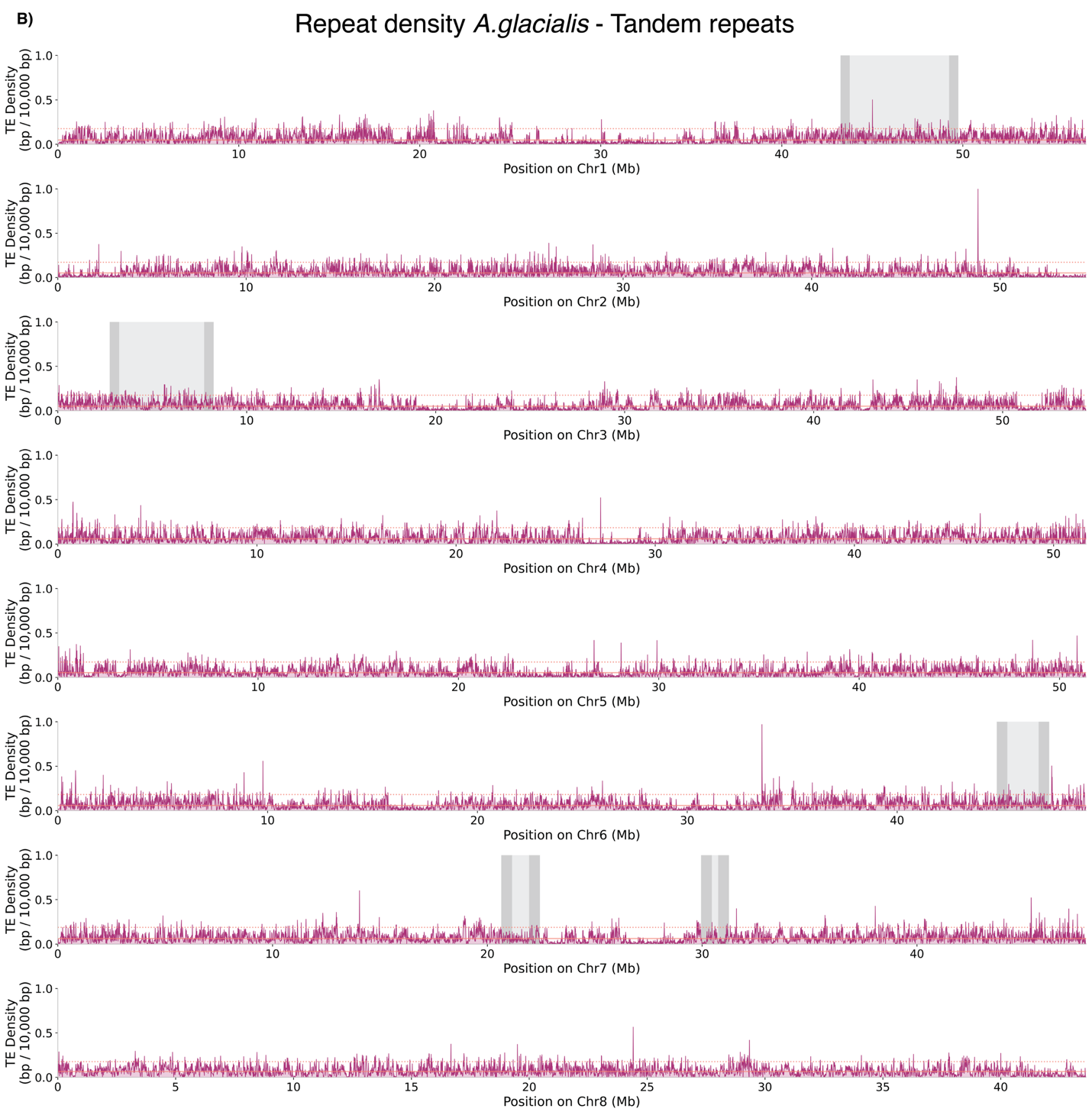
**

**Fig. S16 (continued).** Repeat density across chromosomes, with annotated inversions for Arctic cod. **B)** Density of simple repeats (red) within non-overlapping sliding windows of 10,000 bp in chromosomes harboring inversions in polar cod. Chromosomes are shown along each row in Mb. Inversions are highlighted in light grey, and breakpoints (+/- 250,000 kb for better visualization) are colored in dark grey at the ends of each inversion. Chromosomal median densities are shown as red lines and 95^th^ percentiles as red dotted lines. Breakpoints that display density peaks above the 95^th^ percentile are marked with an asterisk.

**
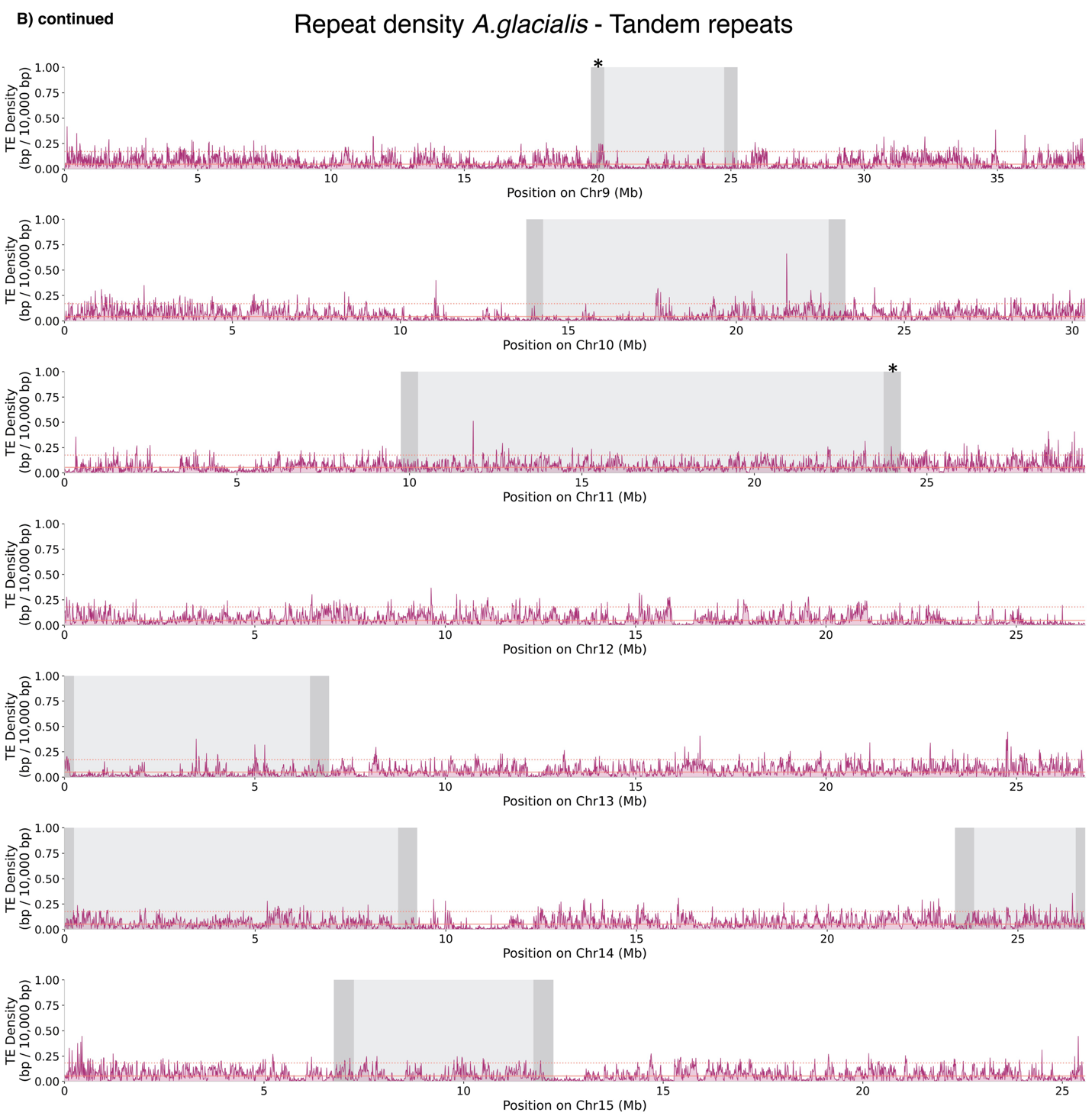
**

**Fig. S16 (continued).** Repeat density across chromosomes, with annotated inversions for Arctic cod. **B)** Density of simple repeats (red) within non-overlapping sliding windows of 10,000 bp in chromosomes harboring inversions in polar cod. Chromosomes are shown along each row in Mb. Inversions are highlighted in light grey, and breakpoints (+/- 250,000 kb for better visualization) are colored in dark grey at the ends of each inversion. Chromosomal median densities are shown as red lines and 95^th^ percentiles as red dotted lines. Breakpoints that display density peaks above the 95^th^ percentile are marked with an asterisk.


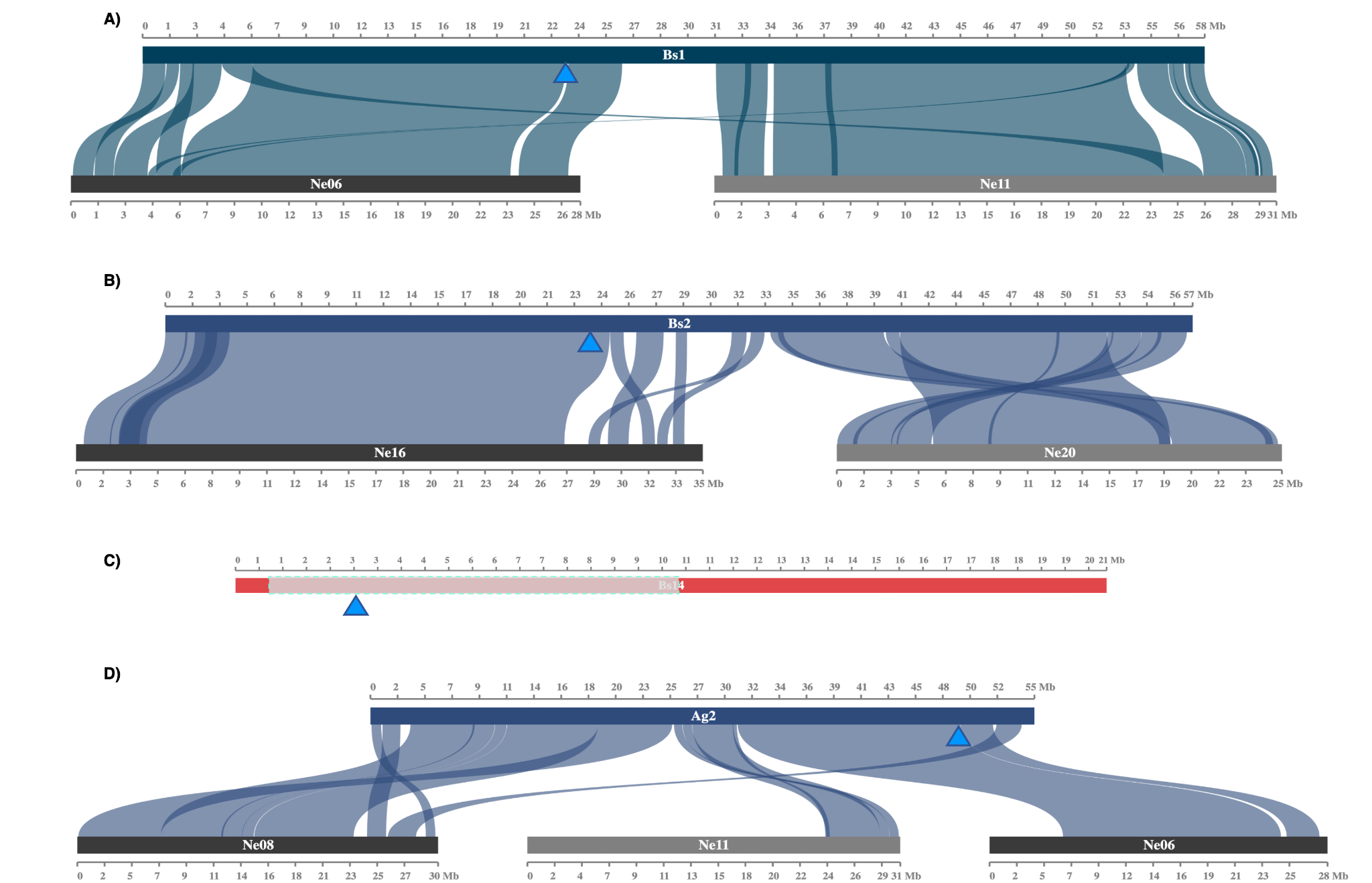


**Fig. S17. A-C)** chromosomal location of the *afgp* gene cluster in polar cod, where A) are the locations of cluster I and III on the fused chromosomes and C) cluster II on the chromosome harboring an intraspecies inversion. The inversion is marked with a turquoise box. **D)** Genomic location of *afgp* gene cluster I in Arctic cod. Location of *afgp* gene clusters is indicated with a blue triangle. Chromosomal synteny compared to Atlantic cod is shown for the fused chromosomes in both polar cod and Arctic cod. Genomic locations in the PacBio genome assemblies were identified by dotplot to the ONT draft assemblies.


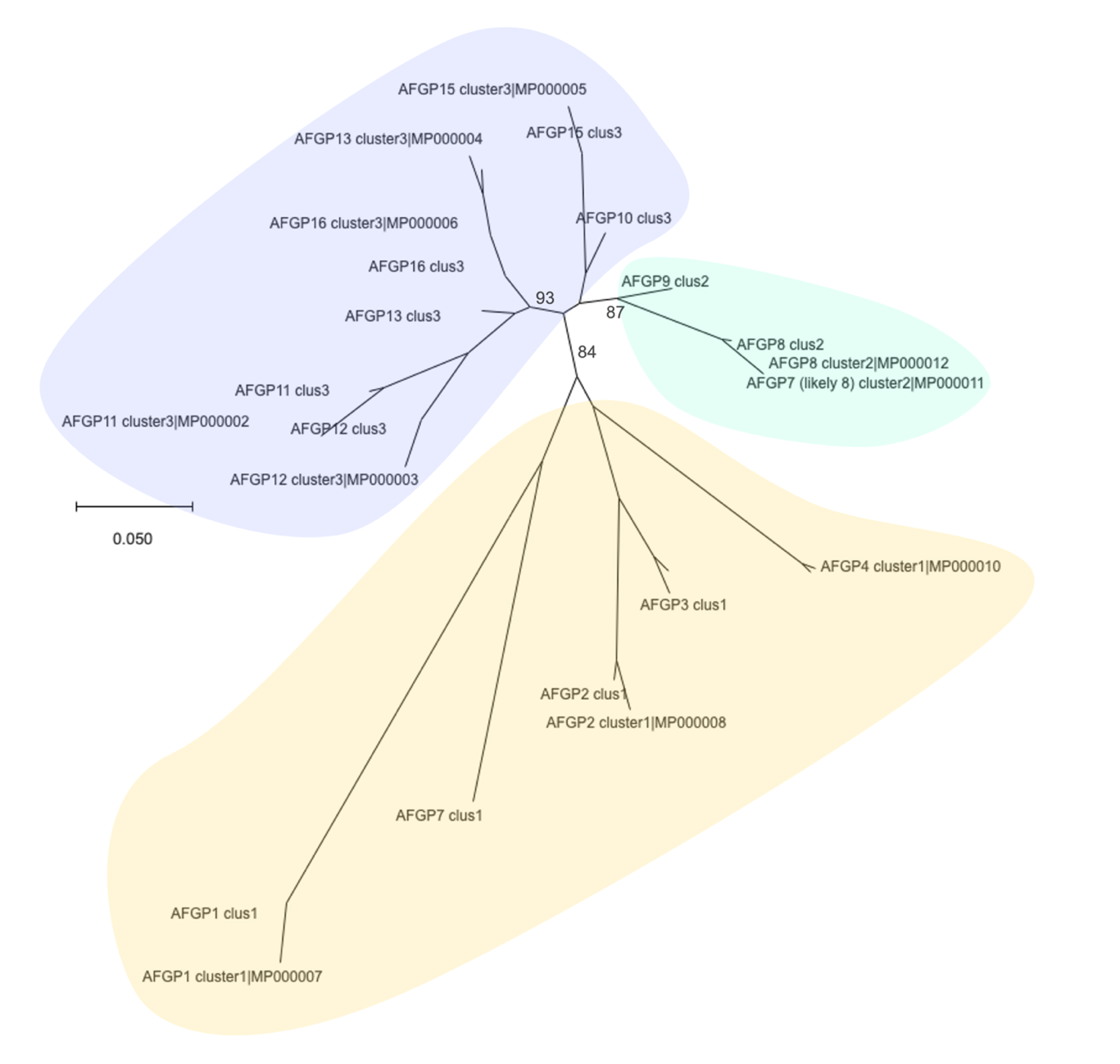

**Fig. S18.** Unrooted Maximum Likelihood (ML) tree using the Tamura-Nei (1993) model and 100 standard bootstrap replicates using the coding sequence of all *afgp* genes identified in polar cod in the present study, as well as the previously identified *afgp* genes [76]. Branch support is shown for the primary branching. Genes marked *MP000000x* (naming from identification by miniprot [142]) are genes extracted from the ONT draft assembly from the present study. The ML tree of the polar cod *afgp* genes, where they group according to their respective gene clusters, suggesting a closer sequence similarity among genes within each cluster, compared to between clusters. Colors indicate *afgp* gene clusters; yellow: cluster I, green: cluster II, and purple: cluster III.

**Fig. S19.** This shows the number of **A)** expanded and **B)** contracted gene families throughout the investigated species estimated by CAFE5 [154]. The gene families were found by OrthoFinder [153], and the species tree was also calculated by OrthoFinder.


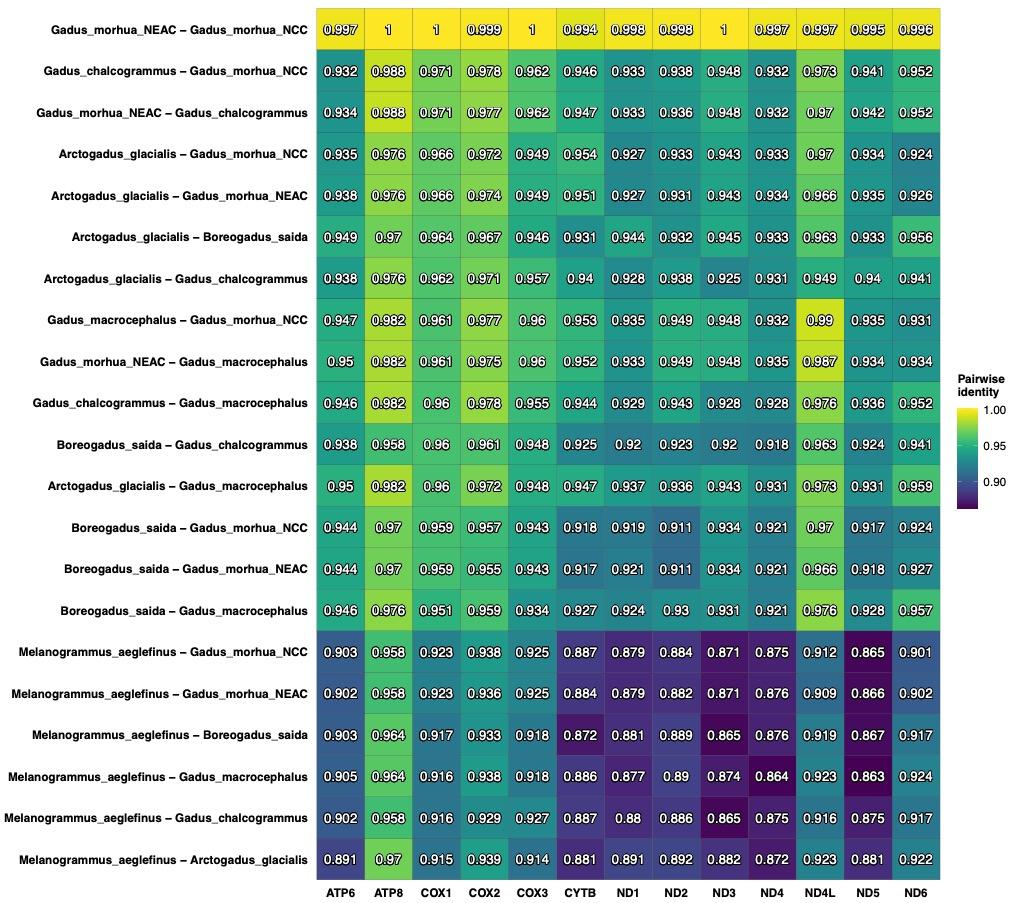


**Fig. S20.** Pairwise identity calculated with PhyKIT [170] for each mitochondrial gene between Arctic cod, polar cod, NEAC, NCC, and Atlantic haddock.


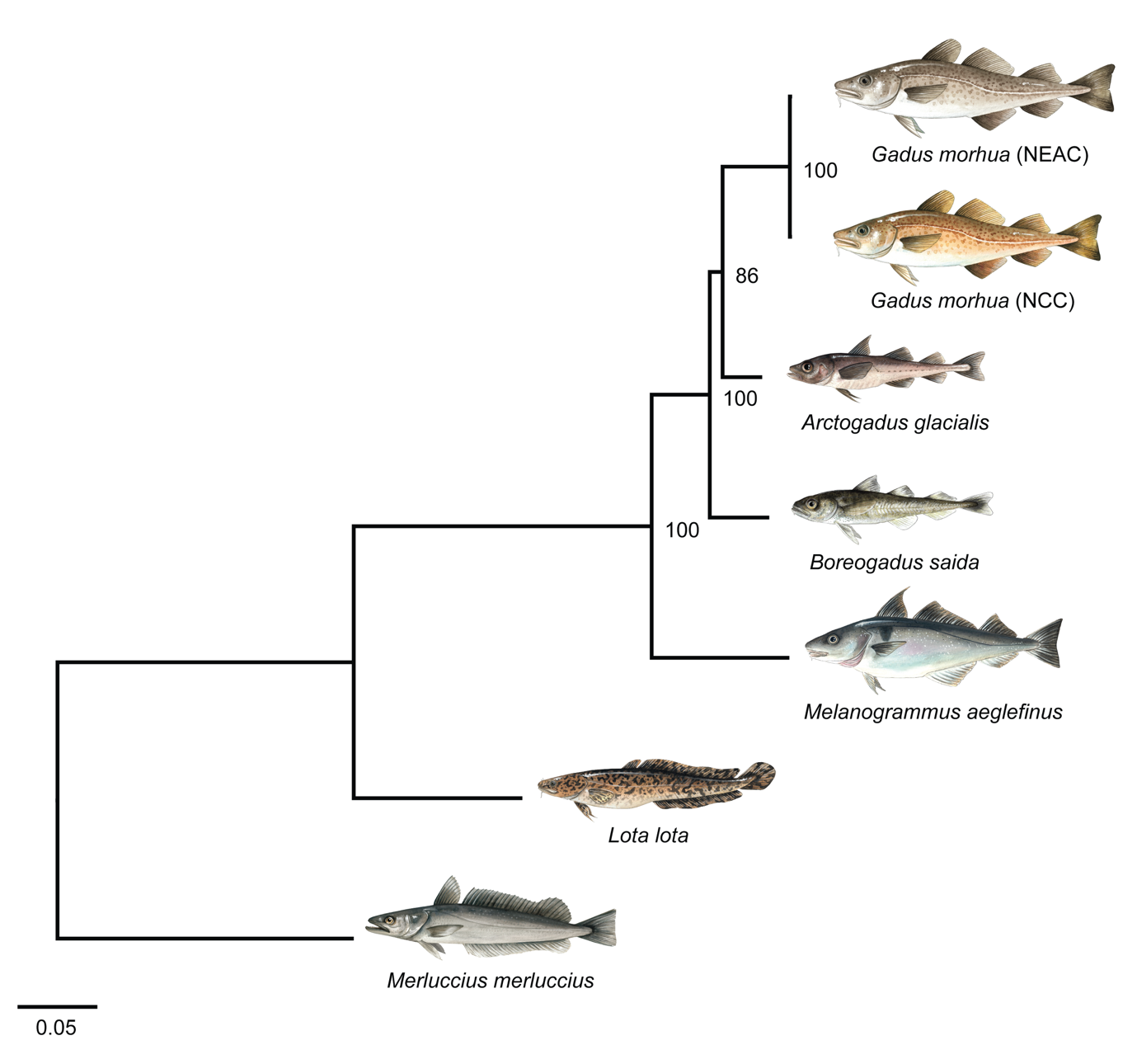


**Fig. S21.** Mitochondrial phylogeny of taxa with sequenced genomes in this study, except for European hake, which was accessed from NCBI accession: NC_007396.1. ML phylogeny of the focal species using a concatenation approach of all 13 protein-coding genes with maximum likelihood tree inference in IQ-Tree2 [59]. Branch length is given in substitutions per site, and bootstrap support is given. All fish illustrations are by Alexandra Viertler.


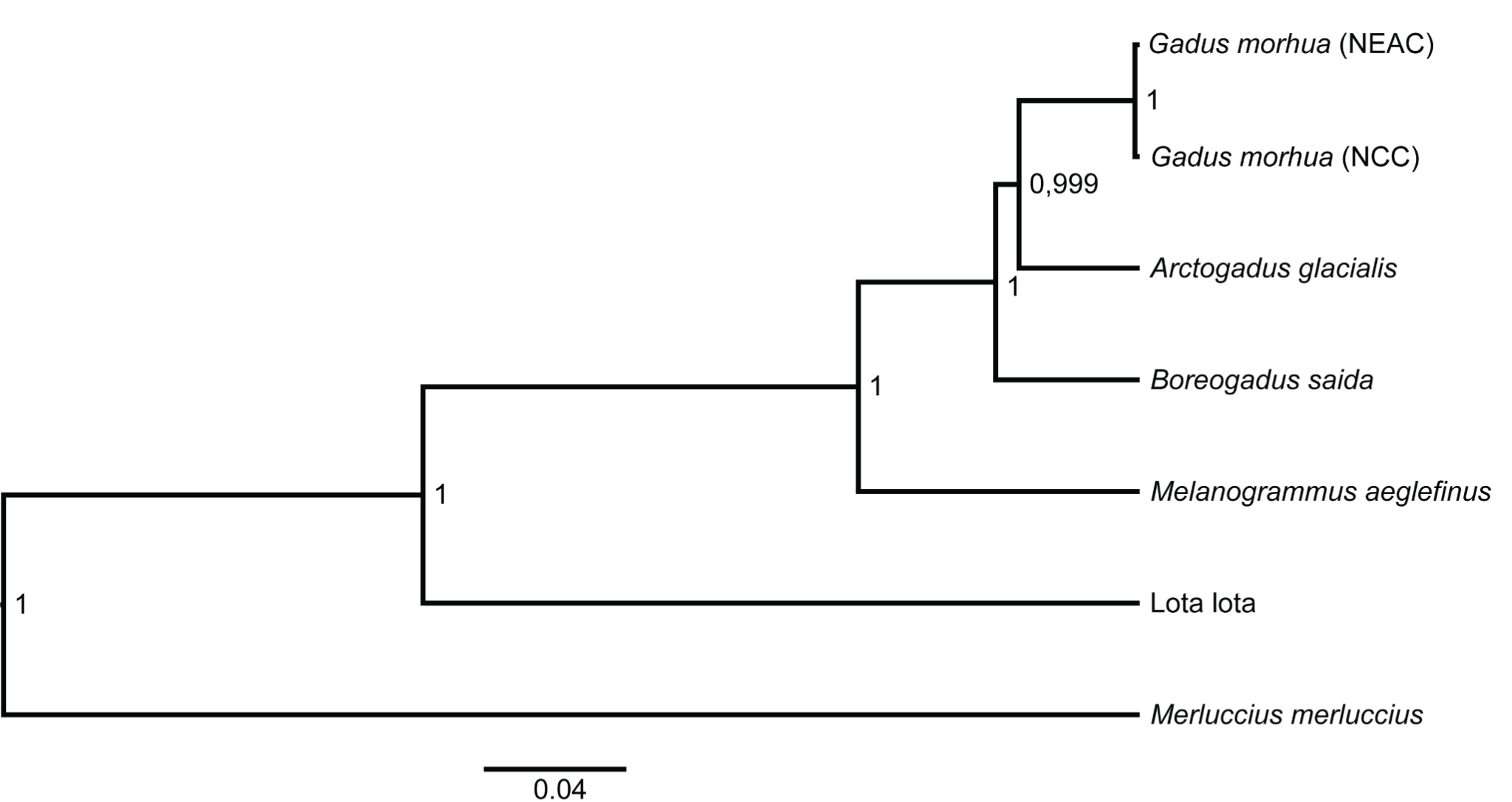


**Fig. S22.** Bayesian phylogenetic tree of taxa with sequenced genomes included in this study, except for European hake, which was accessed from NCBI accession: NC_007396.1. Bayesian phylogeny of the focal species using a concatenation approach of all 13 protein-coding genes inferred in BEAST2 [194]. Branch length is given in substitution rate, and support given as posterior probabilities.

**
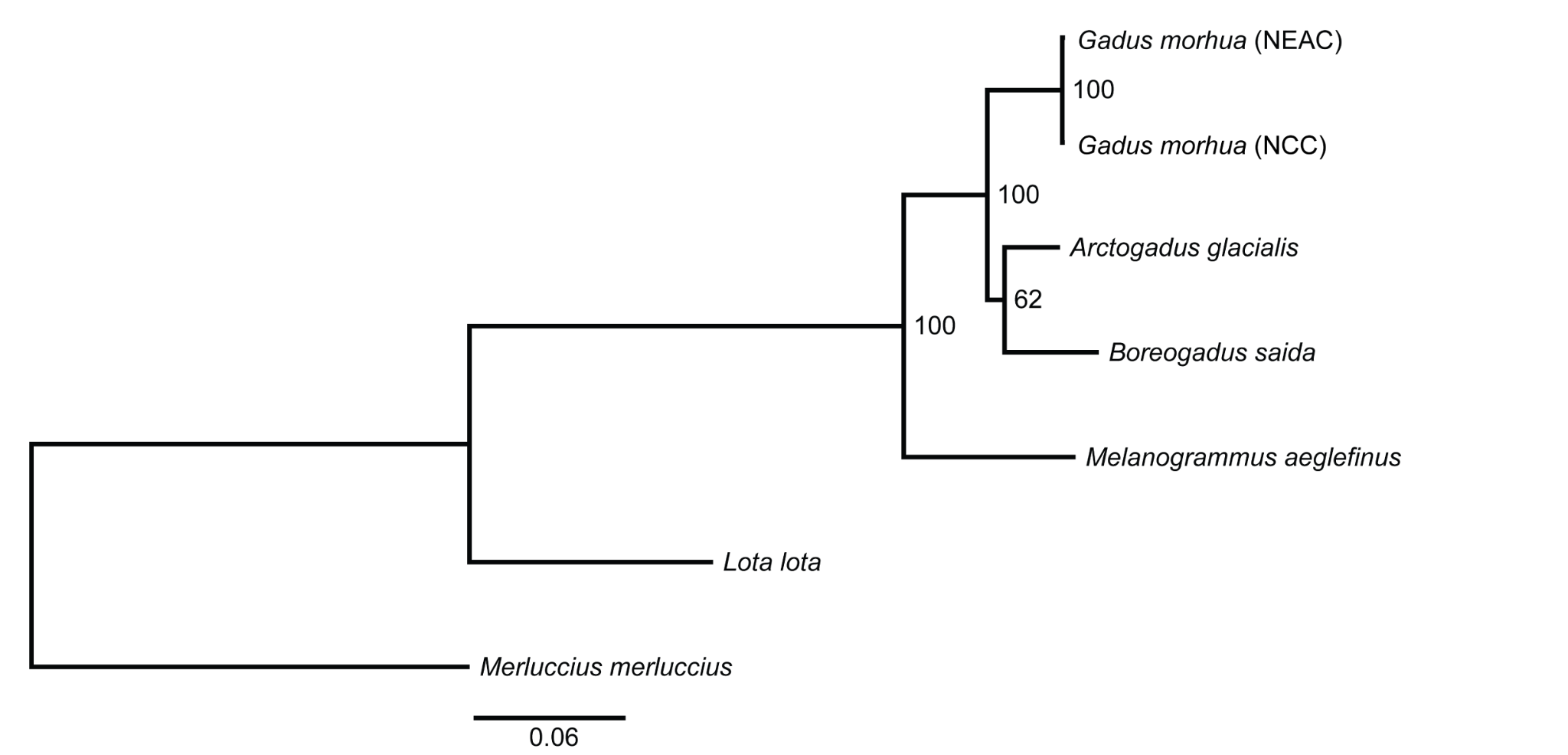
 Fig. S23.** Maximum likelihood phylogenetic tree of complete mitogenomes inferred in IQ-Tree2 [59]. Bootstrap support shown and branch length given as substitution rate.

**
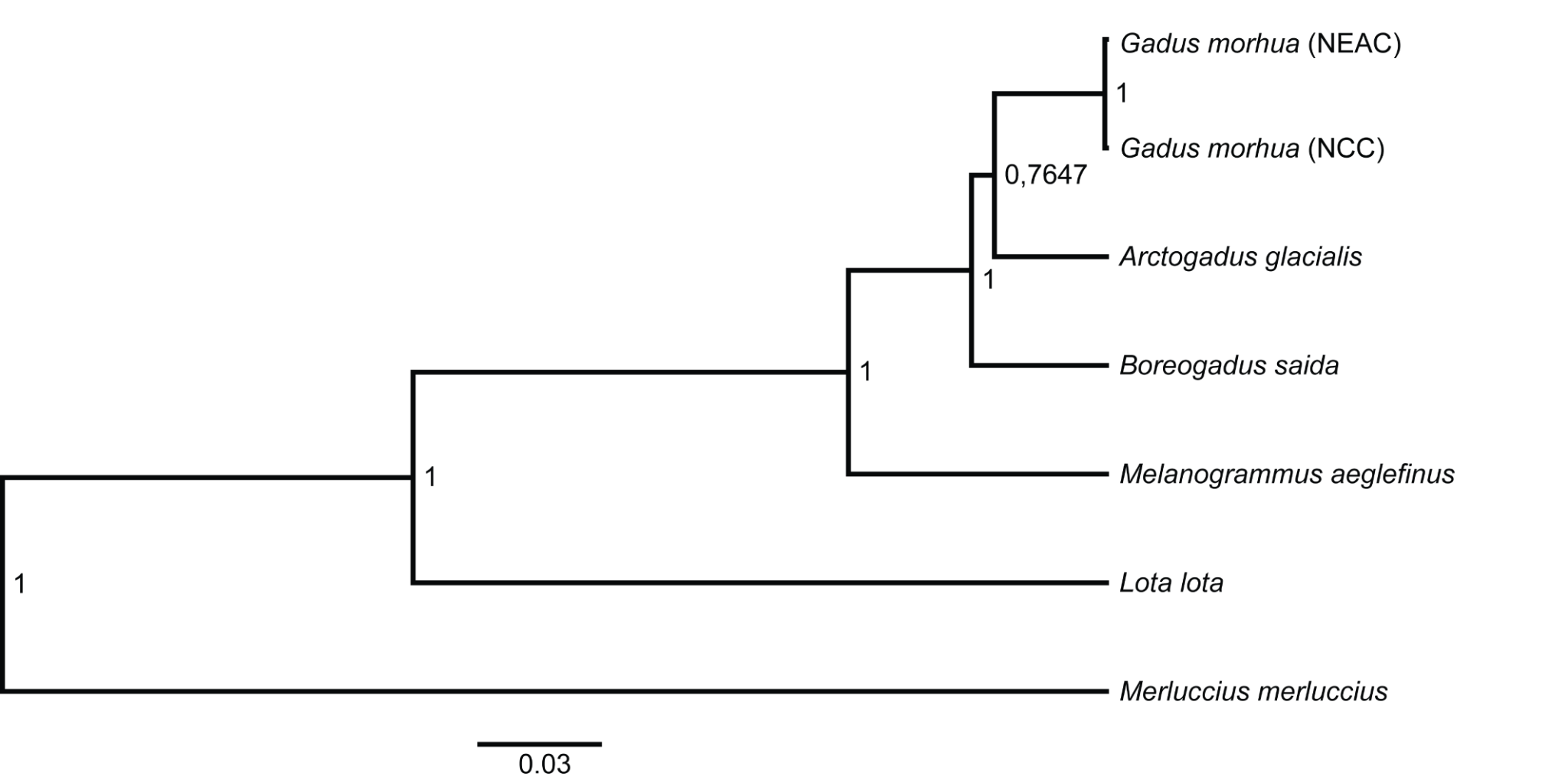
Fig. S24.** Bayesian phylogenetic tree of complete mitogenomes inferred in BEAST2 [194], support values given as posterior probabilities, and branch length given as substitution rate.

**Fig. S25.** A phylogenetic linear regression, applying the mitochondrial ML tree between chromosomal number (n) and **A)** preferred ocean temperature (estimated as mean °C) and **B)** northerly distribution (estimated by southernmost latitudinal range limit) for representing 13 codfish species (see Methods for more details). Abbreviations are given as: Ag; Arctic cod (*Arctogadus glacialis*), Bs; Polar cod (*Boreogadus saida*), Ll: Burbot (*Lota lota*), Eh: European hake (*Merluccius merluccius*), Mp: Blue whiting (*Micromesistius poutassou*), Tm: Poor cod (*Trisopterus minutus*), Eg: Saffron cod (*Eleginus gracilis*), Pv: Saithe (*Pollachius virens*), Pp: Atlantic pollock (*Pollachius pollachius*), Ma: Atlantic haddock (*Melanogrammus aeglefinus*), Gmo: Atlantic cod (*Gadus morhua*), Gc: Alaska pollock (*Gadus chalcogrammus*), Gma: Pacific cod (*Gadus macrocephalus*).

A

C

B


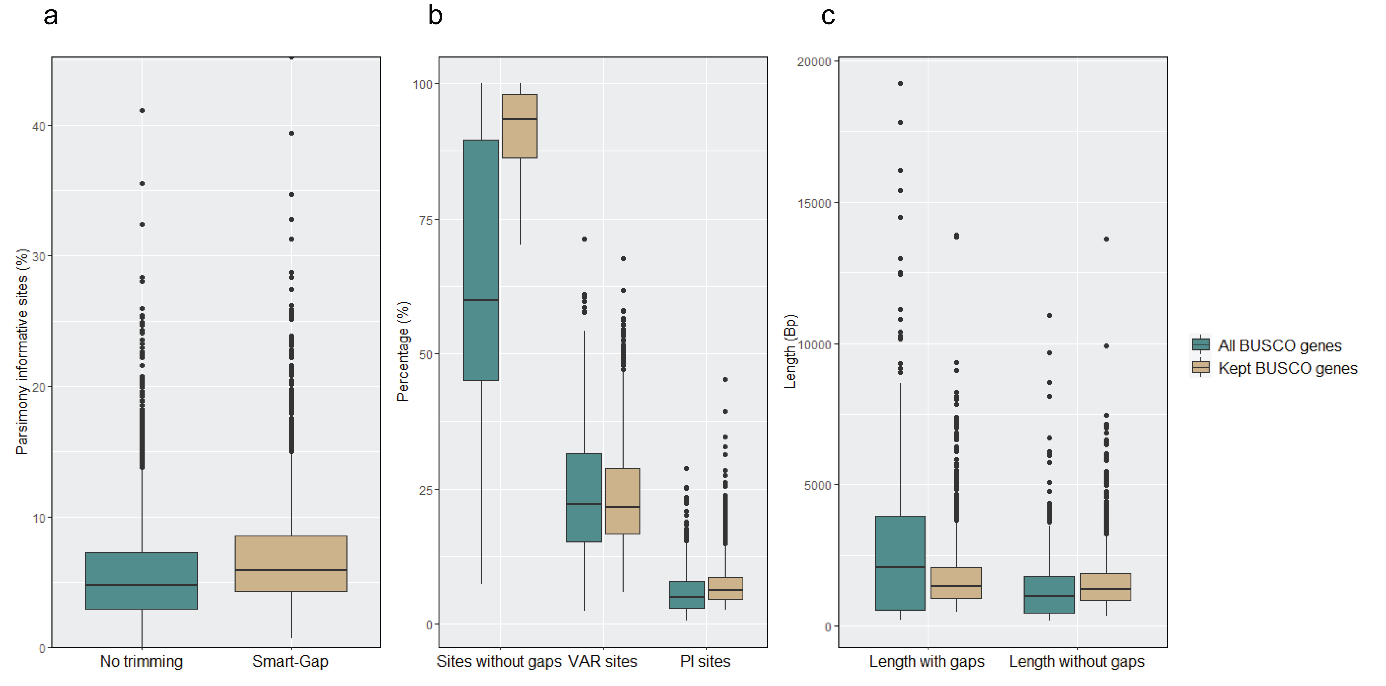


**Fig. S26.** Phykit [170] stats represented as boxplot for **A)** percentage of parsimony informative (PI) sites under no trimming versus smart-gap trimming with ClipKIT [174], **B)** sites without gaps, variable (VAR) sites and PI sites after removing BUSCO genes with more than 30% sites containing gaps, less than 5% VAR sites and 2.5% PI sites, **C)** length of BUSCO genes with and without gaps after filtering on minimum length of 500 Bp. Blue box plots show all BUSCO genes, and brown box plots show BUSCO genes after filtering.


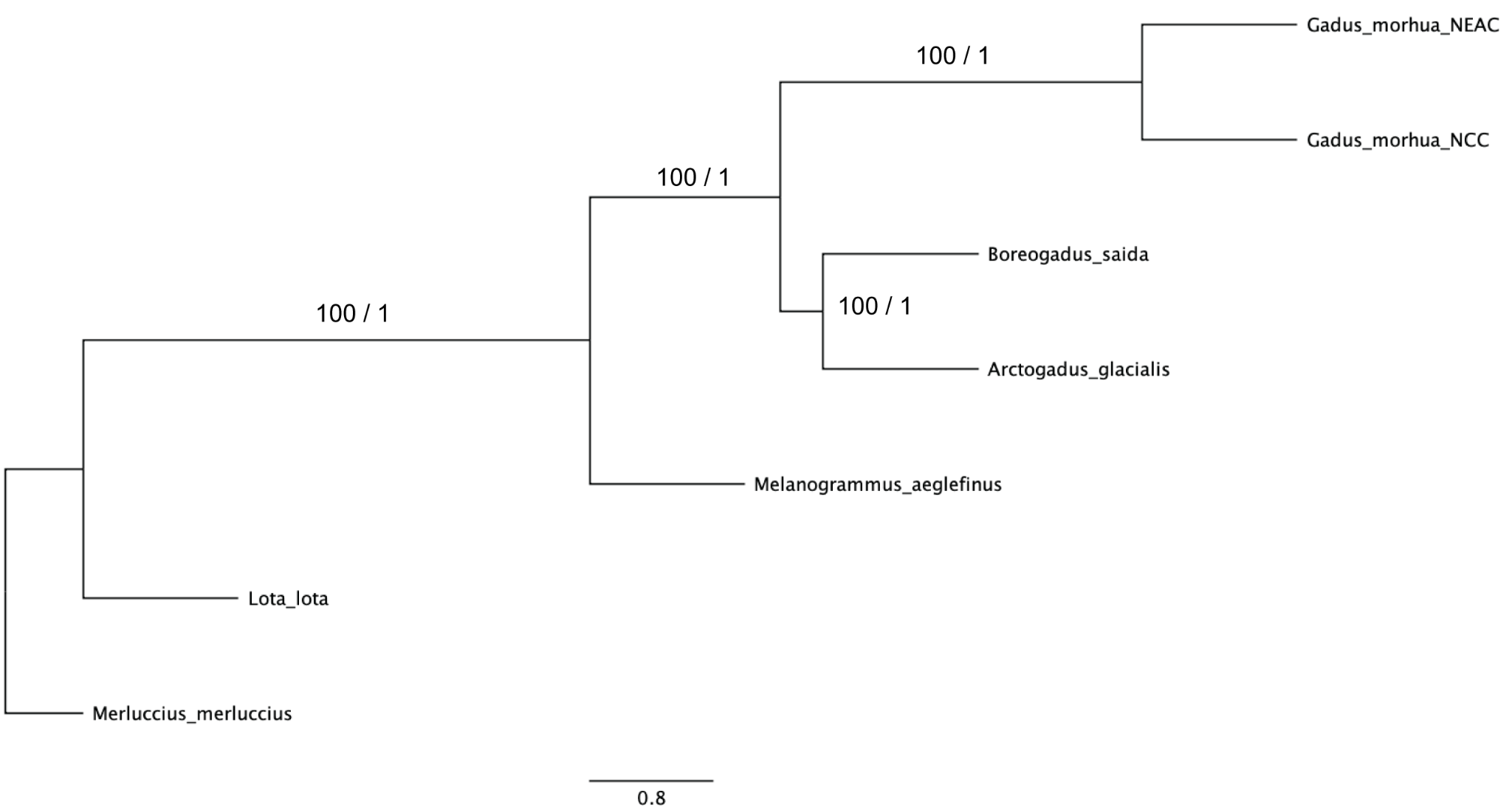


**Fig. S27.** Species tree inferred using Astral-III [65] that corresponds to the tree in Fig. 7. Support values given as bootstrap / posterior probability, and branch lengths given as coalescent units.


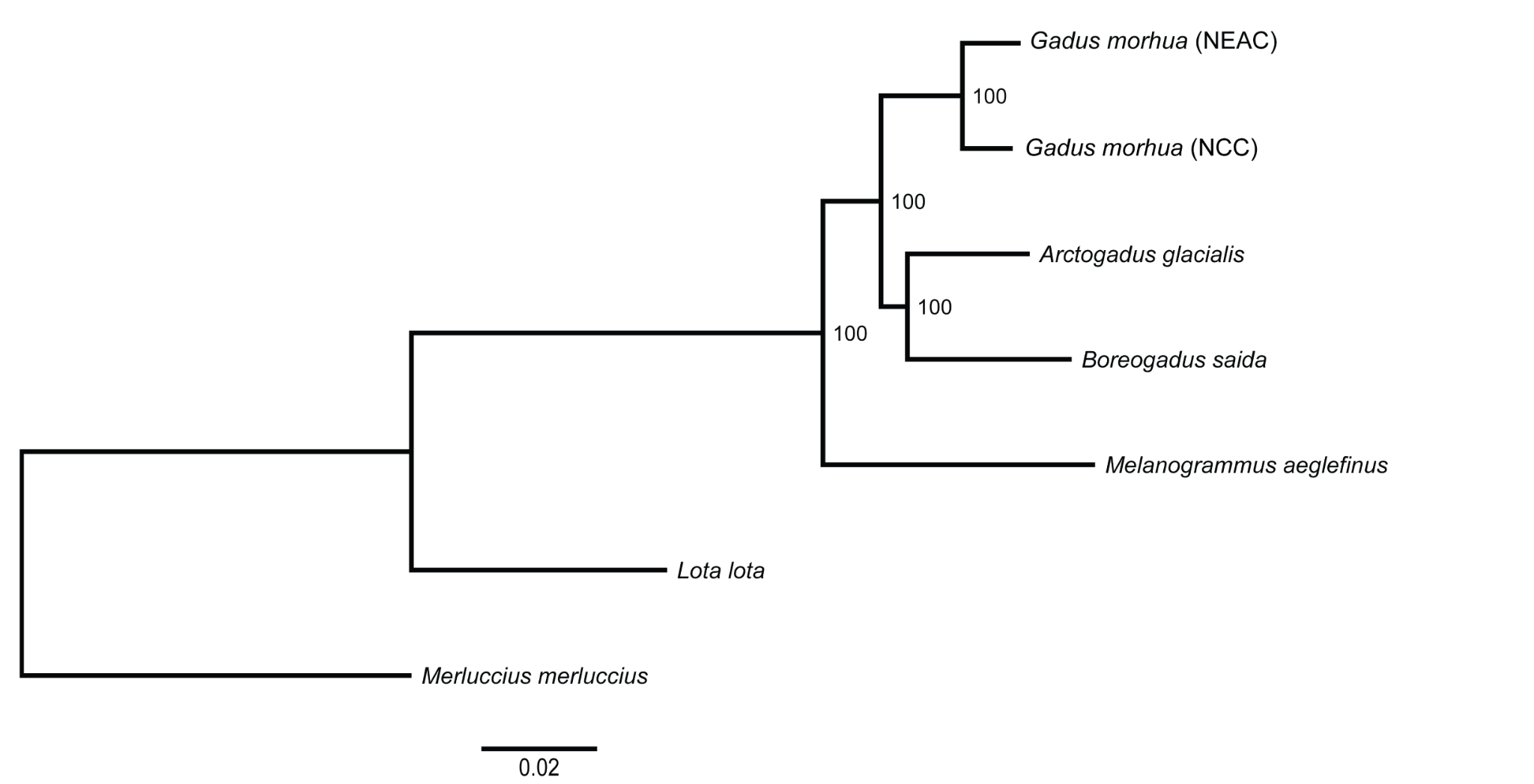


**Fig. S28.** Maximum likelihood tree inferred in IQ-Tree2 using 1939 BUSCO genes concatenated into a supermatrix. Bootstrap supports are shown, and branch lengths are given in nucleotide substitutions per site.


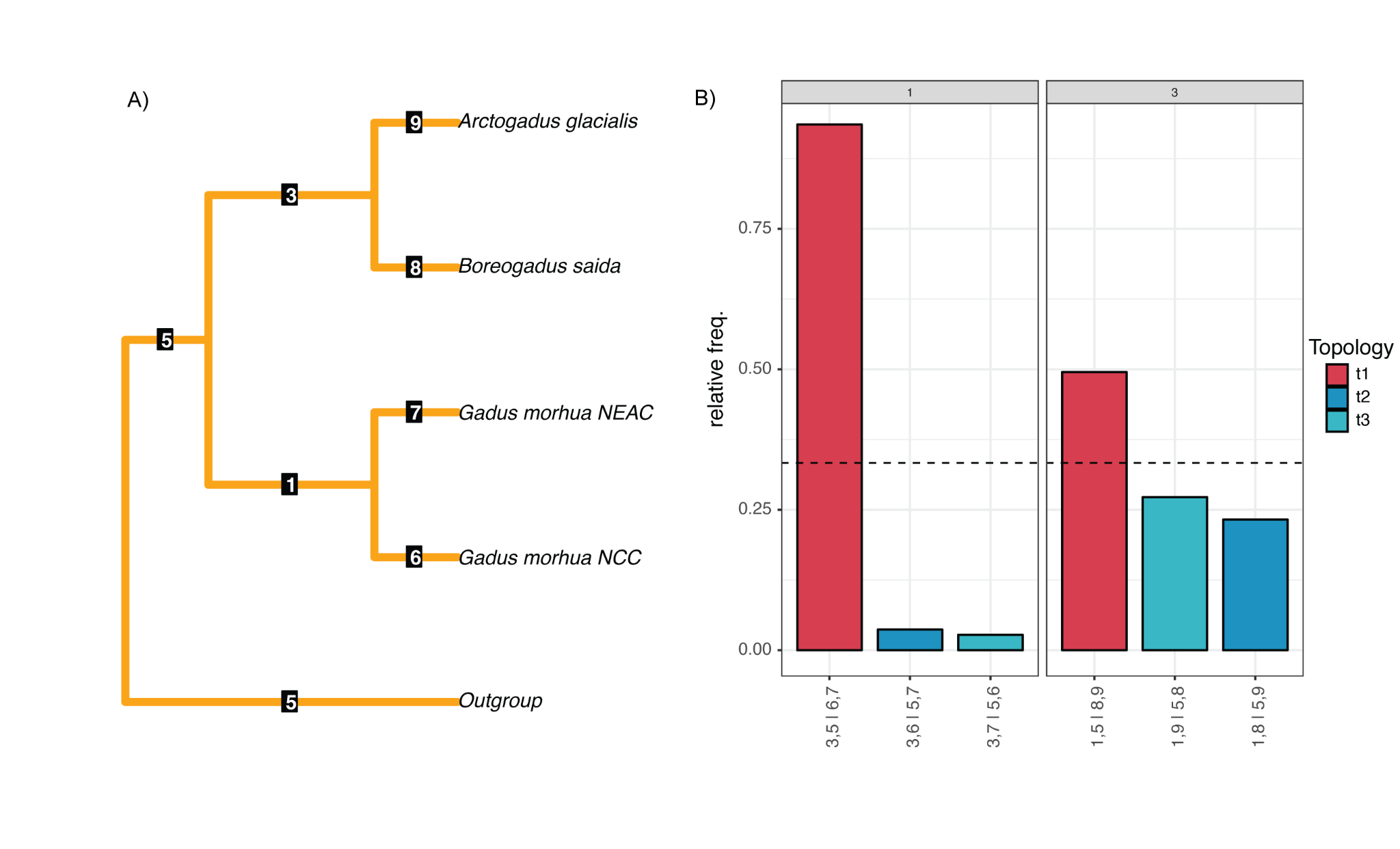


**Fig. S29.** The output of quartet frequencies from DiscoVista [79] with *Merluccius merluccius, Lota lota,* and *Melanogrammus aeglefinus* specified as outgroups. **A)** Species tree with internal branch numbers given. **B)** Quartet frequencies are given for each possible topology of the internal branches of interest (1 and 3). Possible topologies are given on the x-axis.

**Fig. S30.** Map showing sampling location for the six codfish species that were genomes sequenced in the present study. Illustrations made by Alexandra Viertler.


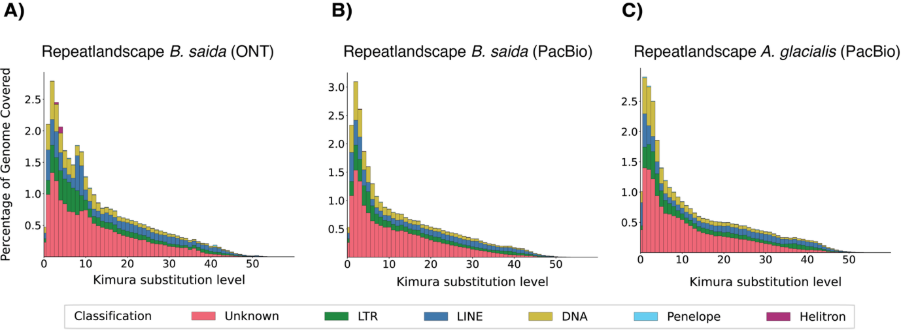


**Fig. S31.** Repeat landscapes of Arctic codfishes. Genomic coverage of annotated TEs (%) is plotted against their divergence from consensus sequences (Kimura substitution level) for **A)** polar cod (ONT assembly), **B)** polar cod (PacBio HiFi assembly), and **C)** Arctic cod (PacBio HiFi assembly). TEs are colored according to the legend.


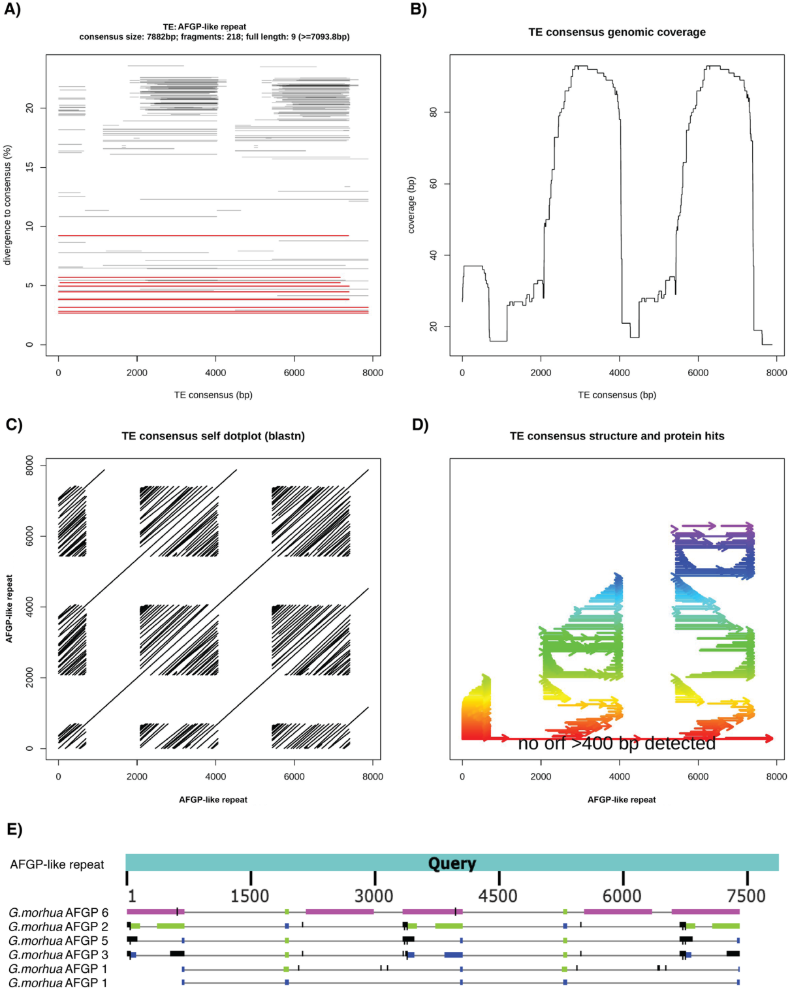


**Fig. S32.** Sequence features of *afgp*-like repeat. Fig. A-D shows output from TE-Aid [148]. **A)** Number of genomic hits across the length of the TE consensus (x-axis) plotted against its divergence to consensus (y-axis). Full-length hits are colored in red. **B)** TE consensus genomic coverage, where coverage (bp) is plotted across the consensus length (bp). **C)** Self-dot-plot of the *afgp*-like repeat, revealing internal repeats across three consecutive repeating units**. D)** Structure and protein hits of the *afgp*-like repeat sequence, showing internal repeats (colors indicate repeat sequence similarity) and that the sequence has no open reading frames (ORFs). **E)** Output from BLASTN when using the *afgp*-like repeat as query against the *afgp* gene sequences identified in Baalsrud et al. [46].

**References**

46. Baalsrud HT, Tørresen OK, Solbakken MH, Salzburger W, Hanel R, Jakobsen KS, et al. De novo gene evolution of antifreeze glycoproteins in codfishes revealed by whole genome sequence data. Mol Biol Evol. 2018;35:593–606. https://doi.org/10.1093/molbev/msx311

53. Hoff SNK, Maurstad MF, Moan AL, Ravinet M, Pampoulie C, Vieweg I, et al. Genomic rearrangements drive population divergence in a keystone Arctic species with high gene flow. bioRxiv. 2024; https://doi.org/10.1101/2024.06.28.597535

59. Minh BQ, Schmidt HA, Chernomor O, Schrempf D, Woodhams MD, von Haeseler A, et al. IQ-TREE 2: New models and efficient methods for phylogenetic inference in the genomic era. Mol Biol Evol. 2020;37:1530–4. https://doi.org/10.1093/molbev/msaa015

65. Zhang C, Rabiee M, Sayyari E, Mirarab S. ASTRAL-III: polynomial time species tree reconstruction from partially resolved gene trees. BMC Bioinformatics. 2018;19:153. https://doi.org/10.1186/s12859-018-2129-y

74. Korunes KL, Samuk K. pixy: Unbiased estimation of nucleotide diversity and divergence in the presence of missing data. Mol Ecol Resour. 2021;21:1359–68. https://doi.org/10.1111/1755-0998.13326

76. Zhuang X, Yang C, Murphy KR, Cheng C-HC. Molecular mechanism and history of non-sense to sense evolution of antifreeze glycoprotein gene in northern gadids. Proc Natl Acad Sci U S A. 2019;116:4400–5. https://doi.org/10.1073/pnas.1817138116

79. Sayyari E, Whitfield JB, Mirarab S. DiscoVista: Interpretable visualizations of gene tree discordance. Mol Phylogenet Evol. 2018;122:110–5. https://doi.org/10.1016/j.ympev.2018.01.019

142. Li H. Protein-to-genome alignment with miniprot. Bioinformatics. 2023;39:btad014. https://doi.org/10.1093/bioinformatics/btad014

148. Goubert C, Craig RJ, Bilat AF, Peona V, Vogan AA, Protasio AV. A beginner’s guide to manual curation of transposable elements. Mob DNA. 2022;13:7. https://doi.org/10.1186/s13100-021-00259-7

153. Emms DM, Kelly S. OrthoFinder: phylogenetic orthology inference for comparative genomics. Genome Biol. 2019;20:238. https://doi.org/10.1186/s13059-019-1832-y

154. Mendes FK, Vanderpool D, Fulton B, Hahn MW. CAFE 5 models variation in evolutionary rates among gene families. Bioinformatics. 2021;36:5516–8. https://doi.org/10.1093/bioinformatics/btaa1022

170. Steenwyk JL, Buida TJ III, Labella AL, Li Y, Shen X-X, Rokas A. PhyKIT: a broadly applicable UNIX shell toolkit for processing and analyzing phylogenomic data. Bioinformatics. 2021;37:2325–31. https://doi.org/10.1093/bioinformatics/btab096

174. Steenwyk JL, Iii TJB, Li Y, Shen X-X, Rokas A. ClipKIT: A multiple sequence alignment trimming software for accurate phylogenomic inference. PLOS Biol. 2020;18:e3001007. https://doi.org/10.1371/journal.pbio.3001007

178. Thorvaldsdóttir H, Robinson JT, Mesirov JP. Integrative Genomics Viewer (IGV): high-performance genomics data visualization and exploration. Brief Bioinform. 2013;14:178–92. https://doi.org/10.1093/bib/bbs017

194. Bouckaert R, Vaughan TG, Barido-Sottani J, Duchêne S, Fourment M, Gavryushkina A, et al. BEAST 2.5: An advanced software platform for Bayesian evolutionary analysis. PLOS Comput Biol. 2019;15:e1006650. https://doi.org/10.1371/journal.pcbi.1006650
