# Supplementary material for: Rapid genome modifications including chromosomal fusions and large-scale inversions are key features in Arctic codfish species
Source: Genome Biol. 2026 Feb 16;27:100. doi: 10.1186/s13059-026-03975-6 (PMC13011446; doi:10.1186/s13059-026-03975-6)
Supplement: Supplementary file 3 — Additional file 3: Supplementary note 1. Description of genome assembly of burbot, and long-read mitogenomes for six codfishes [60, 61, 134, 135, 195–198]. [file 13059_2026_3975_MOESM3_ESM.docx]

**Supplementary note 1**

**The genome assembly of burbot**

The chromosome-level genome assemblies for the six codfishes generated corresponded to the numbers of chromosomes reported from cytogenetic studies (see Additional file 1, Table S1; Additional file 2, Fig. S1), except for burbot, where we obtained a total of n=23 haploid chromosomes vs. n=24 haploid chromosomes reported by two different cytogenetic studies [60,61]. We cannot rule out that super-scaffolds have erroneously been connected, which should have been split in the burbot assembly, but our Hi-C contact map did not indicate any super-scaffolds that should have been further split. Here we could also mention that Moreover, two different chromosome-level burbot assemblies have previously been constructed, which resulted in different numbers of chromosomes, one by Han et al. 2021 of a specimen sampled in Heilong River (Raohe county) in the northeastern part of China resulting in n=22 chromosomes [195], and another by Song et al. 2021 resulting in n=24 chromosomes [196]. Combined, these results suggest that there are chromosomal differences between different lineages of burbot. Previous studies using mitochondrial markers point towards at least five major mitochondrial burbot clades across western and northern Europe, Russia, and parts of central Asia, likely reflecting postglacial colonization and the geological history of burbot in separate Holarctic regions [197]. Based on this, we speculate that the differences in chromosomal numbers observed might be linked to true karyotype differences and thus, different postglacial colonization events.

**Long-read mitogenome assemblies for six codfishes**

Complete mitogenomes were assembled for six of the species included (Additional file 2, Fig. S2), using either MitoVGP [134] or MitoHiFi [135] (see Additional file 5). The European hake was an exception, as the mitogenome was found to be fully assembled during the Flye assemblage stage, 16,968 bp in size, as would be expected from a complete mitogenome. Upon inspection, read depth distribution among the assemblies differed markedly (Additional file 2, Fig. S3); however, this is expected as HiFi reads (for European hake and Atlantic cod (NEAC)) are generated from collapsing sequencing reads, leaving fewer but highly accurate reads. MitoFish annotation of the mitogenome assemblies resulted in the identification of 13 mitochondrial protein-coding genes (PCGs) within all assemblies (Additional file 2, Fig. S2). In Arctic cod, the T-P spacer found before the D-Loop has been shown to contain duplicated repeat motifs [198]. The length of this region is known to differ within individuals [198]. Interestingly, the Arctic cod mitogenome assembly was found to include a stretch of repeated sequence located between tRNA-Thr and tRNA-Pro (T-P spacer), and the sequence reads within this region varied with several large indels, an indication that this area could potentially represent mitochondrial heteroplasmy (Additional file 2, Fig. S4).

**References**

60. Kirtiklis L, Kuciński M, Ocalewicz K, Liszewski T, Woźnicki P, Nowosad J, et al. Heterochromatin organization and chromosome mapping of rRNA genes and telomeric DNA sequences in the burbot *Lota lota* (Linnaeus, 1758) (Teleostei: Gadiformes: Lotidae). Caryologia. 2017;70:15–20. https://doi.org/10.1080/00087114.2016.1254453

61. Zhou J, Jiang Y, Zhang L, Wang Y. Karyotype and morphological characteristics analysis of *Lota lota*. Freshw Fish. 2019;49:3–8. https://doi.org/10.13721/j.cnki.dsyy.2019.04.001

134. Formenti G, Rhie A, Balacco J, Haase B, Mountcastle J, Fedrigo O, et al. Complete vertebrate mitogenomes reveal widespread repeats and gene duplications. Genome Biol. 2021;22:120. https://doi.org/10.1186/s13059-021-02336-9

135. Uliano-Silva M, Ferreira JGRN, Krasheninnikova K, Blaxter M, Mieszkowska N, Hall N, et al. MitoHiFi: a python pipeline for mitochondrial genome assembly from PacBio high fidelity reads. BMC Bioinformatics. 2023;24:288. https://doi.org/10.1186/s12859-023-05385-y

195. Han Z, Liu M, Liu Q, Zhai H, Xiao S, Gao T. Chromosome-level genome assembly of burbot (*Lota lota*) provides insights into the evolutionary adaptations in freshwater. Mol Ecol Resour. 2021;21:2022–33. https://doi.org/10.1111/1755-0998.13382

196. Song D, Qian Y, Meng M, Dong X, Lv W, Huo T. Chromosome-Level genome assembly of the burbot (*Lota lota*) using nanopore and Hi-C technologies. Front Genet. 2021;12. https://doi.org/10.3389/fgene.2021.747552

197. Khrunyk YY, Borodin AV, Semerikov VL, Yalkovskaya LE, Koporikov AR, Rakitin SB, et al. First data on genetic diversity of burbot (*Lota lota L*.) in the Western Siberian. Dokl Biochem Biophys. 2015;463:255–8. https://doi.org/10.1134/S160767291504016X

198. Pálsson S, Paulsen J, Árnason E. Rapid evolution of the Intergenic T–P spacer in the mtDNA of Arctic cod *Arctogadus glacialis*. Mar Biotechnol. 2008;10:270–7. https://doi.org/10.1007/s10126-007-9058-5
