# Supplementary material for: Rapid genome modifications including chromosomal fusions and large-scale inversions are key features in Arctic codfish species
Source: Genome Biol. 2026 Feb 16;27:100. doi: 10.1186/s13059-026-03975-6 (PMC13011446; doi:10.1186/s13059-026-03975-6)
Supplement: Supplementary file 4 — Additional file 4: Supplementary note 2. Description of validation of chromosomal fusions and inversions using ONT genome assemblies and reads, and further details on macro-synteny and identification of chromosomal rearrangements in codfishes [48, 49, 53, 54, 62, 193]. [file 13059_2026_3975_MOESM4_ESM.docx]

**Supplementary note 2**

**Validation of chromosomal fusions and inversions using ONT genome assemblies and reads**

We leveraged the ONT draft assembly generated from different specimens than the PacBio reference genomes for both polar cod and Arctic cod to investigate the structural rearrangements identified in the present study (chromosomal fusions) as well as the chromosomal inversions previously identified in Hoff et al. [53] and Maurstad et al [54], in more detail. Using Arctic cod data, we also mapped ONT reads back to the PacBio assembly, and manually inspected the reads surrounding putative fusion boundaries, as well as the breakpoints of the one inversion that was detected between these assemblies.

The independent assemblage of a polar cod genome assembly (from ONT and Hi-C data, stemming from a different individual than the PacBio sample) yielded the same chromosomal fusions as our PacBio reference genome (Additional file 2, Fig. S2 and S3). One exception was observed; this was the fusion on chromosome 3 (Additional file 2, Fig. S2). Here, comparisons between the two assemblies suggest that a larger region at the end of the chromosome in the PacBio assembly was translocated to the beginning of the chromosome in the ONT assembly. This difference between the genomes could be the result of the fusion of different chromosomal arms between these sequenced individuals, or an assembly error in one of the two assemblies. However, looking at the Hi-C contact map for the two assemblies (Additional file 2, Fig. S1 for the PacBio reference genome, not shown for the ONT assembly), it is less likely. Additionally, the Arctic codfishes have been reported to display population-level karyotype variability [48,49], which could explain such large structural rearrangements among individuals in the population.

Of the total 20 chromosomal inversions that were detected in polar cod in Hoff et al. [53] (using population data), we here were able to identify 11 inversions between the PacBio and ONT individuals (Additional file 1, Table S6), providing additional support that these are genuine chromosomal inversions. It should be noted that some of the 11 inversions that were identified between the assemblies varied in length compared to when found using the population data. This is not surprising as inversion boundaries are expected to be dynamic to some degree. In addition, several of the inversions here detected are categorized as “complex type”, and are likely made up of multiple haploblocks, or nested inversions (see Hoff et al. [53] for more details), that could yield different lengths of variable regions depending on the sample that is inspected.

For Arctic cod, similar results regarding the fusions were observed; the draft ONT assembly for Arctic cod also resulted in the same chromosomes as the PacBio assembly, with 15 chromosomes in total, and the eight largest displaying the same fusions as the PacBio assembly (Additional file 2, Fig. S3). For Arctic cod, it is worth noting that the draft ONT assembly was constructed using a reference-guided approach, which likely affects the chromosomal synteny of the assembly. However, when Hi-C data (from the ONT sequenced sample) was mapped back to the ONT assembly, no conflicts were observed, providing support that the chromosomal architecture of the assembly is largely correct. Furthermore, when mapping ONT reads back to the PacBio assembly, we observed ONT reads that spanned a region on Arctic cod chromosome 2 corresponding to the fusion/translocation of the homologous Atlantic cod chromosomes 11 and 6 (see Additional file 2, Fig. S10 for more details), supporting that these regions are continuous in Arctic cod chromosome 2, across the fusion boundary.

We identified one inversion between the Arctic cod PacBio and ONT assembly, which was previously reported by Maurstad et al. [54] on chromosome 6 at 45-47 Mb (using population data). The SyRI analyses detected one larger and two smaller inversions between the assemblies (reported as INV1672, INV1673, and INV1674 by SyRI), that overlap the 45-47 Mb region in the PacBio assembly (Additional file 1, Table S7; Additional file 2, Fig. S3). The population data in Maurstad et al. indicated that the entire region is one inversion, this could mean that either the three inversions identified by SyRI are wrongly subdivided, and should be one large inversion, or the larger inversion reported by the population data is in reality comprised of smaller inversions, which possibly vary among individuals. Only when compared to haplotype 1 of the ONT assembly did we find the larger inverted region (Additional file 2, Fig. S3), while in haplotype 2, the large inversion was not present, and there seems to be a translocation event in the chromosomal region making up the first breakpoint (Additional file 2, Fig. S3A vs. B), indicating that the ONT sequenced sample may be heterozygous for the inversion. Inspecting the ONT reads mapped back to the PacBio assembly, we observed an abrupt break in read alignment where the first breakpoint of the inversion is located (see Additional file 2, Fig. S12, the beginning of the reported INV1672). Further, we see that reads within the beginning of the inversion map, both within and outside the inversion putative end breakpoint, which is the inversion INV1674 (see Additional file 2, Fig. S12). This is likely due to a combination of the minor rearrangements, i.e., translocation, and the fact that the ONT individual is likely heterozygous for the inversion.

**Macro-synteny and identification of chromosomal rearrangements**

While evaluating chromosomal architecture between Atlantic cod (NEAC) [62] and two species outgroups (platyfish *[Xiphophorus maculatus]* and John Dory *[Zeus Faber]*), we found a few larger inter-chromosomal rearrangements. The rearrangements we identified were: Firstly, parts of chromosomes NC_036450.1, NC_036459.1, and NC_036466.1 in platyfish made up a single chromosome, Ne4 in Atlantic cod (Additional file 2, Fig. S7). Secondly, parts of chromosomes NC_036450.1, and 036459.1 in platyfish made up a single chromosome, Ne19 in Atlantic cod (Additional file 2, Fig. S7). Moreover, between Atlantic cod and John Dory, Ne4 in Atlantic cod is made up of parts of three chromosomes in John Dory OY482846.1, OY482861.1, and OY482865.1 (Additional file 2, Fig. S8). Furthermore, Ne19 in Atlantic cod is made up of parts of OY482861.1 and OY482865.1 in John Dory. Additionally, Ne12 and Ne22 in Atlantic cod, made up one chromosome in John Dory. Lastly, John Dory OY482846.1, is made up of parts of Atlantic cod Ne4 and Ne21 (Additional file 2, Fig. S8). Taken together, for most of the chromosomes, chromosomal architecture seems to be fairly conserved in Atlantic cod compared to outgroup species. However, for the rearrangements that are identified, both fissions, fusions, and translocations must have occurred to result in the current chromosomal organization observed in Atlantic cod.

In comparisons carried out between the fish species of the present study, chromosomes Ne4 and Ne19 in Atlantic cod corresponded to one homologous chromosome each in all comparisons, except in European hake where Ne4 in Atlantic cod was found to correspond to parts of Mm1, Mm2, and Mm15 (Additional file 2, Fig. S9A), and in burbot where Atlantic cod Ne4 was found to be homologous to half of Ll23 and a large part of Ll1 (Additional file 2, Fig.s S9B). Our results suggest that the fusion of chromosomes leading to Ne19 in Atlantic cod (and homologous chromosomes in the other species) either took place before the split of Merlucciidae — since there is a 1:1 relationship between the homologous chromosomes of the other species investigated presently, then a subsequent fission event took place in the lineage of Merlucciidae, or a fusion event occurred after the splitting of Merlucciidae, in the lineage of Gadidae and Lotidae. For Atlantic cod Ne4 our results indicate that a fusion event has occurred after the splitting of Lotidae and Merlucciidae, in the lineage of Gadidae. Moreover, syntenic relationships of Mm1 and Mm15 (European hake) compared to the corresponding chromosomes in the other codfishes (See Fig. 2A; Additional file 2, S9A for comparisons to burbot and Atlantic cod, respectively) suggest that complex chromosomal reshuffling characterized by both fissions, fusions and/or translocations likely have taken place specifically in the lineage of European hake, which diverged from the Gadidae ~70 million years ago (see Malmstrøm et al. [193], and Fig. 1).

Moreover, in the full characterization of the chromosomal architecture among the six Gadiform fish species, Arctic and polar cod were found to harbor eight vs. five larger chromosomes, respectively, likely a result of ancestral chromosomal fusions (see Results and Discussion main document for more details). Additionally, we detect that some of the other gadids: the Atlantic haddock, burbot, and European hake also contain one or several large chromosomes, all exceeding 40 Mbp in size (see Fig. 2A). These large chromosomes include Ma1 in Atlantic haddock; Ll12 in burbot; and Mm1, Mm2, and Mm3 in European hake (Fig. 2A; Additional file 2, Fig. S9A, and Fig. S9B). Intriguingly, in comparisons between Atlantic haddock and polar cod, we found that Atlantic haddock Ma1 and polar cod Bs3 — which we identified as being a fused chromosome in polar cod — were 1:1 syntenic (Fig. 2A; Additional file 2, Fig. S9C). Comparisons between Atlantic haddock and Atlantic cod (as well as burbot) show that Ma1 may have originated by a chromosomal insertion event, of which close to the entirety of one ancestral chromosome homologous to Ne15 and Ll19 has been inserted into a chromosome homologous to Ne3 and Ll16 (Fig. 2A; Additional file 2, Fig. S9C and S7D). While for Bs3 in polar cod, an end-to-end fusion of chromosomes homologous to Ne3 and Ne15 seems most likely, with subsequent translocation of a smaller part of the end region of Ne15 to the beginning of Bs3. However, for this fused chromosome (Bs3), an insertion of the entire chromosome homologous to Ne3 into Ne15 cannot be ruled out completely (see Fig. 2A; Additional file 2, Fig. S9C, and S9D, and Results in the main document for more details). From this, we can conclude that even if Ma1 and Bs3 are seemingly 1:1, these fusion events are likely of independent origins in polar cod and Atlantic haddock.

**References**

48. Ghigliotti L, Mazzei F, Christiansen J, Fevolden S, Pisano E. From Antarctic to Arctic polar fishes: First cytogenetic analyses of three gadid species (*Arctogadus glacialis*, *Boreogadus saida* and *Gadus morhua*). Polarnet Tech Rep. 2005;1:69–73.

49. Ghigliotti L, Christiansen JS, Carlig E, Di Blasi D, Pisano E. Latitudinal cline in chromosome numbers of ice cod *A. glacialis* (Gadidae) from Northeast Greenland. Genes. 2020;11:1515. https://doi.org/10.3390/genes11121515

53. Hoff SNK, Maurstad MF, Moan AL, Ravinet M, Pampoulie C, Vieweg I, et al. Genomic rearrangements drive population divergence in a keystone Arctic species with high gene flow. bioRxiv. 2024; https://doi.org/10.1101/2024.06.28.597535

54. Maurstad MF, Hoff SNK, Cerca J, Ravinet M, Bradbury I, Jakobsen KS, et al. Reference genome bias in light of species-specific chromosomal reorganization and translocations. Genome Biol. 2025;26:355. https://doi.org/10.1186/s13059-025-03761-w

62. Jentoft S, Tørresen O, Tooming-Klunderud A, Skage M, Kollias S, Jakobsen K, et al. The genome sequence of the Atlantic cod, *Gadus morhua* (Linnaeus, 1758). Wellcome Open Res. 2025;9:189. https://doi.org/10.12688/wellcomeopenres.21122.2

193. Malmstrøm M, Matschiner M, Tørresen OK, Star B, Snipen LG, Hansen TF, et al. Evolution of the immune system influences speciation rates in teleost fishes. Nat Genet. 2016;48:1204–10. https://doi.org/10.1038/ng.3645
