# Supplementary material for: Rapid genome modifications including chromosomal fusions and large-scale inversions are key features in Arctic codfish species
Source: Genome Biol. 2026 Feb 16;27:100. doi: 10.1186/s13059-026-03975-6 (PMC13011446; doi:10.1186/s13059-026-03975-6)
Supplement: Supplementary file 5 — Additional file 5: Supplementary methods [59, 62, 78, 123–127, 129–135, 137, 138, 140–145, 160, 161, 167–171, 174, 178, 194, 199–224]. [file 13059_2026_3975_MOESM5_ESM.docx]

**Supplementary methods**

**Genome sequencing and library preparation**

***Polar cod, Arctic cod, burbot, and Atlantic cod (NCC)***

DNA was extracted from a variety of tissue samples, depending on what was available for each species (Additional file 1, Table S11). To obtain high molecular DNA for PacBio as well as Illumina paired-end sequencing libraries, DNA isolation was performed using a high-salt extraction protocol (SOP can be found at https://www.mn.uio.no/cees/english/people/researcher-postdoc/jentoft/sop-038-high-salt-dna-extraction.pdf).

PacBio sequencing libraries were prepared using the PacBio 20 kb library preparation protocol (PacBio). Fragmentation of DNA was performed using Megaruptor (Diagenode), and size selection was done using BluePippin (Sage Science) with a cut-off length of 10 kb. Libraries were sequenced on a Pacific Biosciences Sequel II instrument (PacBio), using Sequel Polymerase v2.0 and Sequencing Chemistry v.2.1, and loading on the instrument was performed by diffusion. The libraries were sequenced across 12, 11, and 10 SMRT cells, for polar cod, Arctic cod, and burbot, respectively, with a movie time of 600 minutes.

The Atlantic cod (NCC) genome was sequenced across three rounds and on three PacBio, where the first two libraries were sequenced on Pacific Biosciences RS II instrument using P6-C4 chemistry. A total of 28 SMRT cells were used for sequencing, 15 SMRT cells were sequenced with 240 min movie time, and 5 SMRT cells were sequenced using 360 min movie time. Additionally, eight additional SMRT cells (with a movie time of 600 minutes) were sequenced using the Sequel instrument (PacBio), using Sequel Polymerase v2.0, and Sequencing Chemistry v.2.0 (Additional file 1, Table S2).

For polar cod, Arctic cod, and burbot preparation of Illumina 150 bp paired-end (PE) libraries were done using KAPA HyperPrep Plus (Roche), with 4 cycles of PCR. Sequencing was performed across 3 lanes, using the Illumina Hiseq4000 instrument (Illumina). For polar cod, Arctic cod, and Atlantic cod, high molecular DNA for 10X library preparation was isolated from gill or spleen (Additional file 1, Table S11) using the Nanobind CBB BIG DBA kit (Circulomics Inc.). 10X library preparation was performed following the Chromium Genome Reagent kit V2 (10X Genomics) user guide using the 10X listed kits as well as suggested QC kits and methods including Chromium™ Genome Chip Kit v2, 48 rxns, Chromium™ Genome Library Kit & Gel Bead Kit v2, 16 rxns, Chromium™ Genome Library Kit V2, 16 rxns -120255, Chromium™ Genome Gel Bead, 16 rxns -120214, and Chromium™ i7 Multiplex Kit, 96 rxns. The 10X libraries were sequenced as 150 bp paired-end reads on the Illumina HiseqX Instrument (Illumina). For polar cod, Arctic cod, and Atlantic cod (NCC), gill filaments and spleen were used as input tissue (Additional file 1, Table S11) with the Arima Hi-C Kit (Arima Genomics) to obtain crosslinked and proximally ligated DNA-PLD. For burbot, Omni-C (Dovetail Genomics) was utilized for crosslinking DNA. 150 bp paired-end Hi-C library preparation was performed using KAPA Hyper Prep Kit (Roche), creating an Illumina library from the PLD, and sequenced on the Illumina HiseqX Instrument (Illumina).

***European hake***

Isolation of DNA from European hake was done from blood, using the nucleated blood protocol by the Circulomics Nanobind BIG DNA kit (Circulomics Inc.). The PacBio libraries were prepared using the Pacific Biosciences protocol for HiFi library prep using SMRTbell® Express Template Prep Kit 2.0. Two libraries were prepared, and 7.5 µg DNA was fragmented into 15-20 kb fragments using Megaruptor 3. A total of 5 µg of fragmented DNA was used for library prep. Final library was size selected using BluePippin with a 10 kb cut-off. Library was sequenced on two 8M SMRT cells on a Sequel II instrument using Sequel II Binding kit 2.2 and Sequencing chemistry v2.0. Loading was performed by adaptive loading, with a movie time of 30 hours. Hi-C preparation was done using the Dovetail Omni-C kit provided by Dovetail genomics. Library preparation followed the “Omni-C proximity Ligation Assay, version 1.0”. The final library was sequenced on an S4 flow cell utilizing the 2*150 bp PE mode on an Illumina Novaseq sequencer. DNA isolation, HiFi sequencing, and Hi-C sequencing were performed by the Norwegian Sequencing Centre (NSC).

***Atlantic haddock***

Isolation of DNA from haddock was done from blood (Additional file 1, Table S11), using the Nanobind HMW Tissue DNA kit (Circulomics Inc.). Library was prepared using Pacific Biosciences Express library preparation protocol without any fragmentation of the sample prior to library prep. Size selection of the final library was performed using BluePippin using 15 kb cut-off. The library was sequenced on 6 SMRT cells using Sequel II Binding kit 2.0 and Sequencing chemistry v2.0 with 15 hours movie time. The Hi-C library was prepared following instructions in the Arima library user guide. Around 4.4µg proximally ligated DNA was sheared using Covaris tubes on a Covaris E220 instrument. Following size selection, 383ng was used in the biotin enrichment step. Illumina unique indexing adaptors were used for the ligation. Library was amplified with 6 cycles of PCR, purified, and checked on a fragment analyzer (FA) using the NGS kit. Library concentration was controlled with qPCR (KAPA Quantification kit). An additional Illumina library was prepared based on the isolated DNA from the Circulomics DNA protocol, with 1000ng gDNA as input in the Kapa Hyper prep PCR-free workflow. The same quality checks as for Hi-C were performed here.

***De novo* genome assembly for six Gadiform fishes**

To construct the primary assembly for Arctic cod, Atlantic cod (NCC), and burbot, PacBio Sequel long reads were assembled using Flye v2.4 [123]. The option “minimum overlap” was set to 2.5 kbp for all three primary assemblies. The presence of duplicated contigs was evaluated using purge dups v.1.0.1 [199], and removal of duplicated contigs in the primary assembly of Arctic cod, as well as burbot, was performed using purge dups. In order to scaffold the primary contigs into chromosome-length scaffolds, we leveraged a combination of 10X linked and Arima Hi-C linked Illumina PE sequencing data for Arctic cod and Atlantic cod (NCC).

To scaffold the primary assembly of Arctic cod and Atlantic cod (NCC), Illumina reads from a 10X sequencing library were aligned to the primary assemblies scaffolded/linked using Scaff10X v4.2 [124]. Subsequently, Illumina Hi-C sequence data were mapped to the 10X scaffolded assembly using Juicer v1.5.6 [200]. For the burbot, Hi-C OmniC Illumina PE reads were mapped directly onto the Flye primary assembly using bwa mem v0.7.17 [160] and scaffolded by Juicer v1.6 [200].

Contigs could then be grouped, arranged, and anchored to near chromosomal length scaffolds using 3D-DNA v180922 [125]. The 3D-DNA draft assemblies and Hi-C contact maps for each species produced by Juicer were visualized and manually inspected in the Juicebox program suite v1.11.08 [127]. During manual curation of Hi-C contact maps, we discovered two obviously erroneous linked super-scaffolds within the Atlantic cod (NCC) as well as the burbot assembly, which had low Hi-C linkage between them, and which likely make up two separate chromosomes. These were manually split into two super-scaffolds, and we used the 3D-DNA post-review script to correct the assemblies (https://github.com/aidenlab/3d-dna/blob/master/run-asm-pipeline-post-review.sh). After scaffolding the genome assemblies for all three species, we performed additional rounds of polishing to close any remaining gaps and improve the base-level accuracy of the assemblies. For long-read polishing, we utilized PacBio sequencing data and aligned the reads to the Hi-C scaffolds using pbmm2 v1.2.1 [129]. Polishing was performed using gcpp v1.9.0 [129]. Lastly, two rounds of short-read polishing were performed by aligning Illumina paired-end reads with Minimap v2.155 [130] and subsequent base calling, done using Freebayes v1.3.2 [131]. Finalized genome assemblies were evaluated using metrics calculated by the Assemblathon_stats [132] script. The completeness of each assembly was assessed using the Benchmarking Universal Single-Copy Orthologs (BUSCO) software v5.0.0 [133] as well as by aligning and visualizing the final assemblies against the chromosome-level assembly of Atlantic cod (gadMor3.0) [62] using the D-GENIES homepage [137].

The European hake genome was assembled using a combination of PacBio HiFi long reads and Hi-C data. First, HiFi reads were assembled into a primary assembly using Flye v2.9 [123] with default settings, and the parameter genome size was set to 650 Mb. HiFi reads were then mapped against the assembly using Minimap2 v2.17 [130] to generate a read-depth histogram to assess duplication levels. Purge Haplotigs v1.1.2 [201] was used to identify and purge any potentially duplicated contigs. Reads were flagged and purged using the read-depth information from the mapping step, and cutoffs were manually selected based on the read-depth histogram. Following, the purged Flye draft assembly was scaffolded using Juicer v1.22.01 [200] and the 3D-DNA pipeline [125] by first aligning Hi-C data to the purged primary assembly using the Juicer pipeline. Next, the alignment file was run through the 3D-DNA pipeline to produce a candidate near chromosome-length draft genome assembly. Thereafter, the draft genome assembly was visualized using Juicebox v1.11.08 [127] and manually curated for any misassemblies. Manual curation included splitting one super-scaffold into two, due to weak Hi-C contact points between scaffolds. The manually curated assembly was processed through the 3D-DNA pipeline one last time, incorporating the changes made within Juicebox.

Atlantic haddock was assembled using a combination of PacBio long reads and chromosome confirmation Hi-C reads. The PacBio reads were first assembled using Flye v2.9 with default settings. The PacBio reads were mapped to the Flye draft assembly with minimap v2.22, and then purge_dups v1.2.5 was applied. Next, Hi-C reads were mapped against the purged genome assembly with bwa mem v0.7.17 with the options -5SPM. SAMtools v1.11 [161] was used to deduplicate the mapped reads. YaHS v1.1a [126] was used to scaffold the purged genome assembly. Next, pbmm2 v1.9 was used to map the PacBio reads to the scaffolded assembly, and gcpp v1.0.0 was used to polish the assembly. Further, two rounds of polishing using Illumina reads mapped with bwa mem v0.7.17 and processed with Freebayes v1.3.6. FCS-Adaptor v0.2.2 (<https://github.com/ncbi/fcs>) was run on the polished assembly, and any adaptor sequences found were masked using BEDtools v2.30.0 maskfasta [202]. FCS-GX v0.2.2 (<https://github.com/ncbi/fcs>) was used to search for contamination. If a contaminant was found at the start or end of a sequence, the sequence was trimmed using a combination of SAMtools faidx [161], BEDtools complement, and BEDtools getfasta. If the contaminant was internal, it was masked using BEDtools maskfasta. The assembly was manually curated using the GRIT rapid curation suite [203] and the PretextView v.0.2.5 (<https://github.com/wtsi-hpag/PretextView>, last accessed September 27, 2023). Genome assembly metrics were calculated using the Assemblathon_stats [132] script, completeness of each assembly was assessed using the Benchmarking Universal Single-Copy Orthologs (BUSCO) software v5.0.0 [133] as well as by aligning and visualizing the final assemblies against the chromosome-level assembly of Atlantic cod (gadMor3.0) [62] using the D-GENIES homepage [137].

**RNA sequencing for annotation**

Total RNA was extracted from spleen, liver, and gonad for polar cod and Arctic cod. All tissues were stored at -80 °C from collection till arrival and processing at University of Oslo, Norway. RNA isolation was performed using the RNeasy mini kit (Qiagen) following the manufacturer's instruction. RNA isolate was normalized, and 2µg was used as input for the Illumina TruSeq mRNA stranded kit (Illumina) and prepped on a Perkin Elmer Sciclone NGSx liquid handler system (Perkin Elmer). Samples were indexed using Uniqued dual indexing. Final libraries were QCed on a fragment analyzer system using the standard sensitivity NGS kit from AATI (Agilent) and quantified using the KAPA Library quantification kit for Illumina (Roche). The library pool was sequenced on an Illumina Hiseq4000 instrument using 2*150bp PE read mode (Illumina).

**Genome annotation**

***Norwegian coastal cod (NCC), Arctic cod, and polar cod***

A putative library of repeated elements prior to gene annotation was created as described in Tørresen et al. (2017) [204]. Briefly, RepeatModeler v1.0.8 [205], LTRharvest [206], part of genometools v1.5.7 [207], and TransposonPSI [208] were used in combination to create a set of putative repeats. Elements with a match only against an UniProtKB/SwissProt database [209] and not against the database of known repeated elements included in RepeatMasker were removed. The remaining elements were classified and combined with known repeat elements from RepBase v20150807 [210].

HISAT v2.1.0 [139] was used to map all RNA-seq data to the assembly. For NCC we downloaded the following datasets from SRA: PRJEB12487, PRJEB31396, PRJNA256972, PRJNA277848, and PRJNA277848. Portcullis v1.2.0 [140] was run on the mapped RNA-seq reads to generate a catalog of good junctions. These junctions were used in a second round of HISAT2. Mikado v2.0rc6 [141] created a set of the best transcripts from the second round of HISAT2. Predicted proteins from zebrafish (*Danio rerio*), Atlantic cod (gadMor1 and gadMor3), channel bull blenny (*Cottoperca gobio*), herring (*Clupea harengus*), Northern pike (*Esox lucius*), denticle herring (*Denticeps clupeoides*), and *Triplophysa tibetana* were downloaded and used in ProtHint v2.2.0 [211] to generate hints for BRAKER. BRAKER v2.1.5 [212] was run with the hints from ProtHint and mapped reads from the second round of HISAT2. GenomeThreader v1.7.1 [213] was used to map UniProtKB/Swiss-Prot Release 2019_10 [209] of 13-Nov-2019 proteins to the genome assembly. The funannotate v1.7.0 [214] mask command was used to mask the genome assembly with the repeat library, before funannotate train was run with all RNA-seq data to train AUGUSTUS v3.3.3 [215]. The training results from funannotate train, proteins from the fishes and UniProtKB/Swiss-Prot, GeneMark gene models from BRAKER, and gene models from Mikado and GenomeThreader were used as input to funannotate predict. InterProScan v5.34-73.0 [145] was run on the predicted proteins from funannotate predict, and funannotate annotate was used to integrate those results into a final predicted set of genes.

***Atlantic haddock, burbot, and European hake***

AGAT v1.0 [144] agat_sp_keep_longest_isoform.pl and agat_sp_extract_sequences.pl were used on the zebrafish assembly and annotation to generate one protein (the longest isoform) per gene. Miniprot v0.5 [142] was used to align the proteins to the curated assemblies. UniProtKB/Swiss-Prot release 2022_03 [209], in addition to the vertebrata part of OrthoDB v10 [216], were also aligned separately to the assemblies. RED v2018.09.10 was run via redmask (https://github.com/nextgenusfs/redmask) on the assemblies to mask repetitive areas [217]. GALBA (https://github.com/Gaius-Augustus/GALBA, commit: f4aaeca) was run with the zebrafish proteins using the miniprot mode on the masked assemblies [142,218–221]. The funannotate-runEVM.py script from Funannotate v1.8.13 [138] was used to run EvidenceModeler v1.1.1 [143] on the alignments of zebrafish proteins, UniProtKB/Swiss-Prot proteins, vertebrata proteins, and the predicted genes from GALBA. The resulting predicted proteins were compared to the protein repeats that funannotate distributes using DIAMOND v2.0.15 [219] blastp, and the predicted genes were filtered based on this comparison using AGAT. The filtered proteins were compared to the UniProtKB/Swiss-Prot release 2022_03 using DIAMOND blastp to find gene names, and InterProScan v5.47-82 [145] was used to discover functional domains. AGATs [144] agat_sp_manage_functional_annotation.pl was used to attach the gene names and functional annotations to the predicted genes.

***New annotation of all species***

To better facilitate gene family comparisons between the species, we redid the annotation. The annotation itself is identical to the one described above, using a pre-release version of the EBP-Nor genome annotation pipeline (https://github.com/ebp-nor/GenomeAnnotation), but updated input data. The actinopterygii part of OrthoDB v12 [222] was used instead of the vertebrata part of OrthoDB v10. UniProtKB/Swiss-Prot proteins were updated to release 2025_03. The following programs were updated compared to the description above: AGAT v1.4, miniprot v0.13, EvidenceModeler v2.1.0, funannotate v1.8.17, DIAMOND v2.1.8, GALBA v1.0.9, and InterProScan v5.62-94.

***Long-read assembly of mitochondrial genomes***

Mitochondrial genomes (mitogenomes) were generated using either of two methods, depending on the input data available. Species sequenced using PacBio continuous long reads (CLR) and which had Illumina PE short reads available were assembled using the mitoVGP v2.0 pipeline [134]. The MitoHiFi v2.2 [135] pipeline was applied to assemble the mitogenomes of the Atlantic cod (NEAC) straight from HiFi data. MitoHiFi can either assemble mitogenomes from raw HiFi reads or search already assembled contigs to extract potential mitogenomes. In addition to the assembly from raw reads, the European hake draft purged Flye assembly was used to search for and retrieve any potentially assembled mitogenomes. Raw reads that map to the mitogenomes are extracted as part of the assembly pipeline of MitoHiFi and MitoVGP. These reads were mapped back to the assembled mitogenomes using pbmm2 v1.9.0 [129], a Minimap2 SMRT wrapper for PacBio data, to assess read depth distribution, and read depth was measured per base using SAMtools v1.14 [161]. The mitogenomes were afterward annotated and visualized using the online tool MitoFish v3.74 [167,168]. Lastly, after assembly and annotation, mitogenomes for all seven species were manually inspected using the Integrative Genomics Viewer (IGV) v2.9.4 [178].

**Phylogenomic placement of Arctic cod**

***Mitochondrial phylogeny***

For the codfishes with sequenced genomes in this study, except for Atlantic haddock, which was accessed from NCBI accession: NC_007396.1, mitochondrial protein-coding genes (PCGs) identified by MitoFish were aligned using MAFFT v7.453 [169] and manually inspected and corrected for reading frame shifts before they were concatenated with PhyKIT v1.11.7 create_concat [170] to produce a supermatrix. A maximum likelihood (ML) tree was inferred using IQ-Tree2 v2.2.0 [59], and ModelFinder Plus (MFP) [171] was used to search for the best substitution model under the Bayesian information criterion (BIC). Branch support was calculated using 1,000 replicates of ultrafast bootstrap approximation (UFBoot) [78].

A Bayesian approach to tree inference was done in BEAST v2.6.7 [194] using the same concatenated supermatrix. BEAST Model Test (bmodeltest) [223] was used to infer the substitution model from the named Extended list of substitution models. A strict clock was applied using the birth-death model as prior, and the analysis was run with a chain length of 100,000,000, with sampling done every 1,000 iterations for a total of 100,000 trees. Convergence was assessed using Tracer v1.7.2 [224], and TreeAnnotator was used to produce the consensus tree with a burn-in of 10% and the target tree set to maximum clade credibility.

Additionally, phylogenetic analysis was conducted using the complete mitogenomes to compare the effects of including all data vs only the PCGs on topology inference. MitoFish outputs reordered mitogenomes, i.e., all start at the same position (tRNA-phe). Therefore, MitoFish annotated mitogenomes were aligned using MAFFT v7.453 [169], and gaps were trimmed using ClipKIT v1.3.0 [174] with the smart-gap approach. The smart-gap strategy was selected because it should work better with alignments that contain taxa that span both shallow and deep evolutionary timescales, appropriate for the taxa included in this study (ClipKIT documentation:https://jlsteenwyk.com/ClipKIT/performance_assessment/index.html#smart-gap). Tree inference for the complete mitogenomes followed the same steps as the PCGs for both ML and Bayesian analysis.

**References**

59. Minh BQ, Schmidt HA, Chernomor O, Schrempf D, Woodhams MD, von Haeseler A, et al. IQ-TREE 2: New models and efficient methods for phylogenetic inference in the genomic era. Mol Biol Evol. 2020;37:1530–4. https://doi.org/10.1093/molbev/msaa015

62. Jentoft S, Tørresen O, Tooming-Klunderud A, Skage M, Kollias S, Jakobsen K, et al. The genome sequence of the Atlantic cod, *Gadus morhua* (Linnaeus, 1758). Wellcome Open Res. 2025;9:189. https://doi.org/10.12688/wellcomeopenres.21122.2

78. Hoang DT, Chernomor O, von Haeseler A, Minh BQ, Vinh LS. UFBoot2: Improving the ultrafast bootstrap approximation. Mol Biol Evol. 2018;35:518–22. https://doi.org/10.1093/molbev/msx281

123. Kolmogorov M, Yuan J, Lin Y, Pevzner PA. Assembly of long, error-prone reads using repeat graphs. Nat Biotechnol. 2019;37:540–6. https://doi.org/10.1038/s41587-019-0072-8

124. Scaff10X v5.0: Pipeline for scaffolding and breaking a genome assembly using 10x genomics linked-reads. wtsi-hpag; 2023 [cited 2023 Dec 15]. https://github.com/wtsi-hpag/Scaff10X.

125. Dudchenko O, Batra SS, Omer AD, Nyquist SK, Hoeger M, Durand NC, et al. De novo assembly of the *Aedes aegypti* genome using Hi-C yields chromosome-length scaffolds. Science. 2017;356:92–5. https://doi.org/10.1126/science.aal3327

126. Zhou C, McCarthy SA, Durbin R. YaHS: yet another Hi-C scaffolding tool. Bioinformatics. 2023;39:btac808. https://doi.org/10.1093/bioinformatics/btac808

127. Robinson JT, Turner D, Durand NC, Thorvaldsdóttir H, Mesirov JP, Aiden EL. Juicebox.js provides a cloud-based visualization system for Hi-C ata. Cell Syst. 2018;6:256-258.e1. https://doi.org/10.1016/j.cels.2018.01.001

129. GitHub - PacificBiosciences/pbbioconda: PacBio secondary analysis tools on Bioconda. Contains list of PacBio packages available via conda. [cited 2023 Dec 15]. https://github.com/PacificBiosciences/pbbioconda.

130. Li H. Minimap2: pairwise alignment for nucleotide sequences. Bioinformatics. 2018;34:3094–100. https://doi.org/10.1093/bioinformatics/bty191

131. Garrison E, Marth G. Haplotype-based variant detection from short-read sequencing. arXiv; 2012 [cited 2023 Jan 2]. https://doi.org/10.48550/arXiv.1207.3907

132. Bradnam KR, Fass JN, Alexandrov A, Baranay P, Bechner M, Birol I, et al. Assemblathon 2: evaluating de novo methods of genome assembly in three vertebrate species. GigaScience. 2013;2:10. https://doi.org/10.1186/2047-217X-2-10

133. Manni M, Berkeley MR, Seppey M, Simão FA, Zdobnov EM. BUSCO update: novel and streamlined workflows along with broader and deeper phylogenetic coverage for scoring of eukaryotic, prokaryotic, and viral genomes. Mol Biol Evol. 2021;38:4647–54. https://doi.org/10.1093/molbev/msab199

134. Formenti G, Rhie A, Balacco J, Haase B, Mountcastle J, Fedrigo O, et al. Complete vertebrate mitogenomes reveal widespread repeats and gene duplications. Genome Biol. 2021;22:120. https://doi.org/10.1186/s13059-021-02336-9

135. Uliano-Silva M, Ferreira JGRN, Krasheninnikova K, Blaxter M, Mieszkowska N, Hall N, et al. MitoHiFi: a Python pipeline for mitochondrial genome assembly from PacBio high-fidelity reads. BMC Bioinformatics. 2023; 24:288. https://doi.org/10.1186/s12859-023-05385-y

137. Cabanettes F, Klopp C. D-GENIES: dot plot large genomes in an interactive, efficient and simple way. PeerJ. 2018;6:e4958. https://doi.org/10.7717/peerj.4958

138. Palmer JM, Stajich J. Funannotate v1.8.1: Eukaryotic genome annotation. Zenodo; 2020 [cited 2024 Jan 19]. https://doi.org/10.5281/zenodo.4054262

140. Mapleson D, Venturini L, Kaithakottil G, Swarbreck D. Efficient and accurate detection of splice junctions from RNA-seq with Portcullis. GigaScience. 2018;7:giy131. https://doi.org/10.1093/gigascience/giy131

141. Venturini L, Caim S, Kaithakottil GG, Mapleson DL, Swarbreck D. Leveraging multiple transcriptome assembly methods for improved gene structure annotation. GigaScience. 2018;7:giy093. https://doi.org/10.1093/gigascience/giy093

142. Li H. Protein-to-genome alignment with miniprot. Bioinformatics. 2023;39:btad014. https://doi.org/10.1093/bioinformatics/btad014

143. Haas BJ, Salzberg SL, Zhu W, Pertea M, Allen JE, Orvis J, et al. Automated eukaryotic gene structure annotation using EVidenceModeler and the Program to Assemble Spliced Alignments. Genome Biol. 2008;9:R7. https://doi.org/10.1186/gb-2008-9-1-r7

144. NBISweden/AGAT: AGAT-v1.2.0. Zenodo; 2023 [cited 2023 Dec 1]; https://doi.org/10.5281/zenodo.8178877

145. Jones P, Binns D, Chang H-Y, Fraser M, Li W, McAnulla C, et al. InterProScan 5: genome-scale protein function classification. Bioinformatics. 2014;30:1236–40. https://doi.org/10.1093/bioinformatics/btu031

160. Li H, Durbin R. Fast and accurate short read alignment with Burrows-Wheeler transform. Bioinformatics 2009;25:1754–60. https://doi.org/10.1093/bioinformatics/btp324

161. Danecek P, Bonfield JK, Liddle J, Marshall J, Ohan V, Pollard MO, et al. Twelve years of SAMtools and BCFtools. GigaScience. 2021;10:giab008. https://doi.org/10.1093/gigascience/giab008

167. Iwasaki W, Fukunaga T, Isagozawa R, Yamada K, Maeda Y, Satoh TP, et al. MitoFish and MitoAnnotator: A mitochondrial genome database of fish with an accurate and automatic annotation pipeline. Mol Biol Evol. 2013;30:2531–40. https://doi.org/10.1093/molbev/mst141

168. Sato Y, Miya M, Fukunaga T, Sado T, Iwasaki W. MitoFish and MiFish pipeline: A mitochondrial genome database of fish with an analysis pipeline for environmental DNA metabarcoding. Mol Biol Evol. 2018;35:1553–5. https://doi.org/10.1093/molbev/msy074

169. Katoh K, Standley DM. MAFFT multiple sequence alignment software version 7: Improvements in performance and usability. Mol Biol Evol. 2013;30:772–80. https://doi.org/10.1093/molbev/mst010

170. Steenwyk JL, Buida TJ III, Labella AL, Li Y, Shen X-X, Rokas A. PhyKIT: a broadly applicable UNIX shell toolkit for processing and analyzing phylogenomic data. Bioinformatics. 2021;37:2325–31. https://doi.org/10.1093/bioinformatics/btab096

171. Kalyaanamoorthy S, Minh BQ, Wong TKF, von Haeseler A, Jermiin LS. ModelFinder: fast model selection for accurate phylogenetic estimates. Nat Methods. 2017;14:587–9. https://doi.org/10.1038/nmeth.4285

174. Steenwyk JL, Iii TJB, Li Y, Shen X-X, Rokas A. ClipKIT: A multiple sequence alignment trimming software for accurate phylogenomic inference. PLOS Biol. 2020;18:e3001007. https://doi.org/10.1371/journal.pbio.3001007

178. Thorvaldsdóttir H, Robinson JT, Mesirov JP. Integrative Genomics Viewer (IGV): high-performance genomics data visualization and exploration. Brief Bioinform. 2013;14:178–92. https://doi.org/10.1093/bib/bbs017

194. Bouckaert R, Vaughan TG, Barido-Sottani J, Duchêne S, Fourment M, Gavryushkina A, et al. BEAST 2.5: An advanced software platform for Bayesian evolutionary analysis. PLOS Comput Biol. 2019;15:e1006650. https://doi.org/10.1371/journal.pcbi.1006650

199. Guan D. Purge_Dups: haplotypic duplication identification tool. 2023 [cited 2023 Dec 15]. https://github.com/dfguan/purge_dups.

200. Durand NC, Shamim MS, Machol I, Rao SSP, Huntley MH, Lander ES, et al. Juicer provides a one-click system for analyzing loop-resolution Hi-C experiments. Cell Syst. 2016;3:95–8. https://doi.org/10.1016/j.cels.2016.07.002

201. Roach MJ, Schmidt SA, Borneman AR. Purge Haplotigs: allelic contig reassignment for third-gen diploid genome assemblies. BMC Bioinformatics. 2018;19:460. https://doi.org/10.1186/s12859-018-2485-7

202. Quinlan AR, Hall IM. BEDTools: a flexible suite of utilities for comparing genomic features. Bioinformatics. 2010;26:841–2. https://doi.org/10.1093/bioinformatics/btq033

203. Howe K, Chow W, Collins J, Pelan S, Pointon D-L, Sims Y, et al. Significantly improving the quality of genome assemblies through curation. GigaScience. 2021;10:giaa153. https://doi.org/10.1093/gigascience/giaa153

204. Tørresen OK, Star B, Jentoft S, Reinar WB, Grove H, Miller JR, et al. An improved genome assembly uncovers prolific tandem repeats in Atlantic cod. BMC Genomics. 2017;18:95. https://doi.org/10.1186/s12864-016-3448-x

205. Flynn JM, Hubley R, Goubert C, Rosen J, Clark AG, Feschotte C, et al. RepeatModeler2 for automated genomic discovery of transposable element families. Proc Natl Acad Sci. 2020;117:9451–7. https://doi.org/10.1073/pnas.1921046117

206. Ellinghaus D, Kurtz S, Willhoeft U. LTRharvest, an efficient and flexible software for de novo detection of LTR retrotransposons. BMC Bioinformatics. 2008;9:18. https://doi.org/10.1186/1471-2105-9-18

207. Gremme G, Steinbiss S, Kurtz S. GenomeTools: A comprehensive software library for efficient processing of structured genome annotations. IEEE/ACM Trans Comput Biol Bioinform. 2013;10:645–56. https://doi.org/10.1109/TCBB.2013.68

208. TransposonPSI: An application of PSI-Blast to mine (retro-)transposon ORF homologies. [cited 2023 Dec 15]. https://transposonpsi.sourceforge.net./.

209. The UniProt Consortium. UniProt: the Universal Protein Knowledgebase in 2023. Nucleic Acids Res. 2023;51:D523–31. https://doi.org/10.1093/nar/gkac1052

210. Jurka J, Kapitonov VV, Pavlicek A, Klonowski P, Kohany O, Walichiewicz J. Repbase Update, a database of eukaryotic repetitive elements. Cytogenet Genome Res. 2005;110:462–7. https://doi.org/10.1159/000084979

211. Brůna T, Lomsadze A, Borodovsky M. GeneMark-EP+: eukaryotic gene prediction with self-training in the space of genes and proteins. NAR Genomics Bioinforma. 2020;2:lqaa026. https://doi.org/10.1093/nargab/lqaa026

212. Brůna T, Hoff KJ, Lomsadze A, Stanke M, Borodovsky M. BRAKER2: automatic eukaryotic genome annotation with GeneMark-EP+ and AUGUSTUS supported by a protein database. NAR Genomics Bioinforma. 2021;3:lqaa108. https://doi.org/10.1093/nargab/lqaa108

213. Gremme G, Brendel V, Sparks ME, Kurtz S. Engineering a software tool for gene structure prediction in higher organisms. Inf Softw Technol. 2005;47:965–78. https://doi.org/10.1016/j.infsof.2005.09.005

214. nextgenusfs/funannotate: funannotate v1.7.0. Zenodo; 2019 [cited 2023 Dec 1]; https://doi.org/10.5281/zenodo.3534297

215. Stanke M, Diekhans M, Baertsch R, Haussler D. Using native and syntenically mapped cDNA alignments to improve de novo gene finding. Bioinformatics. 2008;24:637–44. https://doi.org/10.1093/bioinformatics/btn013

216. Kriventseva EV, Kuznetsov D, Tegenfeldt F, Manni M, Dias R, Simão FA, et al. OrthoDB v10: sampling the diversity of animal, plant, fungal, protist, bacterial and viral genomes for evolutionary and functional annotations of orthologs. Nucleic Acids Res. 2019;47:D807–11. https://doi.org/10.1093/nar/gky1053

217. Girgis HZ. Red: an intelligent, rapid, accurate tool for detecting repeats de-novo on the genomic scale. BMC Bioinformatics. 2015;16:227. https://doi.org/10.1186/s12859-015-0654-5

218. Stanke M, Schöffmann O, Morgenstern B, Waack S. Gene prediction in eukaryotes with a generalized hidden Markov model that uses hints from external sources. BMC Bioinformatics. 2006;7:62. https://doi.org/10.1186/1471-2105-7-62

219. Buchfink B, Xie C, Huson DH. Fast and sensitive protein alignment using DIAMOND. Nat Methods. 2015;12:59–60. https://doi.org/10.1038/nmeth.3176

220. Brůna T, Li H, Guhlin J, Honsel D, Herbold S, Stanke M, et al. Galba: genome annotation with miniprot and AUGUSTUS. BMC Bioinformatics. 2023;24:327. https://doi.org/10.1186/s12859-023-05449-z

221. Hoff KJ, Stanke M. Predicting genes in single genomes with AUGUSTUS. Curr Protoc Bioinforma. 2019;65:e57. https://doi.org/10.1002/cpbi.57

222. Tegenfeldt F, Kuznetsov D, Manni M, Berkeley M, Zdobnov EM, Kriventseva EV. OrthoDB and BUSCO update: annotation of orthologs with wider sampling of genomes. Nucleic Acids Res. 2025;53:D516–22. https://doi.org/10.1093/nar/gkae987

223. Bouckaert RR, Drummond AJ. bModelTest: Bayesian phylogenetic site model averaging and model comparison. BMC Evol Biol. 2017;17:42. https://doi.org/10.1186/s12862-017-0890-6

224. Rambaut A, Drummond AJ, Xie D, Baele G, Suchard MA. Posterior summarization in Bayesian phylogenetics using Tracer 1.7. Syst Biol. 2018;67:901–4. https://doi.org/10.1093/sysbio/syy032
